# Will reshoring manufacturing of advanced electric vehicle battery support renewable energy transition and climate targets?

**DOI:** 10.1126/sciadv.adg6740

**Published:** 2023-06-14

**Authors:** Apoorv Lal, Fengqi You

**Affiliations:** ^1^ Robert Frederick Smith School of Chemical and Biomolecular Engineering, Cornell University, Ithaca, NY 14853, USA.; ^2^ Systems Engineering, Cornell University, Ithaca, NY 14853, USA.; ^3^ Cornell Atkinson Center for Sustainability, Cornell University, 340 Tower Road, Ithaca, NY 14853, USA.

## Abstract

Recent global logistics and geopolitical challenges draw attention to the potential raw material shortages for electric vehicle (EV) batteries. Here, we analyze the long-term energy and sustainability prospects to ensure a secure and resilient midstream and downstream value chain for the U.S. EV battery market amid uncertain market expansion and evolving battery technologies. With current battery technologies, reshoring and ally-shoring the midstream and downstream EV battery manufacturing will reduce the carbon footprint by 15% and energy use by 5 to 7%. While next-generation cobalt-free battery technologies will achieve up to 27% carbon emission reduction, transitioning to 54% less carbon-intensive blade lithium iron phosphate may diminish the mitigation benefits of supply chain restructuring. Our findings underscore the importance of adopting nickel from secondary sources and nickel-rich ores. However, the advantages of restructuring the U.S. EV battery supply chain depend on projected battery technology advancements.

## Teaser

Decarbonization from US EV battery supply chain reshoring can be accelerated by Co-free technologies and expanding EV market.

## INTRODUCTION

Transportation ranks as the second-largest carbon-emitting sector after electricity and heat generation, with greenhouse gas emissions ballooning by 40% in the past two decades (*1*). Light-duty vehicles alone account for 46% of CO_2_ emissions within the transportation sector, necessitating decarbonization to achieve global and national climate change mitigation targets. As a result, numerous countries advocate alternative fuel vehicles, especially electric vehicles (EVs), to decarbonize the light-duty vehicle sector (*2*). Despite the pandemic-induced decline in car sales, EV registration continued to rise and reached over 10 million through 2020, representing 1% of the global passenger car stock (*3*). However, concerns remain over the embodied emissions of EVs and potential shortages of critical materials, primarily centered around battery manufacturing (*4*–*6*). Previous studies showed that battery manufacturing accounted for between 26 and 46% of the embodied emission of EVs (*7*–*10*), emphasizing the critical role of efficient battery recycling and technological advancements in fostering a sustainable raw material supply for EVs (*11*–*13*). Moreover, the coronavirus disease 2019 disruptions severely interrupted supplies of EV battery materials as hundreds of mines, smelters, and refineries suspended their operations (*14*, *15*). Consequently, some western countries are incorporating geopolitical considerations into their efforts to rebuild domestic industrial bases for lithium-ion batteries (LIBs) and gradually reduce dependence on imports (*16*, *17*). This shift toward a more reliable and resilient supply chain is likely to affect the long-term energy and environmental prospects of EV batteries.

An effective estimate of the long-term impacts of rebuilding a more secure and resilient EV battery supply base amid the highly uncertain and dynamic EV market expansion and battery technology evolution pathways could yield policy implications of the potential trade-offs between the energy consumption and environmental impacts of LIBs. Previous studies have indicated that to achieve sectoral climate mitigation targets, current policies in the United States would require the electrification of over 90% of on-road light-duty vehicles, which could exacerbate material concerns due to uneven global resource distribution, geopolitical issues, and supply disruptions (*4*). Existing studies of EV battery material supply chains have attempted to examine the regional variability in LIB value chain components and explore the extent to which battery chemistry and circular economy strategies could alleviate the potential cobalt supply risk (*11*, *12*, *18*). These studies found that advancements in battery cathode technology can substantially mitigate cobalt’s long-term supply risk but shift the burden to nickel demand. Nevertheless, the battery technology evolution and EV market expansion may be highly uncertain and dynamic. The resulting changes in battery material demand and supply and variations in potential environmental implications of the supply chain restructuring are overlooked in previous analyses on the environmental sustainability of the EV battery supply chain (*18*–*20*). Furthermore, the geographical and logistical complexities of the entire EV battery value chain are not sufficiently captured, and the long-term implications of different levels of geopolitical risks remain unexplored.

In this study, we examine the long-term energy and sustainability prospects of the current trend toward bolstering a secure and resilient midstream and downstream supply chain of EV batteries under different trajectories of technology advancement and EV adoption. The upstream valuable metal mining and other battery materials are unchanged as they are mostly geographically concentrated in several resource-rich countries (*21*). Our analysis specifically considers the EV fleet in the United States, the largest LIB manufacturer with the most production capacity planned or under construction among the western countries (*16*, *22*). We present a comprehensive and detailed energy and environmental analysis on reshoring and ally-shoring current U.S. automotive LIB manufacturing by connecting the temporal and spatial variation in sourcing refined valuable metals, components, cells, and packs with the LIBs in major EV models of the U.S. EV fleet. To better investigate the long-term effects of the current trend to revitalize the domestic industrial base for EV batteries, we make a comprehensive comparison between the prepandemic case and future restructuring scenarios. From an energy and sustainability standpoint, we first illustrate the prepandemic reality of EV battery manufacturing by linking current spatial variation in sourcing the refined valuable metals, components, cells, and packs with the battery chemistry and capacity variation for the prepandemic U.S. EV fleet. We then address the long-term prospects of fostering a more resilient industrial base and assuring material supplies for EV battery technology as usual by 2050. The scenarios presented here incorporate projections of the power grid by 2050 and reflect varying levels of the future horizontal and vertical integration in the EV battery value chain to tackle its vulnerabilities. The U.S. manufacturing scenario fosters a domestic industrial base for the entire midstream and downstream LIB manufacturing. The ally-shoring scenario facilitates collaboration with U.S. allies and reallocates the U.S. imports of refined valuable metals, LIB components, cells, and packs from China to Japan, South Korea, the European Union (E.U.), and the United States. Last, we decipher the impacts of low-cobalt and no-cobalt battery technology penetration and EV market expansion on the environmental performances of supply chain restructuring from 2025 to 2050 compared to a business-as-usual (BAU) supply chain configuration. Key findings and policy implications that tackle environmental issues are summarized in the following sections.

## RESULTS

### Key findings

#### Battery technology as usual

  • Bringing back automotive LIB manufacturing to the United States will reduce ecotoxicity, eutrophication, and fine particulate matter formation by over 30% from the secondary nickel supply. However, it will not reduce ozone formation, ozone depletion, and ionizing radiation due to increased overseas transportation distance and intensive use of nuclear power.

• The ally-shoring strategy, designed to restructure a resilient EV battery value chain, reduces environmental impacts by 3 to 15% for most impact categories. However, it may lead to worse environmental performances in terrestrial acidification and fine particulate matter formation, unless nickel imports from Russia are reduced by at least 40%. Expanding allied capability for LIB manufacturing will not achieve more than 7% reductions in water- and terrestrial-related impact categories.

#### Penetration of new battery technologies

  • Increasing the shares of onshoring and production by allies for the battery supply chain of the future U.S. EV fleet can achieve up to 27% reduction in the carbon footprint relative to the BAU supply chain configuration but is not comparable to the up to 54% reduction from switching the battery technologies to less carbon-intensive blade lithium iron phosphate (LFP).

• The benefits of carbon emission reduction through supply chain restructuring are heavily dependent on the market penetration of battery cathode technology. In contrast to other battery chemistries, blade LFP reduces less carbon footprint with increasing degrees of reshoring and ally-shoring due to its inherent higher material efficiency from the cell-to-pack technology.

#### Policy implications

  • Concentrating LIB components, cell, and pack production in the demand zone would be beneficial to reducing LIB’s embodied emissions and avoiding carbon leakage.

• Adopting more secondary aluminum and nickel and importing nickel from nickel-rich ores can reduce the carbon footprint by up to 26% for the prepandemic U.S. EV fleet and by up to 37% when reshoring or ally-shoring the U.S. automotive LIB manufacturing.

• Increasing the shares of renewable energy in the power grid and reducing the shares of hard coal- and lignite-fired electricity would help improve the environmental sustainability of LIBs for the U.S. EV market.

• EV batteries with higher material efficiency and less dependency on carbon-intensive materials are less vulnerable to supply chain restructuring in terms of carbon footprint.

• To maximize climate change mitigation benefits, it is crucial to carefully integrate supply chain restructuring and battery technology promotion policies. This integration can yield up to a 54% benefit. However, the advantages of reshoring and ally-shoring vary substantially depending on battery technology projections, despite implementing promotion policies.

### Energy and sustainability of EV battery supply chain for the 2019 baseline case

**Fig. 1.**
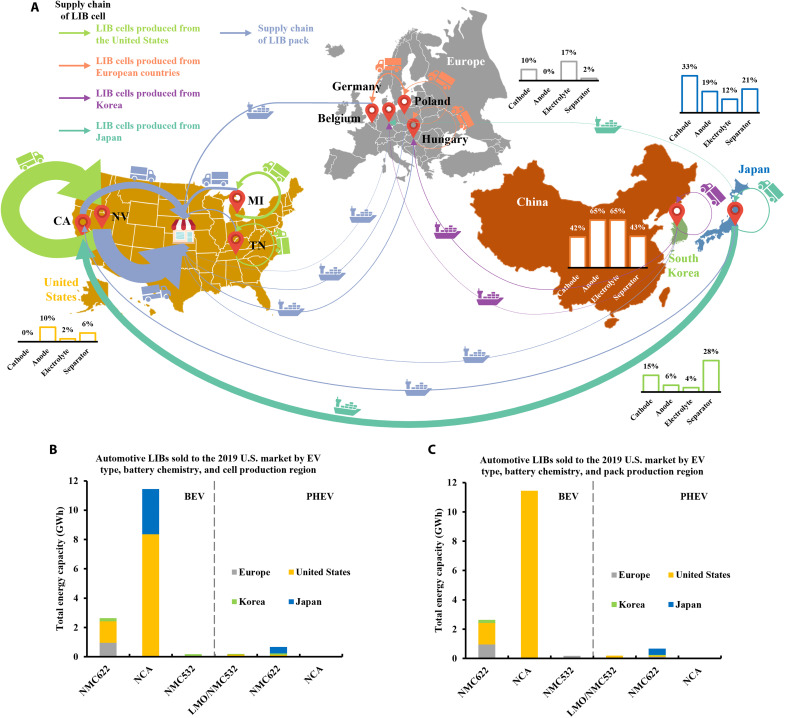
Midstream and downstream LIB manufacturing in the prepandemic U.S. EV market. (**A**) Flows of LIB components, cells, and packs for the prepandemic U.S. EV market. The column charts present the production capacities of LIB components by country on a percentage basis. Arrows represent the direction of flow, and the flow weights represent the amounts of cells and packs transported. Two transportation modes, i.e., by sea and truck, are illustrated for each flow. (**B**) Breakdowns of LIB sales by EV type, battery chemistry, and cell production region. (**C**) Breakdowns of LIB sales by EV type, battery chemistry, and pack production region. CA, California; NV, Nevada; MI, Michigan; TN, Tennessee.

To capture the long-term changes in the energy consumption and sustainability of the trend toward building a more robust and secure industrial base for EV batteries, we first present a detailed analysis for the U.S. case by linking current spatial variation in sourcing the refined valuable metals, components, cells, and packs with the LIBs in major vehicle models of the prepandemic U.S. EV fleet. The 2019 EV sales are used for the U.S. EV fleet case as it is the most updated prepandemic data. This section illustrates the energy and environmental performances of the EV battery supplies for the U.S. case, along with their influential factors. Our scope focuses on raw material acquisition, production of LIB components, cells, and packs, and transportation across automotive LIB manufacturing. First, we integrate real-world EV sales specifications with a bottom-up LIB design model to obtain the life cycle inventory of automotive LIBs. In addition, we construct a complex life cycle inventory for the distributed mining and refining of valuable metals and the production of raw materials, components, cells, and packs for these LIBs. Second, we evaluate the carbon footprint, cumulative energy demand (CED), and 17 ReCiPe midpoint impact categories, each focusing on a specific environmental issue for the U.S. case. These midpoint indicators are comprehensive and frequently reported by previous environmental assessment studies on LIBs (*23*–*25*). We define 1–kilowatt-hour (kWh) energy capacity of LIB manufactured as the functional unit. To account for the geographic variation in battery material production, we modify existing inventory data in ecoinvent to obtain the region-specific full-spectrum life cycle impact assessment results for raw materials of LIBs. A recycled content approach is adopted to quantify the environmental impacts of metals with both primary and secondary sources (*18*, *26*). To be more specific, our system boundary includes extraction, beneficiation, and refining of primary metal and preparation, recycling, and refining of secondary metal. Open-loop recycling is considered for aluminum, copper, steel, nickel, and lithium, and closed-loop recycling is considered for cobalt, as most recycled cobalt is sourced from battery recycling (*27*). Figure 1A and tables S1 to S4 present the flows of valuable metals, LIB components, cells, and packs for the prepandemic U.S. EV market. Valuable metals, including lithium, cobalt, and nickel, are mined and refined by their top producers. LIB component production depends on the East Asia countries, the E.U., and the United States following their respective shares of production capacities. LIB cells and packs are produced in various regions by their corresponding EV models.

The carbon footprint and energy consumption [1.6–metric tons (MT) CO_2_ eq. and 22 PJ] of LIB manufacturing for the U.S. EV fleet are equal to around 2 hours of the United States’ total greenhouse gas emissions and primary energy consumption in 2019 (*28*, *29*). The average carbon footprint and CED per kWh LIB are 103 kg of CO_2_ eq. and 1422 MJ eq. for the U.S. EV fleet. Although plug-in hybrid EVs (PHEVs) make up a quarter of EV models sold, it accounts for only 6% of the annual EV sales in terms of energy capacity. This is because PHEVs have lower energy capacities, ranging from 6 to 18 kWh compared to 18 to 100 kWh for battery EVs (BEVs). This finding suggests that the environmental performance of BEV LIBs primarily determines the carbon footprint and CED of the U.S. EV fleet. On the other hand, the mass of all PHEV LIBs accounts for around one-fifth of the total EV LIB mass due to low-energy density (i.e., the ratio of the rated capacity and battery weight). As a result, the carbon footprint and CED of PHEV LIBs (164 kg CO_2_ eq. and 2329 MJ eq.) are over 60% higher than BEV LIBs (85 kg CO_2_ eq. and 1319 MJ eq.).

Battery chemistry has a considerable impact on the environmental sustainability of LIB manufacturing primarily due to the inherent differences in energy density. The battery chemistries of the U.S. EV fleet include lithium nickel manganese cobalt oxide (NMC) 622, lithium nickel cobalt aluminum oxide (NCA), and NMC532 for BEVs and lithium manganese oxide (LMO)/NMC532, NMC622, and NCA for PHEVs, as shown in Fig. 1, B and C. Battery pack energy density is determined by its energy capacity and battery chemistry. LIB packs with high nickel content weigh less, resulting in higher battery pack energy density. Since LIBs with higher battery pack energy density require less material and energy input, the per-kWh carbon footprint and CED of NMC622 LIBs are 13% lower than that of NMC532 LIBs for BEVs. When both BEVs and PHEVs are considered, the difference narrows, as NMC622 represents 76% of PHEV batteries. This finding suggests that the energy capacity also has an influential impact on the environmental performance of LIBs. Moreover, NCA LIBs have a slightly higher (<4%) carbon footprint and CED compared to NMC622 in BEVs due to the substitution of manganese and Li_2_CO_3_ by more carbon- and energy-intensive nickel and LiOH. NCA and NMC622 LIBs account for 73 and 23% of the carbon footprint and CED for the U.S. EV fleet, respectively.

**Fig. 2.**
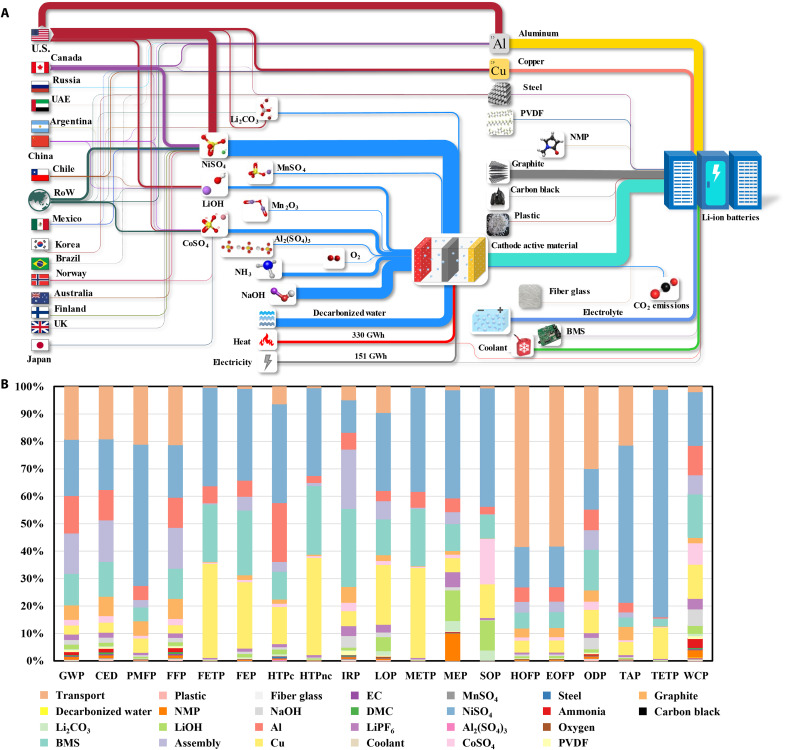
Material flow and environmental profile of LIBs for the prepandemic U.S. EV fleet. (**A**) Material flow of raw materials for the prepandemic U.S. EV fleet. The weight of each flow corresponds to its mass-based consumption rate. The exception is electricity and heat flows, whose weights are energy based. (**B**) Environmental profile of the automotive LIBs for the U.S. market.

In addition to the EV type and battery chemistry, other influential factors in the environmental impacts of LIB manufacturing include transportation, battery management systems, nickel, cobalt, aluminum, and copper. Figure 1A shows the production capacity of LIB components, cells, and packs in each region. Along the value chain from raw material extraction to LIB cell and pack assembly, LIB production becomes more concentrated toward the demand zone. Valuable metals are primarily mined in developing countries, while midstream refining is partly shifted toward developed countries. Three major Northeast Asian economies dominate the manufacturing capacity of all LIB components, including cathode, anode, electrolyte, and separator. For LIB cells and packs, the United States and Japan account for 90% of the energy capacity. In general, transportation distances of LIB cells and packs tend to decrease when LIB components are transported farther due to the concentration of LIB component production in Asia. Figure 2 reveals that transportation contributes to 58% of environmental impacts related to ozone formation, while battery management systems, NiSO_4_, aluminum, and copper dominate other impact categories. NiSO_4_ and CoSO_4_ explain 43 and 17% of the mineral resource scarcity because of cobalt’s high supply risk and nickel’s intensive use.

### Energy and environmental implications of supply chain restructuring

We examine the energy and environmental prospects of reducing dependency on EV battery imports compared to the U.S. EV fleet case, as summarized in the key findings. On the basis of the U.S. EV fleet case, we consider two future scenarios of securing reliable supply bases for the U.S. EV market by 2050 with battery technology as usual, when the temporal variation in the power grid is incorporated. In these two scenarios, the midstream and downstream EV battery value chains, including the production of LIB components, cells, and packs, are shifted according to different levels of geopolitical risks. The metal refining process is also affected. The upstream valuable metal mining and other battery materials are sourced globally following the U.S. EV fleet case.

• U.S. manufacturing scenario: The midstream and downstream LIB manufacturing is moved to the U.S. Specifically, LIB components, cells, and packs are produced in the U.S. Regarding raw materials, the refining of valuable metals and aluminum, copper, and steel production follow their U.S. supply chains (*21*) (table S5). The upstream mining and extraction of raw materials are unchanged.

• Ally-shoring scenario: In light of the United States’ plan to work with allies in securing its LIB supplies, this scenario eliminates the United States’ dependencies on importing refined valuable metals, LIB components, cells, and packs from China; The shares of production capacities for LIB components, cells, and packs in Japan, South Korea, EU, and the United States are reallocated as shown in table S6. The shares of China’s production capacity for refined valuable metals are reallocated to other trading partners of the United States (table S7), while the mining capacities remain unchanged.

**Fig. 3.**
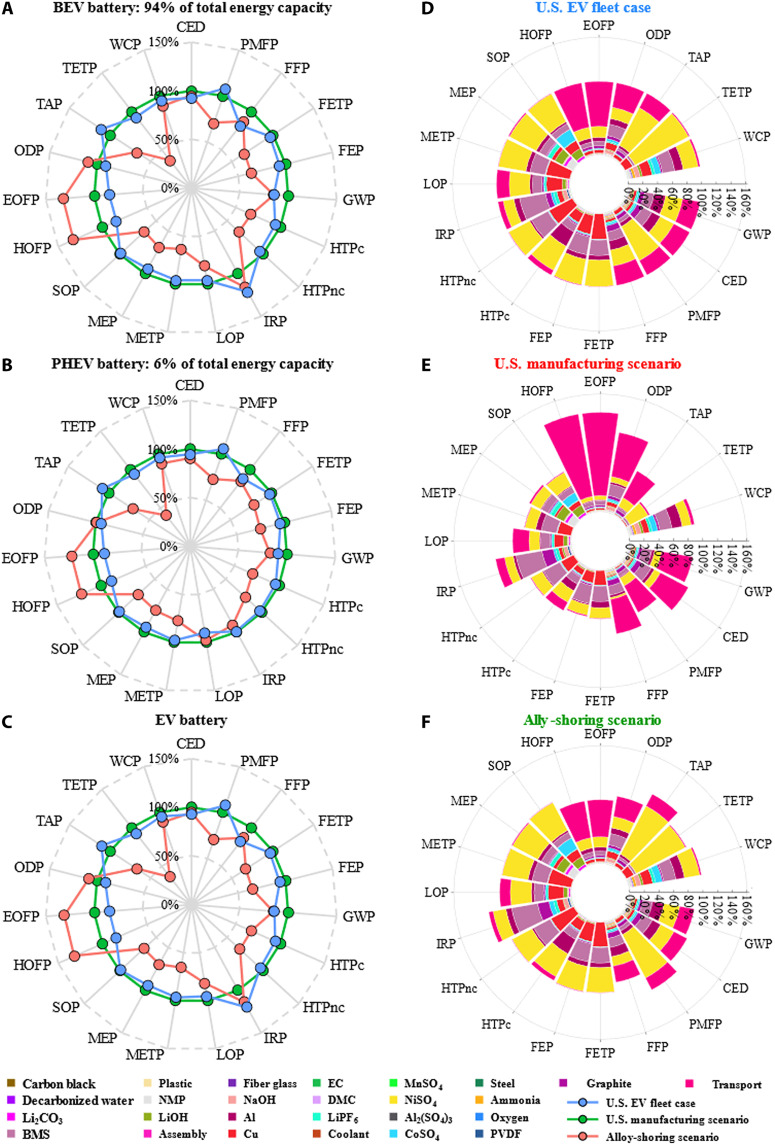
Comparison of full-spectral environmental impacts across the U.S. EV fleet case, U.S. manufacturing scenario, and ally-shoring scenario. (**A**) Comparison of life cycle environmental impacts of BEV LIBs across the three scenarios. The total energy capacity of BEV LIBs accounts for 94% of the U.S. EV fleet. (**B**) Comparison of life cycle environmental impacts of PHEV LIBs across the three scenarios. The total energy capacity of BEV LIBs accounts for 6% of the U.S. EV fleet. (**C**) Comparison of life cycle environmental impacts of EV LIBs across the three scenarios. (**D**) Breakdowns of life cycle environmental impacts for the U.S. EV fleet case. (**E**) Breakdowns of life cycle environmental impacts for the U.S. manufacturing scenario by 2050. (**F**) Breakdowns of life cycle environmental impacts for the ally-shoring scenario by 2050.

**Fig. 4.**
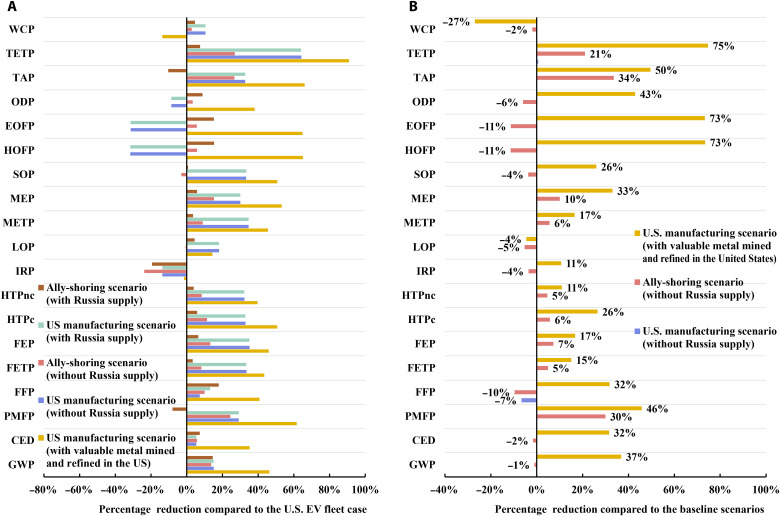
Analysis of interrupting nickel supply from Russia and domestic sourcing of critical battery materials. (**A**) Percentage reduction in full-spectrum environmental impacts compared to the U.S. EV fleet case. It is worth mentioning that these results serve as an upper bound of the energy and environmental impacts in terms of the variation in the assessed parameter. (**B**) Percentage reduction in full-spectrum environmental impacts compared to the baseline results.

The U.S. manufacturing scenario demonstrates reduced environmental impacts across all impact categories but ozone formation, ozone depletion, and ionizing radiation, compared to the U.S. EV fleet case and the ally-shoring scenario, as depicted in Fig. 3. Ozone formation increases by over 30% for the U.S. manufacturing scenario due to the additional nitrogen oxide emissions from longer overseas transportation distances by ferry. The inferior environmental performances of the U.S. manufacturing and ally-shoring scenarios for ionizing radiation can be largely attributed to the critical differences in energy sources of electricity used to produce LIB components, cells, and packs. In particular, for the U.S. EV fleet case, the majority of LIB cells and packs are produced in regions without a strong penetration of nuclear power, including Japan and the Western Electricity Coordinating Council region of the United States (*30*, *31*). The U.S. manufacturing scenario reduces terrestrial acidification and fine particulate matter formation by around 30%, while the ally-shoring scenario shows worse environmental performances than the U.S. EV fleet case. This outcome is due to the high-nickel LIB production dominating the U.S. EV market, which consumes a great amount of nickel, and the U.S. manufacturing scenario does not import nickel from the Norilsk Nickel plant in Russia, where SO_2_ emissions are uncontrolled (*32*). As the share of nickel imported from Russia increases due to the reallocation of the nickel supply for the U.S. EV fleet, terrestrial acidification and fine particulate matter formation increase by around 10%. However, the inferior environmental performance of the ally-shoring scenario can be reversed if at least 40% less nickel is imported from Russia. Considering the recent escalating geopolitical tensions between Russia and Ukraine and international sanctions imposed on Russia, we analyze energy and environmental implications for the two scenarios without nickel supply from Russia. The result suggests that terrestrial acidification, terrestrial ecotoxicity, and fine particulate matter formation can be reduced by up to 27% compared to the U.S. EV fleet case, as illustrated in Fig. 4A. The United States’ recent Defense Production Act aims to boost domestic production of key critical minerals and materials, including lithium, nickel, cobalt, and graphite. Our analysis indicates that sourcing key critical minerals domestically can further improve the environmental impacts of the U.S. manufacturing scenario by 11 to 75% in most impact categories. However, as geothermal brine may become the main lithium source in the United States, water depletion increases substantially due to intensive water use for lithium recovery (*33*). Moreover, the increasing truck transportation from raising domestic critical mineral sourcing leads to up to 4% more land use for the U.S. manufacturing scenario.

The U.S. manufacturing scenario substantially reduces freshwater-, marine-, and terrestrial-related ecotoxicity and eutrophication by over 30% compared to the U.S. EV fleet case, with a notable 64% reduction in terrestrial ecotoxicity. This improvement is mainly because 51% of nickel consumed in the United States is recovered from scrap, while less than 1% of the global nickel supply is from secondary production (*21*, *34*). The treatment of sulfidic tailing and other nickel mine operations cause the emissions of copper, nickel, zinc, and phosphate in the air and groundwater, causing severe harm to ecosystems and freshwater eutrophication. In contrast to the environmental benefits of nickel’s increasing secondary supply, more recycled aluminum could increase freshwater and marine ecotoxicity and water consumption. Preparing aluminum scrap for melting and recycling involves treating the by-product of scrap copper, which leads to emissions of copper ions to groundwater (*35*). Since the United States has the highest aluminum recycling rate in the world, the emissions of copper ions lead to higher freshwater and marine ecotoxicity in the U.S. domestic manufacturing scenario (*36*). On the other hand, primary aluminum production in the United States predominantly relies on hydroelectricity (*37*), causing the U.S. manufacturing scenario to consume more water than the ally-shoring scenario and the U.S. EV fleet case. Moreover, the ally-shoring scenario fails to achieve more than 7% reductions in water- and terrestrial-related impact categories. The critical differences in the share of lignite-generated electricity in the power grid for producing LIB components can mainly explain the inferior freshwater eutrophication of the ally-shoring scenario. Lignite has the lowest heating value, and its mining produces a large overburden, leading to excess phosphate emissions to groundwater (*38*). Although China, the largest producer of LIB components, relies heavily on coal-fired electricity, its share of lignite-generated electricity is minimal (*31*, *39*). Instead, higher percentages of lignite-generated electricity can be found in the power grid of other countries, including Poland, Hungary, Germany, South Korea, and the United States (*31*, *39*). The ally-shoring scenario results in more intensive water consumption than the U.S. manufacturing scenario, primarily due to the high share of water-intensive energy sources in the power grid that support CoSO_4_ and LIB production.

The carbon footprint, CED, and fossil resource scarcity are reduced by less than 15% for the U.S. manufacturing scenario and up to 18% for the ally-shoring scenario, compared to the U.S. EV fleet case. The reduction can be substantially enlarged for the U.S. manufacturing scenario if transportation is excluded, as shown in fig. S1. This difference is primarily due to variations in the aluminum recycling rate, emissions related to the energy consumption of primary aluminum production, and geographic variation in the electricity used to power the aluminum industry. Specifically, a global secondary aluminum supply rate of 34% is adopted for the U.S. EV fleet case and ally-shoring scenario and for the aluminum imports in the U.S. manufacturing scenario (*36*, *40*), while 76% of secondary aluminum production is considered for the domestic production in the United States (*41*). Primary aluminum production is an energy-intensive process. China is responsible for over half of the global primary aluminum production (*42*) and is among the best-performing regions in the world for the energy intensity of primary aluminum production (*37*). However, coal-fired electricity supplies around 90% of China’s primary aluminum production (*37*), resulting in substantially more acute greenhouse gas emissions, fossil depletion, and ozone depletion. Similarly, as the shares of coal- and oil-fired electricity in the power grid of China (68%), Japan (37%), South Korea (45%), Poland (74%), and Germany (34%), are much higher than that of the United States (24%) (*31*), the environmental impacts of the U.S. EV fleet case in carbon footprint and fossil resource scarcity are higher than that of the U.S. manufacturing and ally-shoring scenarios. Other factors contributing to the reduction benefits of the U.S. manufacturing and ally-shoring scenarios in carbon footprint, fossil resource scarcity, and ozone formation include the geographic variation in the ore grades of nickel and power grid. Notably, the U.S. manufacturing scenario can achieve reduction benefits in ozone formation only when transportation is excluded. Nickel ore grades vary across ore type and geographic location of ore deposit (*43*). Moreover, ore grades decline as mining continues, which results in substantially increasing unit intensity of environmental impacts (*43*). According to an extensive study on global trends in nickel mining (*43*), Canada achieved the best average ore grade among the countries with operating nickel mines in 2008. Although Canada accounts for 7% of the global nickel mine production for the U.S. EV fleet case, 20% of nickel consumed for the U.S. manufacturing scenario is imported from Canada. Therefore, the U.S. manufacturing scenario achieves reduction benefits in carbon footprint, fossil resource scarcity, and ozone formation. In terms of the geographic variation in electricity consumption, the environmental impact reduction benefits of the U.S. manufacturing scenario result from the stronger penetration of renewables in the power grid of the U.S. relative to other regions involved in the production of LIB components, cells, and packs (fig. S3) (*30*, *31*). Sensitivity analysis on the temporal variation in the power grid shows that the reduction benefits of restructuring value chain can be improved by up to 15% in carbon footprint and by up to 7% in CED for the U.S. manufacturing scenario and the ally-shoring scenario from 2019 to 2050 (fig. S13). As the EV battery manufacturing can also be relocated domestically, we conduct a sensitivity analysis on the temporal and spatial variation in the U.S. power grid for the U.S. manufacturing scenario, as shown in fig. S14. The result suggests that relocating the midstream and downstream automotive LIB value chain in the Northeast Power Coordinating Council and Western Electricity Coordinating Council region of the United States are the least carbon and energy intensive by 2050, respectively. On the contrary, the power grid in the Reliability First Corporation region of the United States is the most carbon and energy intensive in 2050.

### EV market expansion and battery technology penetration prospects

In the last section, we examine the environmental impacts of reshoring and ally-shoring the midstream and downstream EV battery supply chain with battery technology as usual. However, technological advances in battery chemistries, concerns over the supply risks, high extraction costs, and escalating prices of valuable metal may force the market to move beyond the current LIB technologies with ground-breaking low-cobalt or no-cobalt alternatives. In addition, carbon emission pricing could alter the market penetration of different types of EVs over time by influencing the production decisions of original equipment manufacturers and the purchase behaviors of customers. Therefore, the magnitude of environmental impacts of the EV battery could change notably with varying battery technology evolution and carbon emission pricing strategies, which are investigated in this study. We consider the BAU supply chain scenario and previously defined U.S. manufacturing and ally-shoring scenarios under future EV market penetration and battery technology projections from 2025 to 2050 based on previous studies, as illustrated in Table 1. Our approach adopts EV market expansion projections from a comprehensive climate change mitigation analysis. This analysis involves iterations between a U.S.-specific integrated energy model with a detailed technological representation of the U.S. passenger vehicle sector and its upstream sectors and a comprehensive vehicle life cycle assessment model to identify optimal decarbonization pathways under two carbon pricing policies (*2*).

**Table 1.** Overview of EV market penetration and battery technology evolution scenarios (*2*, *12*).

Scenarios	EV penetration	Battery technology evolution	Battery technology evolution description
**F-BT1**	EV penetration when fully pricing emissions (full pricing)	BT1	Current battery technologies are gradually replaced by state-of-the-art low-cobalt battery chemistries, such as NMC811 and NCA, until 2050.
**DE-BT1**	EV penetration when only pricing direct emissions (direct emission pricing only)		
**F-BT2**	Full pricing	BT2	Battery technologies are expected to shift toward more advanced low-cobalt battery chemistries, such as NMC955 and second-generation NCA (NCA-II), and reach 100% by 2050.
**DE-BT2**	Direct emission pricing only		
**F-BT3**	Full pricing	BT3	Current battery chemistries will be completely substituted by blade batteries with cobalt-free LFP technology by 2050.
**DE-BT3**	Direct emission pricing only		
**F-BT4**	Full pricing	BT4	Current battery technologies will be gradually replaced by state-of-the-art low-cobalt battery chemistries, such as NMC811 and NCA, until 2030. From 2030 to 2050, next-generation cobalt-free battery technologies, including Li-S and Li-air, are assumed to promptly penetrate the EV battery market until a complete substitution by 2050.
**DE-BT4**	Direct emission pricing only		

**Fig. 5.**
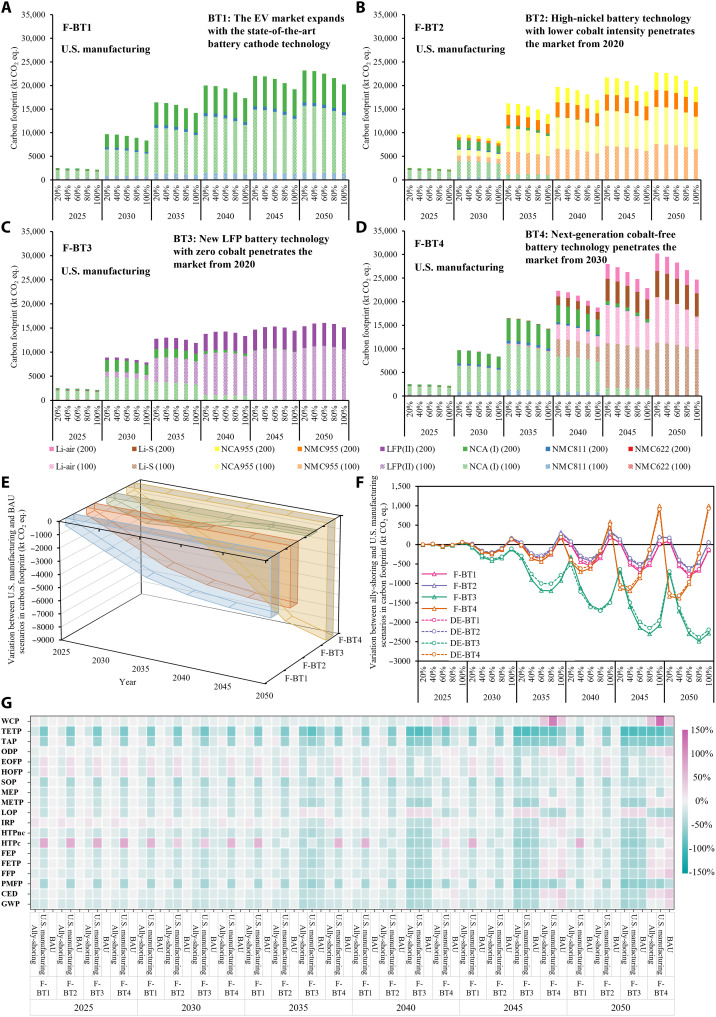
Comparison of environmental impacts across the U.S. EV fleet case, U.S. manufacturing scenario, and ally-shoring scenario under different EV market penetration and battery technology projections. (**A**) Comparison of carbon footprint for the U.S. manufacturing scenario under BT1 and EV market penetration when the full life cycle carbon emissions are priced (full-pricing scenario) from 2025 to 2050. (**B**) Comparison of carbon footprint for the U.S. manufacturing scenario under BT2 and full-pricing EV market penetration from 2025 to 2050. (**C**) Comparison of carbon footprint for the U.S. manufacturing scenario under BT3 and full-pricing EV market penetration from 2025 to 2050. (**D**) Comparison of carbon footprint for the U.S. manufacturing scenario under BT4 and full-pricing EV market penetration from 2025 to 2050. (**E**) Comparison of variations in the carbon footprint between the U.S. manufacturing and BAU scenarios for all battery technology projection scenarios from 2025 to 2050. (**F**) Comparison of variations in the per-kWh carbon footprint between the ally-shoring and U.S. manufacturing scenarios for all EV market penetration and battery technology evolution scenarios from 2025 to 2050. (**G**) Comparison of full-spectrum environmental impacts between the BAU, U.S. manufacturing, and ally-shoring scenarios for all EV market penetration and battery technology evolution scenarios from 2025 to 2050.

Increasing the degrees of production onshore or at the allies for the battery supply chain of future U.S. EV fleet curtain the carbon footprint by up to 27% through the adoption of next-generation cobalt-free battery technologies (BT4), such as lithium-sulfur (Li-S) and lithium-air (Li-air), than the BAU supply chain configuration but is not comparable to the up to 54% reduction resulting from switching to less carbon-intensive blade batteries with the cobalt-free LFP technologies, as shown in Fig. 5, A to D, and figs. S4 and S5. This difference is primarily due to the material- and energy-intensive cathode active material graphene-sulfur composite and electrolyte for the Li-S batteries and lithium anode active material and the additional aluminum oxygen tank for the Li-air batteries (*44*, *45*), which remain room for improvement. The carbon emission reduction benefits of supply chain reshoring and ally-shoring rely highly on the battery chemistry evolution trend, as presented in Fig. 5E. Specifically, with increasing degrees of reshoring and ally-shoring, blade LFP yields lesser reductions in the carbon footprint due to higher material efficiency by virtue of cell-to-pack technology, compared to other battery chemistries. Therefore, EV batteries with higher material efficiency and reduced reliance on carbon-intensive materials are less vulnerable to supply chain restructuring in terms of carbon footprint. Gradually substituting current battery technologies with the blade LFP (BT3) reduces per-kWh environmental impacts for all impact categories except the land occupation from 2035, for both BAU and supply chain restructuring scenarios. When the next-generation cobalt-free Li-S and Li-air technologies dominate the EV battery market, the unit environmental performances deteriorate, especially in CED, fossil depletion, freshwater ecotoxicity, ionizing radiation, marine ecotoxicity, ozone depletion, and water consumption due to less efficient material and energy use.

The benefits of reshoring and ally-shoring become more pronounced along the projection years due to increasing EV market penetration (Fig. 5F). Fully regulating vehicles’ life cycle carbon emissions could accelerate the penetration of BEVs but may also result in a higher share of 100-mile BEVs that bear more per-kWh carbon emissions than the 200-mile BEVs for their batteries. As a result of lower material efficiency for the 100-mile BEVs, the variation in carbon footprint between the ally-shoring and U.S. manufacturing scenarios becomes more prominent when the entire supply chain emissions are regulated. Ally-shoring outperforms reshoring for BT3 and for BT4 when supply chain restructuring is no more than 80%. The carbon footprint of the U.S. manufacturing and ally-shoring scenarios are close for BT1 and BT2, regardless of the EV market penetration level. These variations suggest that the supply chain restructuring policy should be carefully formulated to reduce the carbon footprint.

## DISCUSSION

EV manufacturing and trade play an important role in accelerating vehicle electrification and mitigating climate change. This study presents a novel analysis of the long-term implications of the current trend toward building a reliable and resilient midstream and downstream EV battery supply chain from the energy and environmental perspectives based on the U.S. EV fleet case under different trajectories of battery technology evolution and EV market expansion. The prepandemic U.S. case is considered since the United States has the most LIB production capacity that is operational, announced, or under construction among the western countries (*16*, *22*). Tesla/Panasonic, LG Chem, and SKI have announced their plans to implement 108 GWh of LIB cell production sites by 2025 (*46*). Moreover, Redwood Materials, a leading U.S. LIB recycler, plans to build a new plant in the United States to produce active cathode material and anode copper foils for 100 GWh of LIBs annually by 2025 (*47*). Our results estimate the carbon footprint and energy consumption as 1.6 MT and 22 PJ for the logistically complex cross-border supply chain of EV LIBs in the prepandemic U.S. market. The carbon emission reduction potential is 0.23-MT CO_2_ eq. by reshoring and ally-shoring United States’ automotive LIB manufacturing with battery technology as usual. As the EV market gradually expands to over 90% and advanced low-cobalt or cobalt-free battery technologies penetrate the market, the carbon emission reduction potential of LIB manufacturing can reach up to 8.9-MT CO_2_ eq. for the onshore production and 7.9-MT CO_2_ eq. for ally-shoring, relative to the BAU supply chain configuration. The carbon footprint mitigation benefits of supply chain restructuring for the U.S. EV fleet equate to 12 to 13.5 hours of the 2019 U.S. greenhouse gas emissions. However, manufacturing LIBs in the United States show worse environmental performances in ozone formation, ozone depletion, and ionizing radiation due to increasing overseas transportation distance and the intensive use of nuclear power. The conclusion of this study has important implications for other countries aiming to secure a reliable supply base for LIBs.

EV battery technology will continue to innovate battery chemistry, recycling, and capacity improvements. Therefore, the LIB value chain can be further decarbonized by adopting advanced cathode active materials with higher energy density, phasing out PHEVs, upgrading battery pack energy capacity, and optimizing battery designs for less material requirement. The carbon emission reduction benefits of supply chain restructuring are highly dependent on the market penetration of battery cathode technology. We find that the carbon emission reduction benefits of transition toward the blade LFP can be one time more than that of the supply chain restructuring. On the other hand, with increasing degrees of reshoring and ally-shoring, the LFP-dominant trend reduces the carbon footprint less substantially than the projected penetration of other battery technologies due to its inherently higher material efficiency from the cell-to-pack technology. EV batteries with higher material efficiency and less dependency on carbon-intensive materials are less vulnerable to supply chain restructuring in terms of carbon footprint. Moreover, there is considerable variation in the advantages of reshoring and ally-shoring due to varying battery technology projections. Adopting low-cobalt and cobalt-free cathodes can help mitigate the prospective shortage of cobalt, although not entirely eliminate it. However, it may also hinder the environmental benefits of closed-loop recycling of EV batteries (*12*, *23*). Thus, the supply chain restructuring and battery technology promotion policies should be carefully formulated to reduce the carbon footprint.

Our analysis yields several implications for restructuring the LIB supply chain. First, concentrated production of LIB components, cells, and packs in the demand zone would be beneficial to reducing LIB’s embodied emissions and avoiding carbon leakage. The second is to adopt more secondary aluminum and nickel. In figs. S15 to S17, our sensitivity analysis result shows that using 100% secondary nickel would reduce the carbon footprint by 26 to 37%, CED by 23 to 33%, and mineral resource scarcity by 27 to 43% for the U.S. EV fleet, suggesting a greater emission reduction benefit compared to adopting 100% recycled cobalt, aluminum, copper, or steel. Third, avoiding the import of nickel from ores with low nickel content and uncontrolled SO_2_ emissions would efficiently decarbonize and reduce terrestrial acidification of domestic and allied LIB production. Last, increasing the shares of renewable energy in the power grid while reducing the shares of hard coal- and lignite-fired electricity would help improve the environmental sustainability of EV LIBs. Sensitivity analysis of the temporal and spatial variation in the power grid shows that the climate change mitigation benefits of supply chain restructuring can be improved by up to 50% for U.S. manufacturing and up to 83% for the ally-shoring scenario from 2019 to 2050, as shown in figs. S13 and S14. With the projected electricity generation by energy source for power grids until 2050, the reduction benefits in CED can be improved from 2.9 to 5.4% for the U.S. manufacturing scenario and from 3.6 to 7.2% for the ally-shoring scenario. Because Russia is the world’s major fuel and mineral exporter, the recent Russia-Ukraine war may create additional geopolitical uncertainties to the supply risk faced by the global EV battery supply chain. Expanding domestic production of key critical minerals and materials can further improve the emission reduction benefits of the U.S. manufacturing scenario by 11 to 75% in most impact categories, albeit with intensified water use due to lithium recovery from geothermal brine. Technological advancements in cobalt and nickel extraction, such as deep-sea mining, hold the potential to mitigate the supply risk of valuable battery materials and reduce dependence on geographically concentrated land-based mining, which could cause contamination and damage to terrestrial environments and give rise to social issues such as the use of child labor (*48*, *49*). However, this study does not assess the viability of deep-sea mining due to uncertainties regarding its environmental and ecological effects, the potential impact on international relations, economic considerations, and technological challenges, as per the current state of knowledge (*49*, *50*).

Although reshoring and ally-shoring the EV battery supply chain for the U.S. EV fleet may benefit the environment, it may not be economically advantageous. Our results, detailed in Supplementary Text, demonstrate that high-performance LIB’s cost advantage is affected by variations in labor and material costs. The transportation cost also contributes minimally, by less than 2%, to LIB manufacturing cost. Because of high labor and material costs in the United States, disrupting all cross-border value chains can increase the manufacturing cost by 11%, while ally-shoring the EV LIBs manufacturing in the U.S. market increase the cost by 5%. Soaring energy prices can accelerate transportation electrification, increasing the pressure on critical battery material supply. However, the international sanctions against Russia and its export ban for certain products and raw materials may present greater supply risks and lead to surges in nickel prices. Our analysis reveals that the potential nickel supply interruption from Russia can reverse the inferior environmental performance of ally-shoring the EV battery supply chain in terrestrial acidification and fine particulate matter formation. Nevertheless, the resulting record-high nickel price can increase the manufacturing cost by 16 to 21%, creating challenges in the demand for vehicle electrification.

Implementing emission pricing, especially the indirect emissions from vehicle production, from the U.S. light-duty vehicle sectors would accelerate the technological change toward an EV-dominated future by 2050 (*2*). In Supplementary Text, our findings indicate that to achieve parity with import counterparts, a breakeven border carbon adjustment between the United States and the LIB exporting countries in Asia at $65 to $1051/t CO_2_ eq. for BEV LIBs and $110 to $1543/t CO_2_ eq. for PHEV LIBs can be imposed. The breakeven border carbon adjustments can be reduced when sourcing essential critical minerals and materials domestically from the United States in line with its recent Defense Production Act. To comply with World Trade Organization’s nondiscrimination principles, the same border carbon adjustment should be levied on domestic LIB products (*51*, *52*). High border carbon adjustments may lead to a faster phase-out of internal combustion engine vehicles and PHEVs. While imposing border carbon adjustments on LIBs may reduce carbon leakage and advance the domestic competitiveness of the automotive LIB industry, it may be perceived as a form of protectionism. It could threaten the climate target by stimulating demand for conventional internal combustion engine vehicles (*52*). U.S. and European Commission’s former experiences of imposing taxes on solar photovoltaic products suggest that these trade restriction measures could hinder solar deployment and consequently reduce the carbon emissions mitigation potential in the long term, although a recent study reveals the environmental benefits of reshoring silicon photovoltaics manufacturing (*53*–*55*).

## MATERIALS AND METHODS

Here, we introduce the methodology to assess the long-term effects of the trend toward building a more secure and resilient EV battery value chain from the energy and environmental perspectives. This analysis is conducted based on the LIB supply chains for the prepandemic U.S. EV fleet since the United States is the largest LIB producer in Western countries (*16*). Last, in the Supplementary Materials, we show the breakeven border carbon adjustment calculation to examine the potential to achieve climate parity between U.S. EV LIB products and their counterparts imported from E.U. and East Asia countries.

### Energy and environmental analysis of supply chain restructuring

We conduct a life cycle assessment to assess the energy and environmental performance of EV battery manufacturing for the U.S. EV fleet case in four steps, namely, goal and scope definition, LCI, life cycle impact assessment, and interpretation, following the ISO 14040 standard (*56*).

### Scope and system boundary

In this work, we aim to evaluate the “cradle-to-site” environmental impacts of LIB manufacturing and assess the potential of reducing environmental impacts for regional variations in the production of LIB components, cells, and packs. This study focuses on the stages of raw material acquisition, LIB component production, LIB cell production, LIB pack production, and transportation. Use and end-of-life phases are out of the scope of this study. The transportation stage encompasses the transportation of raw materials, components, cells, and packs of LIB. We define the functional unit as 1-kWh energy capacity of LIB manufactured. Besides the environmental impacts based on the functional unit, we report the net environmental impacts of the LIB supplies for EVs in the United States. We use the carbon footprint, CED, and 17 ReCiPe midpoint indicators from the hierarchist perspective to examine the burden across various environmental impact categories. These categories include fine particulate matter formation, fossil resource scarcity, freshwater ecotoxicity, freshwater eutrophication, human carcinogenic toxicity, human noncarcinogenic toxicity, ionizing radiation, land use, marine ecotoxicity, marine eutrophication, mineral resource scarcity, ozone formation (human health), ozone formation (terrestrial ecosystems), stratospheric ozone depletion, terrestrial acidification, terrestrial ecotoxicity, and water consumption.

### Life cycle inventory for the U.S. EV fleet case

The life cycle inventory phase complies with the energy and material inputs and outputs across all the life cycle stages of LIB manufacturing and distribution. We obtain the U.S. EV sales data by model from the U.S. Department of Energy (*57*). Specifically, 2019 U.S. EV sales data are the most updated and is used to represent the U.S. EV fleet case. EV is classified as BEV and PHEV. Tables S1 and S2 list the battery capacity, original equipment manufacturers, and production locations of LIB cells and packs based primarily on a recent report from Argonne National Laboratory and a recent study (*11*, *58*). The fuel economy (i.e., electricity consumption rate per kilometer) and an all-electric range of each EV model are extracted from the official U.S. government source for fuel economy information (*59*). Battery chemistries for different EV models are obtained from various sources, including literature, industrial report, and government report (*11*, *60*). According to the EV type, battery capacity, battery chemistry, and fuel economy, the material inventories of LIBs for different models of the U.S. EV fleet are extracted from the BatPac model and summarized in tables S8 to S10 (*61*). We estimate energy inventories from existing literature (*8*), considering dry room operation for cell assembly, cell charging, charge-retention testing, and the slurry pumping, coating, coiling, cutting, heating, drying, and calendaring for LIB components, including cathode, anode, and separator. Energy inventories of LIB manufacturing can be found in table S12. A total of 80% of the binder solvent is recovered because of its high price and environmental damage if emitted (table S13) (*62*). Since, production capacities of LIB components specific to EV models or U.S. EV fleet are not available, we use the best-available region-specific capacity data to represent LIB component production for the U.S. EV fleet case, as summarized in table S3 (*17*). Notably, while China is not a manufacturer of LIB cells and packs for the U.S. EV fleet, it is the largest producer of all LIB components. Similarly, we adopt the best-available country-level production capacity for mining and refining valuable metals, including lithium, cobalt, and nickel, from a recent government report, as shown in table S4 (*17*, *21*). The top producers of valuable metal mining are identified to include over 80% of global mining capacity (*21*). Since Canada and Russia produce substantially more nickel ores and concentrates than imports, we only consider domestic sources for nickel refining and nickel sulfate production in Canada and Russia (*21*, *63*). Likewise, domestic lithium sources of lithium carbonate and hydroxide production in Chile and Argentina are considered. For other top refining countries of valuable metals, ores and concentrates are sourced from the top mining countries according to their shares of mining capacities. For regions producing cathodes, sulfates of valuable metals are obtained from the top refining countries with shares proportional to their refining capacities. For metals produced and imported to the United States, primary and secondary sources are identified and summarized in table S5 (*21*). For other regions producing LIB cathodes, average global primary and secondary metal supply rates are considered for all metals (table S14) (*34*). As the majority of recycled cobalt is sourced from battery recycling, we consider cobalt recycling from NMC333 by the hydrometallurgical and pyrometallurgical process in major LIB recycling regions, including China, E.U., Canada, and South Korea, as summarized in table S15 (*27*). Transportation between different regions for raw materials, LIB components, cells, and packs is calculated and deposited in the GitHub repository.

### Life cycle inventory for the U.S. manufacturing scenario

On the basis of the current trend to assure material supply and revitalize the domestic industrial base in the United States, we consider two supply chain restructuring scenarios with different levels of geopolitical risks. The U.S. manufacturing scenario establishes the midstream and downstream manufacturing capacity of automotive LIBs in the United States. Specifically, LIB components, cells, and packs are produced in the United States. In terms of raw materials, the refining of valuable metals and production of aluminum, copper, and steel follow their U.S. supply chains (table S5) (*21*). However, the upstream mining and extraction of raw materials remain unchanged.

### Life cycle inventory for the ally-shoring scenario

In light of the United States’ plan to strengthen engagement with allies and to rebuild a broader domestic and allied industrial base, we consider the ally-shoring scenario that eliminates the United States’ dependencies on importing refined valuable metals, LIB components, cells, and packs from China. In this scenario, China’s shares of production capacities for refined valuable metals, LIB components, cells, and packs are reallocated to Japan, South Korea, the E.U., the United States, and other trading partners of the United States, proportionally to their respective production capacities, as shown in tables S6 and S7.

### Life cycle impact assessment

Life cycle inventory requires the production of raw materials in different regions. However, many region-specific impact assessment data are unavailable. Therefore, we modify the unit processes in ecoinvent to accommodate the need for region-specific impact assessment data, following ecoinvent’s extrapolation method for unit processes without region-specific data (*35*). In essence, this method extrapolates the material and energy inventory of an original dataset covering a specific region and replaces the linked activities with ones covering the region of interest where possible. Higher SOx emissions are considered for Russian-sourced nickel due to the uncontrolled SO_2_ emissions from the Norilsk Nickel plant reported in the previous literature (*32*). Characterization factors are mainly collected from ecoinvent with and without modifying the unit processes. However, the characterization factors of cathode active materials other than LMO are inaccessible. In addition, the characterization factor of CoSO_4_, as a critical raw material for producing the cathode active materials, is not accessible from the ecoinvent database either. Therefore, we construct region-specific production processes for CoSO_4_ and cathode active materials in the openLCA software and therein obtain their characterization factors. Hydrometallurgical and pyrometallurgical recycling processes for cobalt are constructed using inventories of LIB recycling from a recent study based on mass allocation to obtain the corresponding characterization factors, as shown in tables S16 and S17 (*23*, *64*). Detailed modifications of ecoinvent processes and lists of characterization factors are summarized in the GitHub repository.

## Supplementary Materials

### This PDF file includes:

Supplementary Text Figs. S1 to S21 Tables S1 to S28 References
