# Supplementary material for: Will reshoring manufacturing of advanced electric vehicle battery support renewable energy transition and climate targets?
Source: Sci Adv. 2023 Jun 14;9(24):eadg6740. doi: 10.1126/sciadv.adg6740 (PMC10266728; doi:10.1126/sciadv.adg6740)
Supplement: Supplementary file 1 — Supplementary Text Figs. S1 to S21 Tables S1 to S28 References [file sciadv.adg6740_sm.pdf]

Science Advances
AAAS

Supplementary Materials for

Will reshoring manufacturing of advanced electric vehicle battery support renewable energy transition and climate targets?

Apoorv Lal *et al.*

Corresponding author: Fengqi You, fengqi.you@cornell.edu

Sci. Adv. **9**, eadg6740 (2023)
DOI: 10.1126/sciadv.adg6740

This PDF file includes:

Supplementary Text
Figs. S1 to S21
Tables S1 to S28
References

Supplementary Text

Energy and environmental analysis of electric vehicle (EV) battery supply chain

Fig. S1 compares the full spectrum environmental impacts of the United States (US) EV fleet case, US manufacturing scenario, and ally-shoring scenario with transportation excluded. **Fig. 3** and **Fig. S1** show that all impact categories achieve more reduction when transportation is not considered for the US manufacturing scenario, while no substantial change in the environmental impacts of the ally-shoring scenario can be observed. By comparing the full spectrum of environmental impacts across the US EV fleet case, US manufacturing scenario, and ally-shoring scenario by different battery chemistry, as shown in **Fig. S2**, we found that the reduction benefits in most impact categories are rather insusceptible to the change in battery chemistry. **Fig. S2** also suggests that Plug-in hybrid electric vehicle (PHEV) batteries are less detrimental in terms of ionizing radiation and ozone-related environmental issues. **Fig. S3** presents the variation in the electricity mix of different regions where LIB components, cells, and packs are produced. Notably, coal-fired electricity dominates the electricity generation in Poland and China; gas-fired power generation in Japan, Korea, and the US are the most influential; Belgium and Germany have a stronger penetration of renewables in the power grid. **Fig. S4** and **Fig. S5** compare the carbon footprint across the US EV fleet case, US manufacturing scenario, and ally-shoring scenario under different EV market penetration and battery technology projections. **Fig. S6** represents the projection of battery technology penetration under BT1, BT2, BT3, and BT4 scenarios. **Fig. S7** illustrate the EV market expansion under two carbon emission regulation strategies, one only prices direct tailpipe emission and the other prices full life-cycle carbon emissions.

Table S1–STable S2 enumerate the EV sales by the model in the US with the original equipment manufacturers (OEMs) for lithium ion battery (LIB) cells and packs, production location of LIB cells, packs, and EV, battery capacity, electric range, energy efficiency, and battery chemistry. **Table S8–STable** S10 show the material inventory of LIBs for the EV batteries listed in **Table S1–STable** S2. **Table S11** lists the material inventory of LIBs for projection of battery technology penetration. The energy inventory of LIB manufacturing is available in **Table S12**. Material and energy inventory to recover the binder solvent N-methyl-2-pyrrolidone (NMP) is listed in **Table S13**. **Table S3–STable** S4 present the production capacities of LIB components and valuable metal mining and refining in their major production regions. **Table S6–STable** S7 reallocate the production capacities of LIB components and valuable metal mining and refining for the ally-shoring scenario. The percentage of primary and secondary metals, as well as sources of primary copper and secondary aluminum, are available in **Table S5**, **STable** S14, and **STable** S18. **Table S15** estimates the percentage of hydrometallurgical and pyrometallurgical recycling by region according to the operational LIB recyclers (*27*). **Table S16–STable** S17 demonstrate

the material and energy inventory of cobalt recycling with hydrometallurgical and pyrometallurgical recycling of lithium nickel manganese cobalt oxide (NMC333).

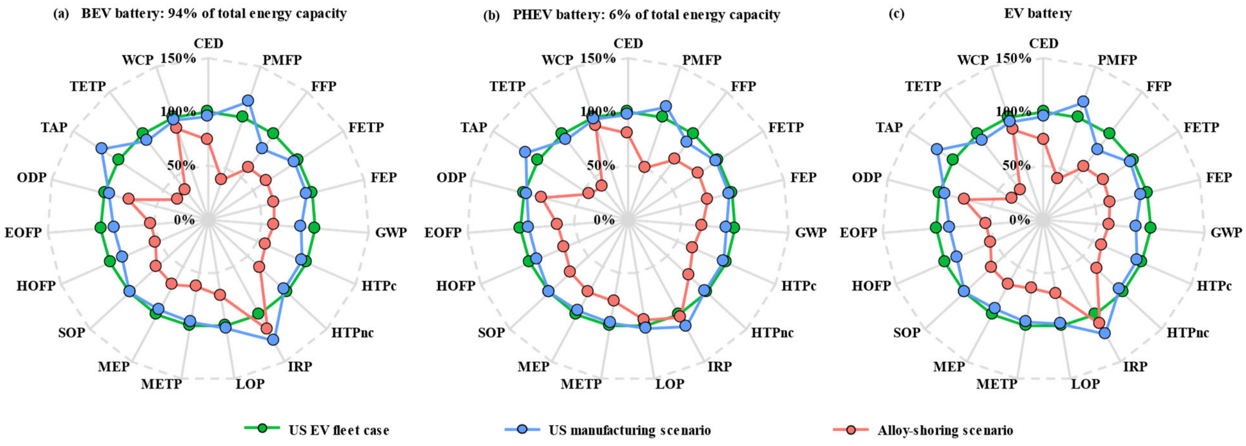

Fig. S1 Comparison of full-spectral environmental impacts across the US EV fleet case, US manufacturing scenario, and ally-shoring scenario, with transportation excluded. a, Comparison of life cycle environmental impacts of battery electric vehicle (BEV) LIBs across the three scenarios. The total energy capacity of BEV LIBs accounts for 94% of the 2019 US EV fleet. **b**, Comparison of life cycle environmental impacts of PHEV LIBs across the three scenarios. The total energy capacity of BEV LIBs accounts for 6% of the 2019 US EV fleet. **c**, Comparison of life cycle environmental impacts of EV LIBs across the three scenarios. Acronyms: global warming potential (GWP); cumulative energy demand (CED); fine particulate matter formation (PMFP); fossil resource scarcity (FFP); freshwater ecotoxicity (FETP); freshwater eutrophication (FEP); human carcinogenic toxicity (HTPc); human non-carcinogenic toxicity (HTPnc); ionizing radiation (IRP); land use (LOP); marine ecotoxicity (METP); marine eutrophication (MEP); mineral resource scarcity (SOP); ozone formation (human health) (HOFP); ozone formation (terrestrial ecosystems) (EOFP); stratospheric ozone depletion (ODP); terrestrial acidification (TAP); terrestrial ecotoxicity (TETP); water consumption (WCP).

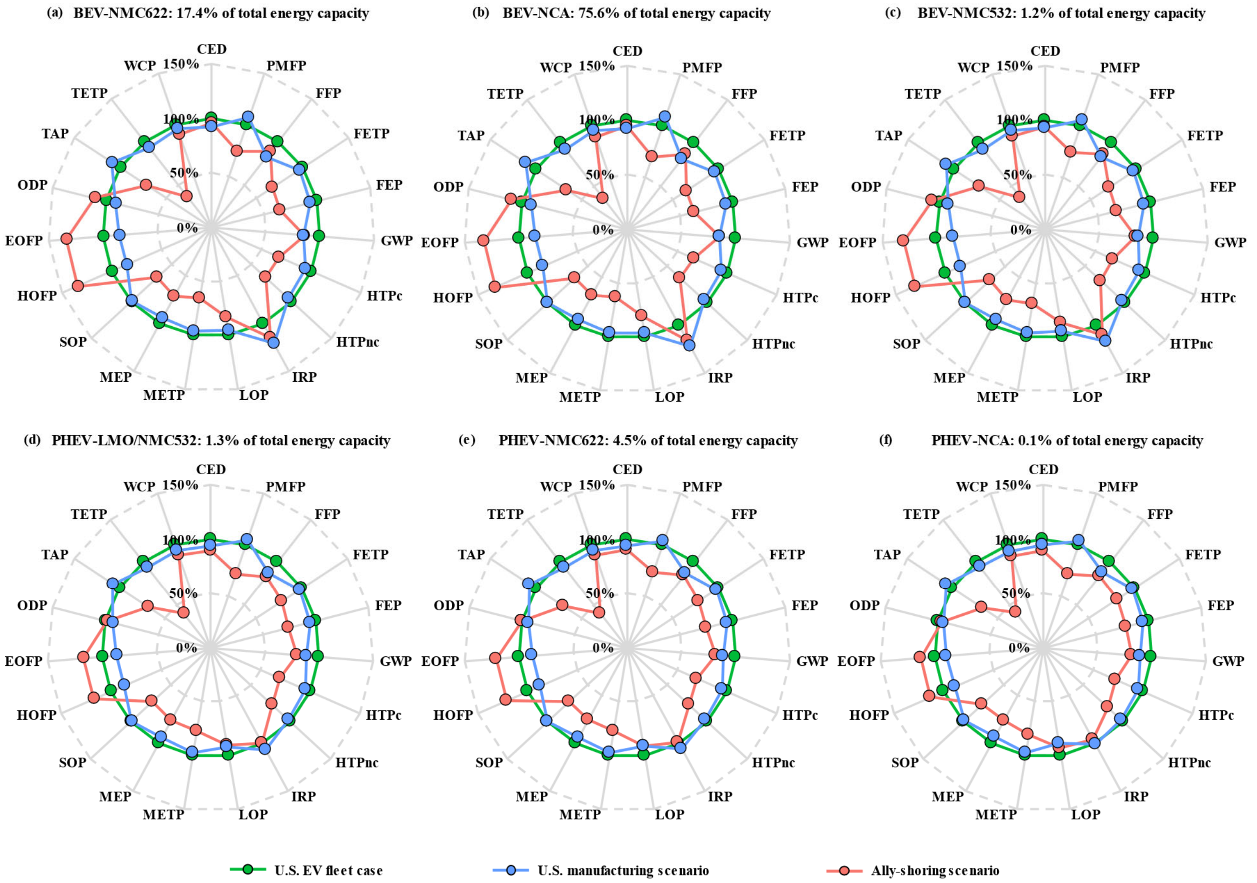

Fig. S2 Comparison of full-spectral environmental impacts across the US EV fleet case and US and ally-shoring scenarios by different battery chemistry. a, Comparison of full-spectral environmental impacts for NMC622 in BEV. **b**, Comparison of full-spectral environmental impacts for lithium nickel cobalt aluminum oxide (NCA) in BEV. **c**, Comparison of full-spectral environmental impacts for NMC532 in BEV. **d**, Comparison of full-spectral environmental impacts for lithium manganese oxide (LMO)/NMC532 in PHEV. **e**, Comparison of full-spectral environmental impacts for NMC622 in PHEV. **f**, Comparison of full-spectral environmental impacts for NCA in PHEV.

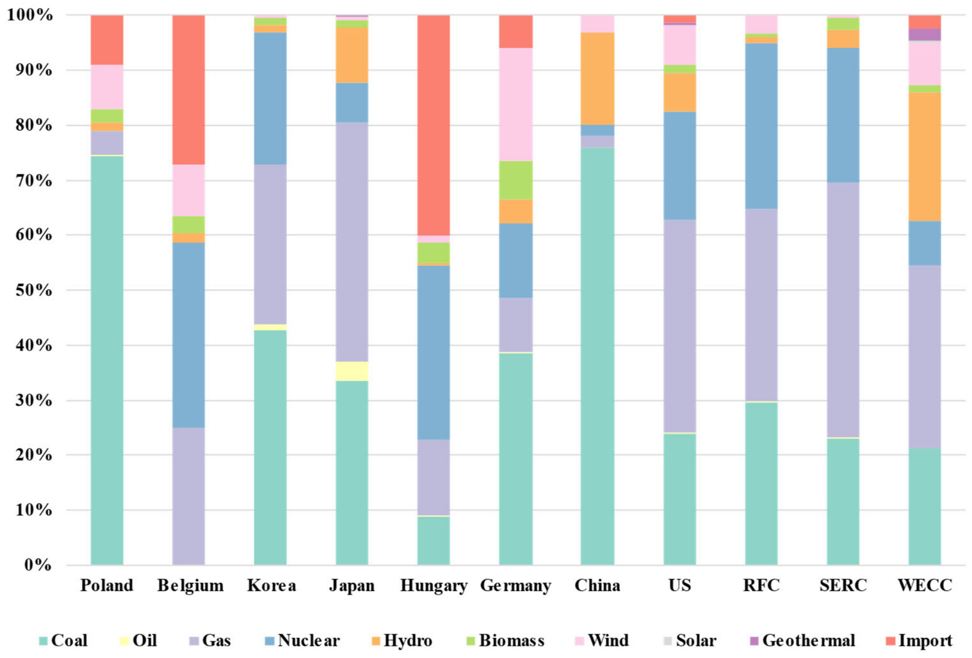

Fig. S3 Energy sources of power grids in the US, Poland, Belgium, Korea, Japan, Hungary, Germany, and China, where LIB components, cells, and packs are produced (*30, 35*).

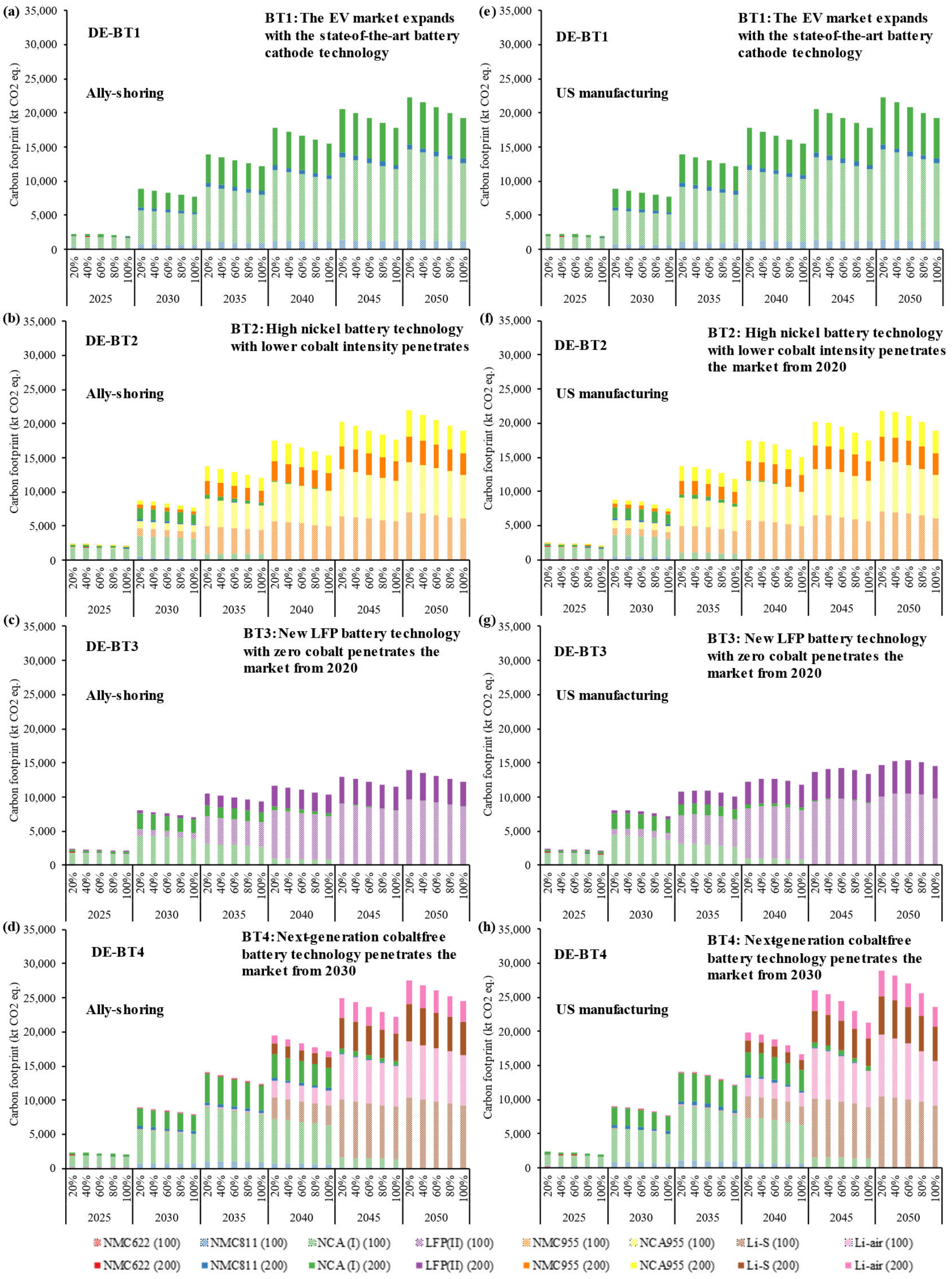

Fig. S4 Comparison of environmental impacts across the US EV fleet case, US manufacturing scenario, and ally-shoring scenario under different EV market penetration and battery technology projections. a-d, Comparison of carbon footprint for the ally-shoring scenario under BT1, BT2, BT3, and BT4, respectively, and EV market penetration when the tailpipe emissions are priced (direct-emission-pricing only scenario) from 2025 to 2050. **e-h**, Comparison of carbon

footprint for the US manufacturing scenario under BT1, BT2, BT3, and BT4, respectively, and direct-emission-pricing only EV market penetration from 2025 to 2050.

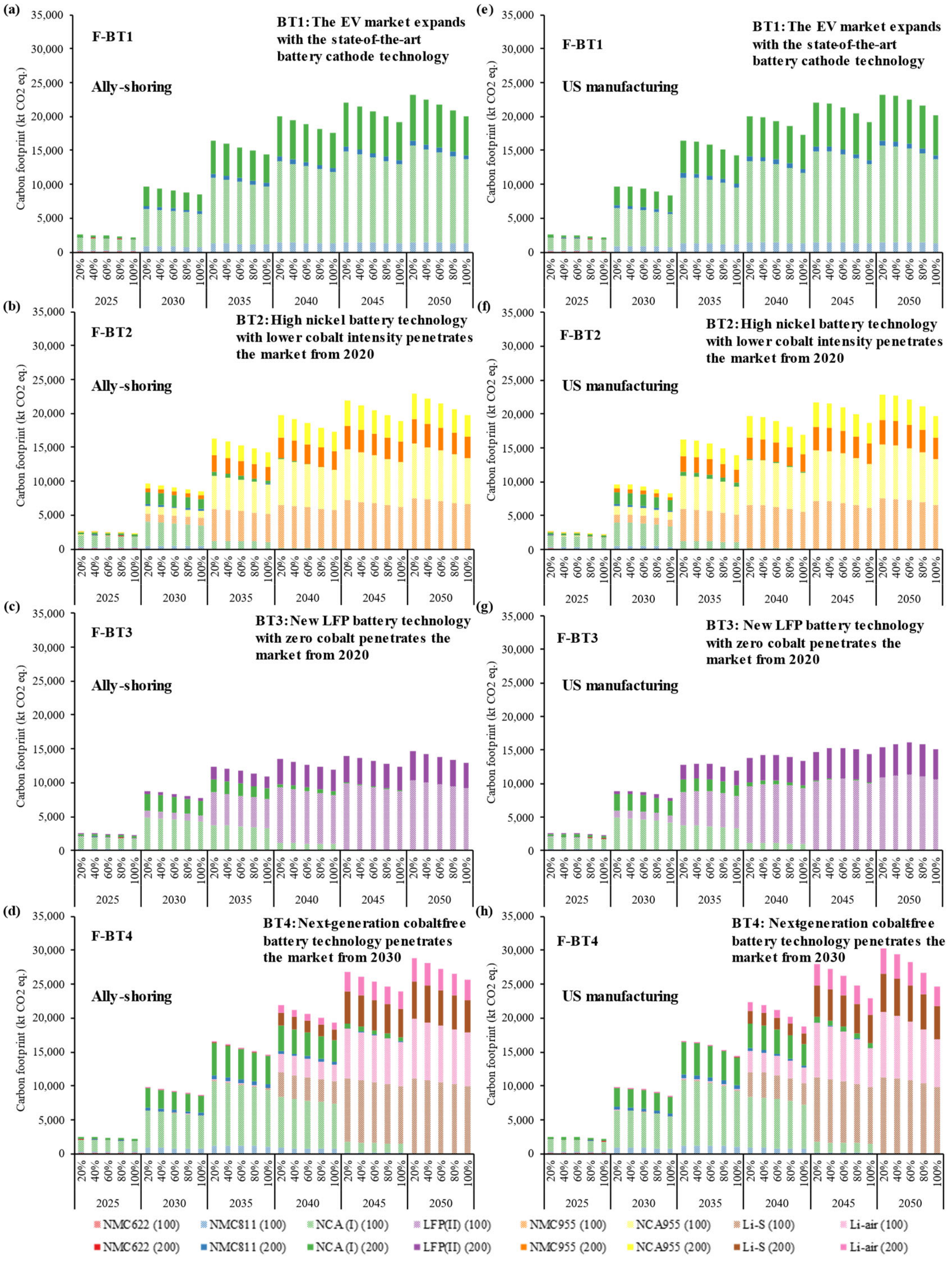

Fig. S5 Comparison of environmental impacts across the US EV fleet case, US manufacturing scenario, and ally-shoring scenario under different EV market penetration and battery technology projections. a-d, Comparison of carbon footprint for the ally-shoring scenario under BT1, BT2, BT3, and BT4, respectively, and EV market penetration when the full life cycle carbon

emissions are priced (full-pricing scenario) from 2025 to 2050. **e-h**, Comparison of carbon footprint for the US manufacturing scenario under BT1, BT2, BT3, and BT4, respectively, and full-pricing EV market penetration from 2025 to 2050.

Projection based on EV market expansion and battery technology penetration

In this study, we also examine the impacts of EV market expansion level, battery technology penetration, and degrees of production onshore and at the allies over 2020–2050 on the environmental performances of supply chain restructuring. For the variation in degrees of production onshore and at the allies, we follow the settings of US manufacturing and ally-shoring scenarios with 20%, 40%, 60%, 80%, or 100% production onshore or at the allies. Specifically, only the restructuring of midstream and downstream supply chains is involved. Besides the different degrees of production onshore and at the allies, the rest follows a BAU supply chain setting as it is under the 2019 US EV fleet case. We adopt the projection of EV market expansion and battery technology penetration from existing literature (*2, 12*). The projection of battery technology penetration for the US follows the battery cathode technology scenario setting from a dynamic material flow analysis with a regional resolution to address the future cobalt supply risks. Specifically, BT1, BT2, and BT3 represent the gradual adoption of the state-of-the-art NMC811 and NCA, advanced low-cobalt NMC955 and second-generation NCA, and no-cobalt blade LFP with the cell-to-pack technology, respectively, from 2020, and reach complete substitution in the market by 2050. BT4 characterizes the gradual adoption of the state-of-the-art NMC811 and NCA until 2030 and rapid market dominance of next-generation cobalt-free battery technologies, such as Li-S, Li-air, and solid-state batteries, from 2030 to 2050. However, solid-state batteries are excluded from this study due to challenges in obtaining life cycle inventories of their diverse battery systems (*65*). The projection of different battery cathode technology scenarios for every five years from 2025 to 2050 can be found in Fig. S6. The projection of EV market expansion until 2050 focuses on the US light-duty vehicle sector with both demand-side solutions and the responses from the supply side (upstream material production, vehicle manufacturing, and fuel generation) using a US-specific integrated energy model. Two carbon emission regulation strategies are considered, with only tailpipe emissions or full life cycle emissions priced. These projections show an accelerated transition toward the BEV-dominant future. The projected share of PHEVs among the total energy capacity of all EVs is under 3% from 2025 to 2050 under both carbon emission regulation strategies and thus are not considered in this study. Furthermore, the projection leans towards adopting short-distance BEVs when full life cycle CO_2 emissions are priced, as shown in Fig. S7.

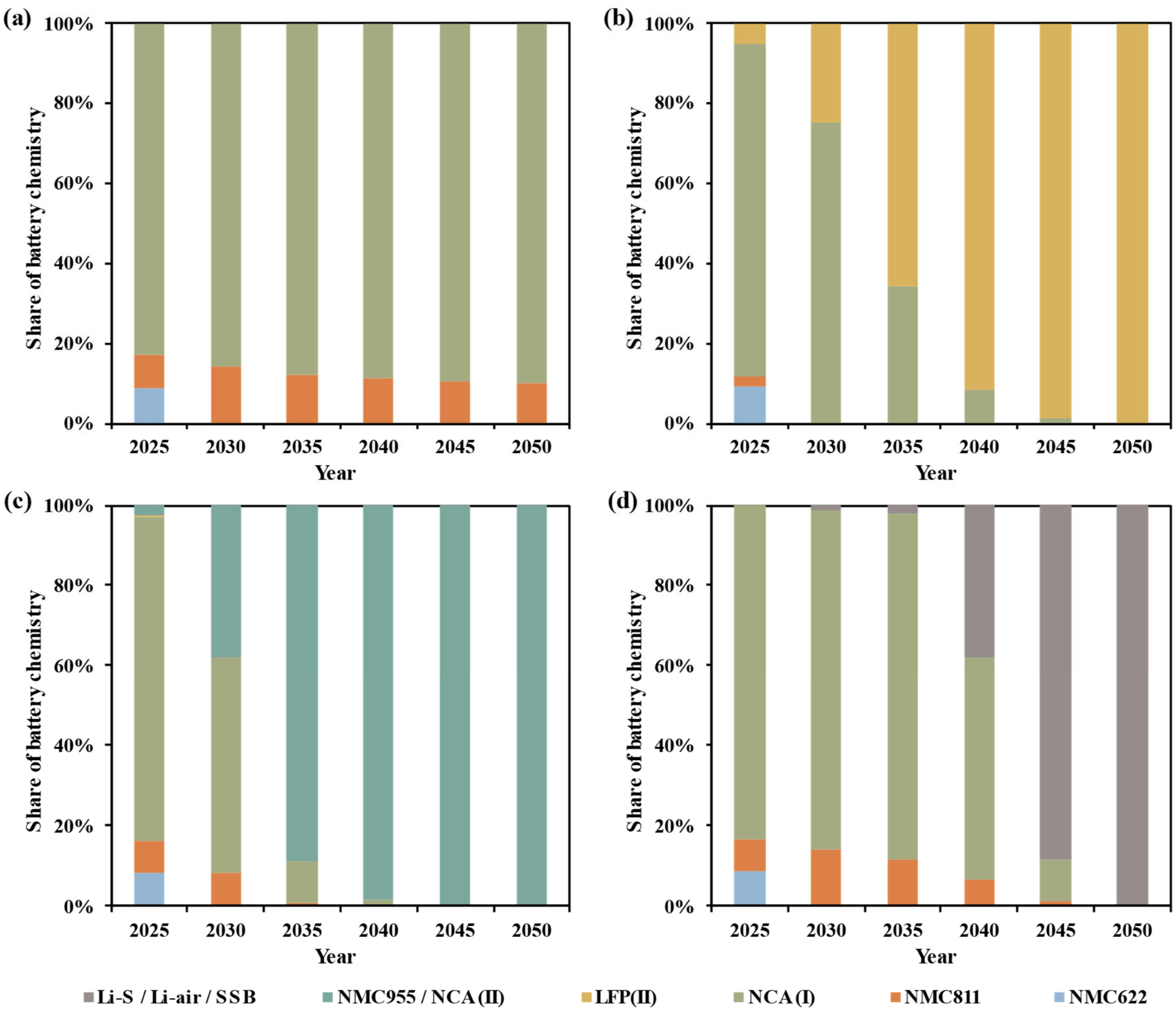

Fig. S6 Illustration of battery technology penetration trends. a-d, Battery technology penetration under BT1, BT2, BT3, and BT4 scenarios, respectively from 2025 to 2050 (*12*).

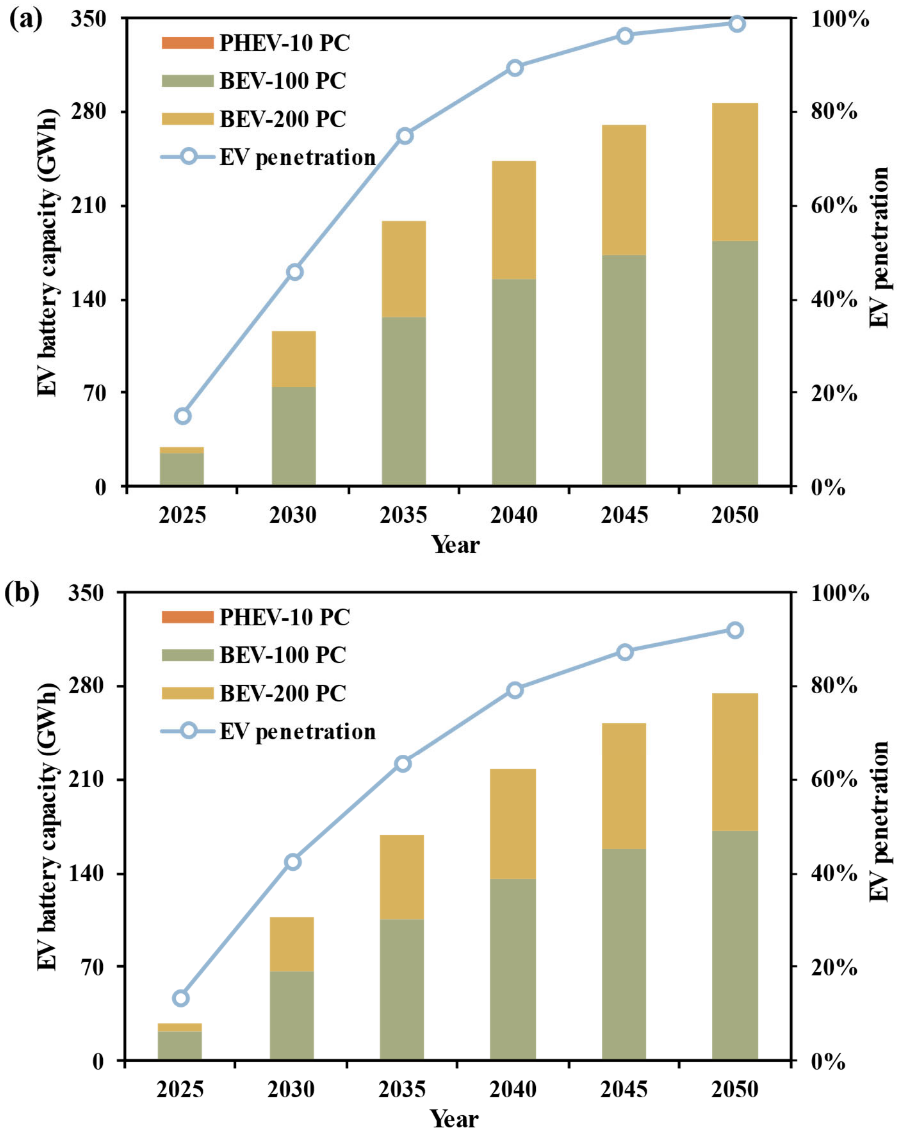

Fig. S7 Illustration of EV market expansion. a, EV market expansion under the full-pricing scenario. **b**, EV market expansion under the direct-emission-pricing only scenario.

Table S1 EV sales by the model in the US (*57*). OEMs for LIB cells and packs are identified (*60*). Production location of LIB cells, packs, and EV are listed (*60*). Models with less than 1,000 vehicles sold are not considered in this study for simplification.

Model	Type	2019 sales	Cell OEM	Pack OEM	Cell Production Location	Pack Production Location	Vehicle Assembly Location
Tesla Model 3	BEV	154,840	Panasonic	Tesla	Nevada, US	Nevada, US	California, US
Tesla Model X	BEV	19,425	Panasonic	Tesla	Japan	California, US	California, US
Chevy Bolt	BEV	16,313	LG Chem	LG Chem	Michigan, US	Michigan, US	Michigan, US
Tesla Model S	BEV	15,090	Panasonic	Tesla	Japan	California, US	California, US
Nissan Leaf	BEV	12,365	AESC	AESC	Tennessee, US	Tennessee, US	Tennessee, US
Audi e-tron	BEV	5,369	LG Chem	LG Chem	Poland	Belgium	Belgium
VW e-Golf	BEV	4,863	Samsung	Samsung SDI	Korea	Hungary	Germany
BMW i3	BEV	4,854	Samsung	Samsung SDI	Hungary	Hungary	Germany
Jaguar I-Pace	BEV	2,594	LG Chem	Jaguar	Poland	Poland	Austria
Hyundai Kona Electric	BEV	1,721	LG Chem	LG Chem	Korea	Korea	Korea
Kia Niro EV	BEV	1,562	SK Innovation	SK Innovation	Korea	Korea	Korea
Toyota Prius PHEV	PHEV	23,630	Panasonic	Panasonic	Japan	Japan	Japan
Honda Clarity Plug-in	PHEV	10,728	Panasonic	Panasonic	Japan	Japan	Japan
Ford Fusion Energi	PHEV	7,476	Panasonic	Panasonic	MI, US	MI, US	Mexico
Chrysler Pacifica Plug-in Hybrid	PHEV	5,811	LG Chem	LG Chem	MI, US	MI, US	Canada
BMW 5-Series Plug in (530e)	PHEV	5,442	Samsung	BMW Group	Korea	Germany	Austria
Chevy Volt	PHEV	4,915	LG Chem	LG Chem	MI, US	MI, US	MI, US
Kia Niro Plug In	PHEV	4,051	SK Innovation	SK Innovation	Korea	Korea	Korea
Mitsubishi Outlander Plug In	PHEV	2,810	GS Yuasa	GS Yuasa	Japan	Japan	Japan
Mercedes GLC 350e Hybrid	PHEV	2,459	SK Innovation	Accumotive	Korea	Germany	Germany
Mercedes C350We	PHEV	2,172	SK Innovation	Accumotive	Korea	Germany	AL, U.S.
Porsche Panamera S E-Hybrid	PHEV	1,958	Panasonic	Bosch	Japan	Germany	Germany
Hyundai Ioniq Plug-In	PHEV	1,765	LG Chem	LG Chem	Korea	Korea	Korea
Volvo-XC90 Plug In	PHEV	1,710	LG Chem	LG Chem	Poland	Poland	Sweden
Volvo XC60 Plug In	PHEV	1,682	LG Chem	LG Chem	Poland	Poland	Sweden

Mercedes GLE 550e	PHEV	1,509	SK Innovation	Accumotive	Korea	Germany	AL, U.S.
Porsche Cayenne S E-Hybrid	PHEV	1,140	Panasonic	Bosch	Japan	Germany	Germany
BMW i8	PHEV	1,102	Samsung	Samsung SDI	Korea	Germany	Germany

Table S2 EV sales by the model in the United States (*57*). Battery capacity for each model is obtained from an Argonne National Laboratory study (*60*). Electric range and energy efficiency are extracted from fueleconomy.gov (*59*). Battery chemistry data are obtained from both existing journal articles and industry reports (*11, 66-69*). As lithium nickel manganese cobalt oxide (NMC622) dominates the battery chemistry found for the US BEV fleet, all NMC LIBs with unknown compositions of nickel, manganese, and cobalt are assumed to be NMC622. Acronyms: lithium nickel cobalt aluminum oxide (NCA); lithium manganese oxide (LMO).

Vehicle	Type	2019 sales	Capacity (kWh)	Electric range (mile)	Energy efficiency (Wh/mile)	Chemistry
Tesla Model 3	BEV	154,840	54	220	260	NCA
Tesla Model X	BEV	19,425	100	295	390	NCA
Chevy Bolt	BEV	16,313	60	238	280	NMC622
Tesla Model S	BEV	15,090	75	285	310	NCA
Nissan Leaf	BEV	12,365	40	150	300	NMC622
Audi e-tron	BEV	5,369	95	204	460	NMC622
VW e-Golf	BEV	4,863	35.8	125	280	NMC532
BMW i3	BEV	4,854	42.2	153	300	NMC622
Jaguar I-Pace	BEV	2,594	90	234	440	NMC
Hyundai Kona Electric	BEV	1,721	64	258	280	NMC622
Kia Niro EV	BEV	1,562	64	239	300	NMC622
Toyota Prius PHEV	PHEV	23,630	8.8	22	450	NMC
Honda Clarity Plug-in	PHEV	10,728	17	48	310	NMC
Ford Fusion Energi	PHEV	7,476	7.6	26	330	LMO/NMC
Chrysler Pacifica Plug-in Hybrid	PHEV	5,811	16	32	410	NMC
BMW 5-Series Plug in (530e)	PHEV	5,442	9.2	16	460	LMO/NMC
Chevy Volt	PHEV	4,915	18.4	53	310	LMO/NMC
Kia Niro Plug In	PHEV	4,051	8.9	26	320	NMC
Mitsubishi Outlander Plug In	PHEV	2,810	12	22	450	NMC

Mercedes GLC 350e Hybrid	PHEV	2,459	8.7	10	590	NMC
Mercedes C350We	PHEV	2,172	6.2	9	560	NCA
Porsche Panamera S E-Hybrid	PHEV	1,958	9.4	14	650	NMC
Hyundai Ioniq Plug-In	PHEV	1,765	8.9	29	280	NMC
Volvo-XC90 Plug In	PHEV	1,710	9.8	17	550	NMC
Volvo XC60 Plug In	PHEV	1,682	10.4	17	550	NMC
Mercedes GLE 550e	PHEV	1,509	8.7	10	690	NMC
Porsche Cayenne S E-Hybrid	PHEV	1,140	10.8	14	700	NMC
BMW i8	PHEV	1,102	7.8	18	490	NMC

Table S3 Production capacity of LIB components by region (*17*). As the European Union (EU) is the largest LIB cell and component producer, following China, Japan, Korea, and the US (*70*). We attribute all rest-of-world (RoW) production capacity to the EU for simplification.

Region	Cathode	Anode	Electrolyte	Separator
US		10%	2%	6%
China	42%	65%	65%	43%
Japan	33%	19%	12%	21%
Korea	15%	6%	4%	28%
RoW/EU	10%		17%	2%

Table S4 Production capacity of valuable metal mining and refining by country (*17, 21*). Acronyms: Democratic Republic of the Congo (DRC). The values of each column do not necessarily sum to 100% since only the top four producers are identified.

Region	Lithium mining	Lithium refining	Cobalt mining	Cobalt refining	Nickel mining	Nickel refining
Australia	60%		4%		6%	
Argentina	7%	10%				
Canada				4%	7%	13%
Chile	19%	30%				
China	9%	60%		72%	5%	16%
Cuba			4%			
DRC			70%			
Finland				9%		
Indonesia					26%	
Japan						15%
New Caledonia					10%	
Norway				4%		
Philippines					16%	
Russia			5%		10%	21%

Table S5 Percentage of primary and secondary sources for US production and import of metals (*21*).

	Aluminum	Copper	Steel	Nickel	Cobalt	Lithium
Domestic: secondary	34%	25%	67%	39%	19%	
Domestic: primary	44%	40%	12%	12%	3%	75%
Import: secondary	8%	5%	4%	12%	10%	
Import: primary	14%	30%	17%	37%	68%	25%

Table S6 Reallocated production capacity of LIB components by region for the ally-shoring scenario (*17*). The production capacity of China is allocated to other regions proportional to their current production capacities. The production capacity of LIB cells and packs are not reallocated for the ally-shoring scenario because no LIB cell or pack of the US EV fleet with over 1,000-vehicle sales is produced in China.

Region	Cathode	Anode	Electrolyte	Separator
US		29%	6%	11%
China				
Japan	57%	54%	34%	37%
Korea	26%	17%	11%	49%
EU	17%		49%	4%

Interrupted nickel supply and US domestic sourcing of critical materials

Given the recent geopolitical tensions over the global critical material supply chain, we conduct sensitivity analysis on the nickel supply from Russia for both scenarios and domestic sourcing of critical materials for the US manufacturing scenario. We assess the impact of no nickel supply from Russia by reallocating its shares of cobalt and nickel mining capacities as well as the nickel refining capacity to other top miners and refiners mentioned in Table S7. Moreover, we model the impact of 100% domestic sourcing of critical materials, including lithium, nickel, and cobalt, by adapting the mining and refining processes of Canada to the US. Specifically, NERC regions where mines are located in the US are selected for the power grid; Transportation distances are recalculated. According to the US's recent Defense Production Act, lithium is projected to be mainly produced from geothermal brine, with about 400,000 t lithium carbonate equivalent annual production capacity from Berkshire Hathaway Energy Renewables, Controlled Thermal Resources, and EnergySource Minerals (*71*). Meanwhile, the only operating lithium mine in the US produces 60,000 t lithium carbonate equivalent per year. Therefore, we adopt the life cycle inventory of lithium extraction using the geothermal brine, considering future demand growth and potential supply from geothermal brine based on existing works (*33, 72*). We consider a weighted sum of the environmental impacts of lithium produced from both the conventional pathway and geothermal brine, following their respective projected annual production capacities.

Table S7 Reallocated production capacity of valuable metal mining and refining by the country for the ally-shoring scenario (*17, 21*). The production capacity of China is allocated to other regions proportional to their current production capacities.

Region	Lithium mining	Lithium refining	Cobalt mining	Cobalt refining	Nickel mining	Nickel refining
Australia	70%		5%			
Argentina	8%	25%				
Canada				24%		27%
Chile	22%	75%				
China						
Cuba			5%			
DRC			84%			
Finland				53%		
Indonesia					42%	
Japan						31%
New Caledonia					16%	
Norway				24%		
Philippines					26%	
Russia			6%		16%	43%

Table S8 Material inventory of LIBs for BEV models in the 2019 US market (*61*).

Model		Audi e-tron	BMW i3	Chevy Bolt	Hyundai Kona Electric	Jaguar I-Pace	Kia Niro EV	Nissan Leaf	Tesla Model 3	Tesla Model S	Tesla Model X	VW e-Golf
Type		EV	EV	EV	EV	EV	EV	EV	EV	EV	EV	EV
Capacity (kWh)		95	42.2	60	64	90	64	40	54.00	75	100.00	35.80
Energy efficiency (Wh/mile)		460	300	280	280	440	300	300	260.00	310	390.00	280.00
Chemistry		NMC622	NMC622	NMC622	NMC622	NMC622	NMC622	NMC622	NCA	NCA	NCA	NMC532
Cell energy density (Wh/kg)		298	290	294	295	298	295	289	307.00	311	314.00	266.00
Pack energy density (Wh/kg)		234	203	219	222	233	221	200	223.00	237	247.00	184.00
Pack weight (kg)		515.10	256.14	342.88	362.18	490.69	362.60	245.32	301.12	399.33	515.53	238.63
Cathode	Active material (kg)	142.45	63.22	89.92	95.92	134.94	95.92	59.92	74.34	103.29	137.78	61.35
	Carbon black (kg)	2.97	1.32	1.87	2.00	2.81	2.00	1.25	1.55	2.15	2.87	1.28
	PVDF (kg)	2.97	1.32	1.87	2.00	2.81	2.00	1.25	1.55	2.15	2.87	1.28
	NMP (kg)	71.25	31.62	44.99	47.98	67.45	47.98	29.95	37.15	51.67	68.89	30.70
	Al current collector (kg)	8.45	3.93	5.46	5.81	8.03	5.81	3.74	4.45	6.07	7.97	3.73
Anode	Graphite (kg)	84.41	38.03	53.69	57.21	80.03	57.21	36.08	49.47	68.32	90.71	32.13
	PVDF (kg)	1.72	0.78	1.10	1.17	1.63	1.17	0.74	1.01	1.39	1.85	0.66
	NMP (kg)	41.36	18.60	26.32	27.99	39.17	27.99	17.68	24.25	33.47	44.41	15.72
	Cu current collector (kg)	19.83	9.35	12.92	13.72	18.84	13.72	8.90	10.56	14.32	18.73	8.90
Electrolyte	LiPF6 (kg)	7.59	3.41	4.82	5.13	7.20	5.13	3.23	4.16	5.75	7.64	2.98
	EC (kg)	13.99	6.27	8.88	9.46	13.26	9.46	5.95	7.66	10.59	14.08	5.49
	DMC (kg)	13.99	6.27	8.88	9.46	13.26	9.46	5.95	7.66	10.59	14.08	5.49
Separator	PP (kg)	2.18	1.00	1.40	1.49	2.07	1.49	0.95	1.14	1.56	2.05	1.18
	PE (kg)	0.13	0.06	0.08	0.09	0.12	0.09	0.06	0.07	0.09	0.12	0.07
Positive terminal	Al (kg)	2.59	1.63	2.02	2.09	2.52	2.09	1.58	1.85	2.21	2.62	1.54
Negative terminal	Cu (kg)	8.62	5.45	6.67	6.91	8.35	6.91	5.28	6.12	7.37	8.64	5.11
Cell container	Al (kg)	5.06	2.74	3.57	3.74	4.83	3.74	2.66	3.20	4.06	5.08	2.53
	PET (kg)	0.79	0.43	0.56	0.58	0.75	0.58	0.41	0.50	0.63	0.79	0.39
	PP (kg)	0.34	0.18	0.24	0.25	0.32	0.25	0.18	0.21	0.27	0.34	0.17

Module	Al casing (kg)	7.16	4.7	5.62	5.8	6.96	5.8	4.58	5.2	6.16	7.2	4.46
	Al conductor/ cooling plates (kg)	9.52	4.9	6.5	6.86	9.1	6.86	4.7	5.74	7.52	9.54	4.48
	Terminal (kg)	1.06	1.16	1.1	1.08	1.06	1.08	1.16	1.14	1.08	1.06	1.16
	Cell group interconnects (kg)	0.00	0.00	0.00	0.00	0.00	0.00	0.00	0.00	0.00	0.00	0.00
Pack	Al jacket outer layer (kg)	42.23	29.85	34.49	35.44	41.22	35.46	29.22	32.44	37.31	42.38	28.63
	Fiber glass jacket insulation layer (kg)	1.67	1.18	1.36	1.40	1.63	1.40	1.15	1.28	1.47	1.67	1.13
	Steel module compression plate (kg)	2.86	1.46	1.92	2.02	2.72	2.03	1.41	1.70	2.22	2.85	1.33
	Steel strap (kg)											
	Cu module interconnects (kg)	1.32	1.45	1.39	1.37	1.32	1.37	1.47	1.43	1.37	1.34	1.47
	BMS (kg)	3.60	3.60	3.60	3.60	3.60	3.60	3.60	3.60	3.60	3.60	3.60
	Coolant EG (kg)	7.25	5.95	5.66	5.65	7.09	5.85	5.97	5.70	6.17	6.92	5.67
	Coolant deionised water (kg)	7.25	5.95	5.66	5.65	7.09	5.85	5.97	5.70	6.17	6.92	5.67
	Pack terminal (kg)	0.12	0.13	0.13	0.12	0.12	0.12	0.13	0.13	0.12	0.12	0.13

Table S9 Material inventory of LIBs for PHEV models in the 2019 US market (*61*) (Part I).

**Model**	**BMW 5-Series Plug in (530e)**	**BMW i8**	**Chevy Volt**	**Chrysler Pacifica Plug-in Hybrid**	**Ford Fusion Energi**	**Honda Clarity Plug-in**	**Hyundai Ioniq Plug-In**	**Kia Niro Plug In**	**Mercedes C350We**
Type	PHEV	PHEV	PHEV	PHEV	PHEV	PHEV	PHEV	PHEV	PHEV
Capacity (kWh)	9.2	7.8	18.4	16	7.6	17	8.9	8.9	6.2
Energy efficiency (Wh/mile)	460	490	310	410	330	310	280	320	560
Chemistry	LMO/ NMC532	NMC622	LMO/ NMC532	NMC622	LMO/ NMC532	NMC622	NMC622	NMC622	NCA

	Cell energy density (Wh/kg)	207	223	216	258	199	259	231	231	211
	Pack energy density (Wh/kg)	112	112	139	155	104	161	123	122	107
	Pack weight (kg)	99.29	76.73	169.06	120.49	86.96	124.02	80.84	81.53	63.14
Cathode	Active material (kg)	18.07	11.61	36.16	23.93	14.92	25.42	13.26	13.26	8.46
	Carbon black (kg)	0.38	0.24	0.75	0.50	0.31	0.53	0.28	0.28	0.18
	PVDF (kg)	0.38	0.24	0.75	0.50	0.31	0.53	0.28	0.28	0.18
	NMP (kg)	9.04	5.82	18.09	11.98	7.43	12.73	6.62	6.62	4.20
	Al current collector (kg)	1.44	1.71	2.64	1.75	1.48	1.86	1.67	1.67	1.84
Anode	Graphite (kg)	8.35	7.25	16.36	14.61	6.93	15.50	8.25	8.25	5.92
	PVDF (kg)	0.44	0.15	0.86	0.30	0.36	0.32	0.17	0.17	0.12
	NMP (kg)	10.54	3.57	20.68	7.14	8.76	7.60	4.03	4.03	2.88
	Cu current collector (kg)	3.53	4.19	6.34	4.26	3.61	4.49	4.09	4.09	4.52
Electrolyte	LiPF6 (kg)	1.38	1.06	2.72	2.00	1.17	2.11	1.18	1.18	0.87
	EC (kg)	2.55	1.96	5.02	3.68	2.16	3.90	2.17	2.17	1.60
	DMC (kg)	2.55	1.96	5.02	3.68	2.16	3.90	2.17	2.17	1.60
Separator	PP (kg)	0.37	0.44	0.68	0.45	0.38	0.48	0.43	0.43	0.47
	PE (kg)	0.02	0.03	0.04	0.03	0.02	0.03	0.03	0.03	0.03
Positive terminal	Al (kg)	0.77	0.65	1.18	0.96	0.70	1.01	0.70	0.70	0.55
Negative terminal	Cu (kg)	2.54	2.14	3.89	3.19	2.28	3.31	2.28	2.28	1.85
Cell container	Al (kg)	1.44	1.24	2.27	1.82	1.30	1.91	1.29	1.29	1.08
	PET (kg)	0.22	0.19	0.35	0.28	0.20	0.30	0.20	0.20	0.17
	PP (kg)	0.10	0.08	0.15	0.12	0.09	0.13	0.09	0.09	0.07
Module	Al casing (kg)	2.46	2.18	3.4	2.92	2.3	3	2.3	2.3	2.02
	Al conductor/cooling plates (kg)	2.1	1.72	3.62	2.8	1.86	2.94	1.86	1.86	1.48
	Terminal (kg)	0.6	0.62	0.52	0.58	0.6	0.58	0.62	0.62	0.62
	Cell group interconnects (kg)	0.00	0.00	0.00	0.00	0.00	0.00	0.00	0.00	0.00
Pack	Al jacket outer layer (kg)	12.85	11.66	21.74	14.67	12.07	14.92	12.10	12.14	7.37
	Fiber glass jacket insulation layer (kg)	0.51	0.46	0.86	0.58	0.48	0.59	0.48	0.48	0.29
	Steel module compression plate (kg)	0.79	0.66	1.19	1.01	0.69	1.01	0.67	0.69	0.58
	Steel strap (kg)									
	Cu module interconnects (kg)	0.74	0.78	0.65	0.74	0.74	0.74	0.78	0.74	0.78
	BMS (kg)	3.60	3.60	3.60	3.60	3.60	3.60	3.60	3.60	3.60

Coolant EG (kg)	5.64	5.13	4.62	6.08	4.90	5.17	4.49	4.83	4.79
Coolant deionised water (kg)	5.64	5.13	4.62	6.08	4.90	5.17	4.49	4.83	4.79
Pack terminal (kg)	0.07	0.07	0.06	0.07	0.07	0.07	0.07	0.07	0.07

Table S10 Material inventory of LIBs for PHEV models in the 2019 US market (*61*) (Part II).

	**Model**	**Mercedes GLC 350e Hybrid**	**Mercedes GLE 550e**	**Mitsubishi Outlander Plug In**	**Porsche Cayenne S E-Hybrid**	**Porsche Panamera S E-Hybrid**	**Toyota Prius PHEV**	**Volvo XC60 Plug In**	**Volvo-XC90 Plug In**
	Type	PHEV	PHEV	PHEV	PHEV	PHEV	PHEV	PHEV	PHEV
	Capacity (kWh)	8.7	8.7	12	10.8	9.4	8.8	10.4	9.8
	Energy efficiency (Wh/mile)	590	690	450	700	650	450	550	550
	Chemistry	NMC622	NMC622	NMC622	NMC622	NMC622	NMC622	NMC622	NMC622
	Cell energy density (Wh/kg)	230	230	249	243	235	231	241	237
	Pack energy density (Wh/kg)	118	118	138	131	123	120	129	126
	Pack weight (kg)	81.87	81.79	99.05	93.16	85.58	82.06	90.77	87.46
Cathode	Active material (kg)	12.96	12.96	17.92	16.11	14.01	13.11	15.51	14.61
	Carbon black (kg)	0.27	0.27	0.37	0.34	0.29	0.27	0.32	0.30
	PVDF (kg)	0.27	0.27	0.37	0.34	0.29	0.27	0.32	0.30
	NMP (kg)	6.45	6.45	8.99	8.06	7.03	6.57	7.78	7.32
	Al current collector (kg)	1.68	1.68	1.58	1.61	1.65	1.67	1.62	1.64
Anode	Graphite (kg)	8.07	8.07	11.04	9.96	8.70	8.15	9.60	9.06
	PVDF (kg)	0.17	0.17	0.23	0.20	0.18	0.17	0.20	0.18
	NMP (kg)	3.97	3.97	5.41	4.90	4.26	3.97	4.72	4.44
	Cu current collector (kg)	4.11	4.11	3.87	3.94	4.04	4.09	3.96	4.00
Electrolyte	LiPF6 (kg)	1.16	1.16	1.52	1.39	1.24	1.17	1.35	1.28
	EC (kg)	2.14	2.14	2.81	2.56	2.28	2.15	2.48	2.36
	DMC (kg)	2.14	2.14	2.81	2.56	2.28	2.15	2.48	2.36
Separator	PP (kg)	0.43	0.43	0.41	0.41	0.42	0.43	0.41	0.42
	PE (kg)	0.03	0.03	0.02	0.02	0.03	0.03	0.02	0.03
Positive terminal	Al (kg)	0.67	0.67	0.82	0.77	0.72	0.67	0.74	0.72
Negative terminal	Cu (kg)	2.26	2.26	2.69	2.54	2.35	2.28	2.50	2.40
Cell container	Al (kg)	1.28	1.28	1.54	1.44	1.34	1.30	1.43	1.38
	PET (kg)	0.20	0.20	0.24	0.22	0.21	0.20	0.22	0.21

	PP (kg)	0.09	0.09	0.10	0.10	0.09	0.09	0.10	0.09
Module	Al casing (kg)	2.3	2.3	2.56	2.48	2.36	2.28	2.44	2.38
	Al conductor/cooling plates (kg)	1.84	1.84	2.28	2.1	1.92	1.86	2.06	1.98
	Terminal (kg)	0.7	0.62	0.62	0.76	0.62	0.62	0.62	0.62
	Cell group interconnects (kg)	0.00	0.00	0.00	0.00	0.00	0.00	0.00	0.00
Pack	Al jacket outer layer (kg)	12.13	12.13	13.23	12.93	12.41	12.08	12.73	12.49
	Fiber glass jacket insulation layer (kg)	0.48	0.48	0.52	0.51	0.49	0.48	0.50	0.49
	Steel module compression plate (kg)	0.70	0.70	0.85	0.79	0.73	0.71	0.78	0.75
	Steel strap (kg)								
	Cu module interconnects (kg)	0.78	0.78	0.78	0.78	0.78	0.78	0.78	0.78
	BMS (kg)	3.60	3.60	3.60	3.60	3.60	3.60	3.60	3.60
	Coolant EG (kg)	5.37	5.37	5.80	5.73	5.50	5.32	5.62	5.51
	Coolant deionised water (kg)	5.37	5.37	5.80	5.73	5.50	5.32	5.62	5.51
	Pack terminal (kg)	0.07	0.07	0.07	0.07	0.07	0.07	0.07	0.07

Table S11 Material inventory of LIBs for projection of battery technology penetration from 2025 to 2050 (*44, 45, 61*).

**Model**		**100-mile BEV**								**200-mile BEV**							
		**NMC 622**	**NMC 811**	**NCA (I)**	**Blade LFP**	**NMC 955**	**NCA 955**	**Li-S**	**Li-air**	**NMC 622**	**NMC 811**	**NCA (I)**	**Blade LFP**	**NMC 955**	**NCA 955**	**Li-S**	**Li-air**
Capacity (kWh)		29.5	29.5	29.5	28.9	29.5	29.5	33.5	31.9	58.0	58.0	58.0	56.2	58.0	58.0	67.4	63.8
Energy efficiency (Wh/mile)		251	251	251	251	251	251	258	269	246	246	246	246	246	246	302	267
Cell energy density (Wh/kg)		285	295	299	221	303	303	286	242	294	304	308	226	312	312	330	359
Pack energy density (Wh/kg)		187	191	193	189	195	194	182	157	218	224	227	199	229	229	231	239
Pack weight (kg)		155.68	151.93	150.67	146.64	149.11	149.59	151.86	275.20	263.67	256.56	253.69	221.13	251.15	251.30	283.52	335.77
Cathode	Active material (kg)	44.18	37.48	40.59	59.92	36.14	39.61	16.50	7.04	86.90	73.71	79.84	117.79	71.08	77.90	33.60	14.45
	Carbon black (kg)	0.92	2.08	0.84	1.25	2.01	0.83	4.71	1.63	1.81	4.09	1.66	2.45	3.95	1.62	9.60	3.34
	PVDF (kg)	0.92	2.08	0.84	1.25	2.01	0.83	2.36	0.01	1.81	4.09	1.66	2.45	3.95	1.62	4.80	0.01
	NMP (kg)	22.12	50.00	20.28	29.96	48.21	19.81	56.56	115.20	43.43	98.27	39.92	58.90	94.75	38.94	6.56	13.20
	Al current collector (kg)	6.77	6.67	6.11	9.37	6.30	6.04	10.60	22.13	12.52	12.32	11.29	4.37	11.66	11.16	14.88	21.25
Anode	Graphite (kg)	26.81	27.69	27.40	30.58	27.73	27.41			51.93	53.65	53.06	16.84	53.71	53.08		
	Lithium anode (kg)							6.38	9.55							12.87	19.44
	Production layer (kg)							1.47	2.78							2.09	2.71
	PVDF (kg)	0.55	0.56	0.56	0.62	0.57	0.56			1.06	1.09	1.08	0.34	1.10	1.08		
	NMP (kg)	13.13	13.54	13.42	14.98	13.59	13.42			25.46	26.27	25.98	8.25	26.32	25.98		

	Cu current collector (kg)	6.77	6.67	6.11	9.37	6.30	6.04	19.52	40.67	12.52	12.32	11.29	4.37	11.66	11.16	27.39	39.05
Electrolyte	LiPF6 (kg)	2.39	2.36	2.29	4.02	2.29	2.27			4.66	4.60	4.46	7.73	4.47	4.43		
	EC (kg)	4.41	4.35	4.21	7.40	4.22	4.19			8.59	8.47	8.21	14.25	8.23	8.16		
	DMC (kg)	4.41	4.35	4.21	7.40	4.22	4.19			8.59	8.47	8.21	14.25	8.23	8.16		
	LiTFSI (kg)							5.81								11.18	
	DOL (kg)							10.54								20.27	
	DME (kg)							19.62								37.74	
	LiNO3 (kg)							0.36								0.70	
	LiClO4 (kg)								4.43								7.57
	TEGDME (kg)								34.73								51.75
Separator	PP (kg)	0.71	0.86	0.64	1.10	0.81	0.63	4.58	7.32	1.35	1.63	1.22	1.84	1.54	1.20	6.43	7.08
	PE (kg)	0.18	0.21	0.16	0.28	0.20	0.16	1.14	1.83	0.34	0.41	0.30	0.46	0.38	0.30	1.61	1.77
Positive terminal	Al (kg)	1.32	1.34	1.30	0.09	1.32	1.30	1.95	1.86	1.97	1.97	1.92	0.34	1.94	1.92	2.76	2.44
Negative terminal	Cu (kg)	4.42	4.44	4.30	0.31	4.34	4.30	6.44	6.14	6.53	6.55	6.38	1.12	6.46	6.36	9.13	8.05
Cell container	Al (kg)	2.15	2.16	2.08	1.13	2.14	2.06	4.43	7.36	3.49	3.50	3.36	1.89	3.43	3.31	7.48	11.05
	PET (kg)	0.33	0.34	0.32	0.18	0.33	0.32	0.69	1.14	0.54	0.54	0.52	0.29	0.53	0.52	1.16	1.72
	PP (kg)	0.14	0.14	0.14	0.08	0.14	0.14	0.30	0.49	0.23	0.23	0.22	0.13	0.23	0.22	0.50	0.74
Module	Al casing (kg)	3.96	3.96	3.88	0.00	3.9	3.88	4.43	4.32	5.52	5.54	5.4	0.00	5.44	5.38	6.14	5.57
	Al conductor/ cooling plates (kg)	3.7	3.74	3.56	2.25	3.66	3.54	7.77	6.63	6.32	6.4	6.08	3.657	6.24	6.04	13.56	10.24
	Terminal (kg)	0.68	0.66	0.68	0.00	0.68	0.68	0.65	0.48	1.1	1.1	1.12	0.00	1.1	1.12	0.65	0.48
	Cell group interconnects (kg)	0.00	0.00	0.00	0.00	0.00	0.00	1.72	1.85	0.00	0.00	0.00	0.00	0.00	0.00	2.43	2.42
Oxygen tank (kg)									55.00								55.00
Pack	Al jacket outer layer (kg)	25.91	25.92	25.54	7.95	25.62	25.57	28.52	31.02	33.97	34.00	33.40	15.23	33.57	33.42	37.84	38.61
	Fiber glass jacket insulation layer (kg)	1.02	1.02	1.01	0.31	1.01	1.01	1.13	1.23	1.34	1.34	1.32	0.60	1.33	1.32	1.49	1.53
	Steel module compression plate (kg)	1.11	1.12	1.08	0.00	1.10	1.07	1.43	1.60	1.85	1.87	1.79	0.00	1.83	1.77	2.50	2.46
	Steel strap (kg)																
	Cu module interconnects (kg)	0.84	0.84	0.86	0.00	0.84	0.86	0.82	0.70	1.39	1.39	1.41	0.00	1.39	1.41	0.82	0.70
	BMS (kg)	3.60	3.60	3.60	3.10	3.60	3.60	4.00	4.00	3.60	3.60	3.60	3.10	3.60	3.60	4.00	4.00
	Coolant EG (kg)	5.57	5.43	5.82	1.14	5.50	5.88	3.59	9.39	5.31	5.19	5.53	1.74	5.24	5.58	4.76	10.93
	Coolant deionised water (kg)	5.57	5.43	5.82	1.14	5.50	5.88	3.59	9.39	5.31	5.19	5.53	1.74	5.24	5.58	4.76	10.93
	Pack terminal (kg)	0.08	0.08	0.08	1.48	0.08	0.08	0.19	0.29	0.13	0.13	0.13	1.48	0.13	0.13	0.19	0.29

Table S12 Energy inventory of LIB production (*8, 62*). Cathode and anode production include pumping, coating, coiling, cutting, and heating. Cell production involves drying and calendaring of anode, cathode, and separator.

	**Process**	**PHEV**			**EV**		**100-mile BEV**									**200-mile BEV**							
		**LMO/ NMC532**	**NMC 622**	**NCA (I)**	**NMC 622**	**NCA (I)**	**NMC 532**	**NMC 622**	**NMC 811**	**NCA (I)**	**Blade LFP**	**NMC 955**	**NCA 955**	**Li-S**	**Li-air**	**NMC 622**	**NMC 811**	**NCA (I)**	**Blade LFP**	**NMC 955**	**NCA 955**	**Li-S**	**Li-air**
Heat (MJ/kg)	Cathode production											0.65											
	Anode production											1.22											
	Cell production											0.065											
	Dry room operation											1.28											
Electricity (kWh/kg)	Cathode production											0.002											
	Anode production											0.002											
	Cell production											0.002											
	Dry room operation											0.19											
	Cell charging	0.10	0.13	0.12	0.15	0.16	0.14	0.20	0.21	0.21	0.15	0.21	0.21	0.20	0.17	0.21	0.21	0.22	0.16	0.22	0.22	0.23	0.25
	Pack testing/activating	0.093	0.11	0.093	0.17	0.17	0.14	0.18	0.18	0.18	0.18	0.19	0.18	0.17	0.15	0.21	0.21	0.22	0.19	0.22	0.22	0.22	0.23

Table S13 Material and energy inventory of recovering 1 kg NMP (*62*).

	Amount
Decarbonised water (kg)	83.30
Nitrogen (kg)	0.015
Steam (kg)	1.53
Electricity (kWh)	0.05

Table S14 Percentage of primary and secondary sources for global production and import of metals (*34, 73*).

	Aluminum	Copper	Steel	Nickel	Cobalt	Lithium
Global: secondary	35%	15%	20%	1%	10%	0%
Global: primary	65%	85%	80%	99%	90%	100%

Table S15 Percentage of pyrometallurgical and hydrometallurgical recycling by region. We obtained the list of operational LIB recyclers that recover cobalt as salts from Sommerville et al. (*27*).

Region	Pyrometallurgical recycling	Hydrometallurgical recycling
China	3%	91%
EU	1%	1%
South Korea		3%
Canada		1%
Total	4%	96%

Table S16 Material and energy inventory of cobalt recycling with hydrometallurgical recycling of NMC333 (*23*). The mass allocation has been conducted for this multi-output process.

Process	Item	Pyrometallurgical recycling
Discharging	Electricity (kWh)	0.035
Smelting	Heat (MJ)	1.37
	Limestone (kg)	0.17
	Sand (kg)	0.20
	Slag (kg)	1.34
	Coke (kg)	38.18
	CO_2 emissions (kg)	2.90
	PET incineration (kg)	0.0024
	PP incineration (kg)	0.0097
	PE incineration (kg)	0.0022
	PVDF incineration (kg)	0.031
	NMP evaporation (kg)	0.015
Leaching	Heat (MJ)	0.074
	HCl (kg)	1.28
Cu precipitation	Fe (kg)	0.105
Geothite precipitation	$NaClO_3$ (kg)	0.038
	Na_2CO_3 (kg)	0.23
Solvent extraction	H_2SO_4 (kg)	1.57
	H_2O_2 (kg)	0.24
Precipitation	NaOH (kg)	0.64
	Deionized water (kg)	13.80
Wastewater treatment	wastewater (kg)	17.94

Table S17 Material and energy inventory of cobalt recycling with pyrometallurgical recycling of NMC333 (*23*). The mass allocation has been conducted for this multi-output process.

Process	Item	Hydrometallurgical recycling
discharging	Electricity (kWh)	0.021
Crushing	Electricity (kWh)	0.036
Waste treatment	PVDF incineration (kg)	0.026
NMP soaking	Heat (MJ)	0.22
	NMP (kg)	0.018
NMP recovery	Decarbonised water (kg)	73.37
	Wastewater treatment (kg)	0.074
	Nitrogen (kg)	0.015
	Steam (kg)	1.35
	Steam generation facility (unit)	3.47E-11
Calcination	Heat (MJ)	1.89
	Electricity (kWh)	0.021
Leaching	Heat (MJ)	0.012
	H_2O_2 (kg)	0.31
	H_2SO_4 (kg)	2.04
	HCl (kg)	0.033
precipitation	Soda ash (kg)	0.46
	NaOH (kg)	0.39
	Deionized water (kg)	8.90
Wastewater treatment	wastewater (kg)	11.16

Table S18 Percentage of sources for global primary copper and secondary aluminum (*35, 37*).

	Aluminum		Copper
Secondary: Pre-customer scrap	41%	Primary: Electrowinning	22%
Secondary: Post-customer scrap	59%	Primary: Electrorefining	78%

Manufacturing cost and cost prospects of supply chain restructuring for the US case

Economic analysis is performed to quantify the manufacturing cost of LIBs imported from different regions. Breakdowns of LIB manufacturing costs are extracted from the BatPac model, which includes material cost, labor cost, capital equipment cost, and building cost (detailed in **Table S20**–S**Table** S22) (*61*). In particular, the production costs are estimated based on plants with annual throughput of 100,000 battery packs following the settings in the BatPac model. Moreover, material cost, building cost, and labor cost are computed using region-specific data, as illustrated in **Table S23–STable** S24. Labor costs for each country are obtained from government statistics (*74-76*). For building cost, we obtain the unit cost in Shanghai from the market and estimate for other regions involved in producing LIB components, cells, and packs using the 2017 price level index (PLI) of the construction sector (*77, 78*). PLIs are extracted from the International Comparison Program of the World Bank, and inflation is considered. BatPac model assumes direct purchase of cathode active materials (*61*). In this study, we source the valuable metals for the cathode active material production in regions listed in **Table S3**. Therefore, there is a need to model the cathode active material production while capturing the region-specific differences in costs. Following a previous study, we model the production costs of cathode active materials for NMC622, NCA, NMC532, and LMO/NMC532, considering capital equipment cost, building cost, material cost, labor cost, maintenance cost, and energy cost (*79*). Similar to the LIB manufacturing cost estimation, region-specific material costs, building costs, and labor costs are adopted. The unit prices of raw materials for the cathode active material production are computed using the trade data (i.e., net value and weight imported by the region of interest) extracted from the UN Comtrade database (*63*). The prices of other raw materials are set constant regardless of the exporting and importing regions due to the limited data availability. The result of cathode active material cost is listed in **Table S25**. Moreover, we evaluated how the skyrocketed nickel price would raise the LIB manufacturing cost using the record-high nickel price of \$48/kg (*80*). The unit prices of $NiSO_4$ and $CoSO_4$ exported from Canada to the US are used as proxies for the prices of domestic originated metals (*63*). We adopt the projected lithium price considering future demand growth and potential supply from geothermal brine based on existing works (*33, 66*).

Fig. S8 compares the manufacturing costs of LIBs by battery chemistry for the 2019 US EV fleet. This suggests that LIBs with higher specific energy density tend to attain lower unit manufacturing costs, although the cost advantages are less significant when battery pack energy capacities are low. **Table S19** lists the average labor cost in different countries producing the LIB components, cells, and packs. The region-specific material cost of cathode active material production (\$/kg), labor cost, and building cost are available in **Table S23–STable** S24 (*63, 74-76*). The manufacturing cost of LIBs for EV models in the 2019 US market is calculated using the BatPac model (*61*), as shown in **Table S20**–S**Table** S22. **Table S25** estimates the region-specific cathode active material cost using the cost model from Ciez et al. (*81*).

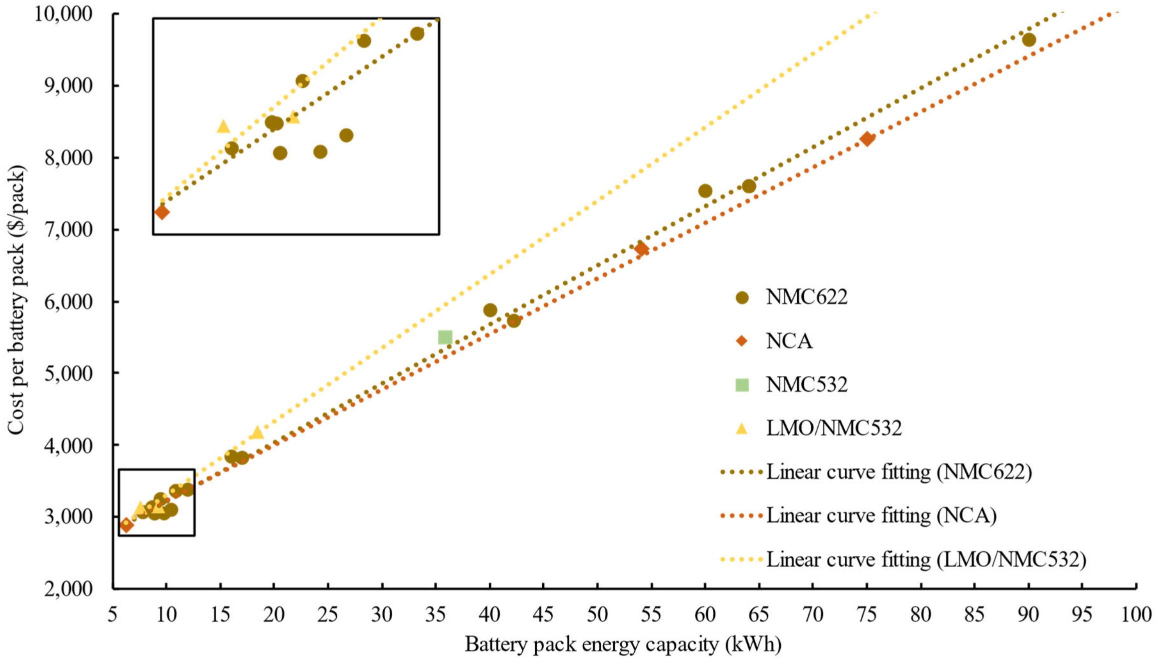

Fig. S8 Economic performance of LIBs for the 2019 US EV fleet. Each marker represents the LIB manufacturing cost of an EV model. Each dotted line denotes the linear curve fitting for each battery chemistry.

Table S19 Average labor cost in Belgium, Hungary, Poland, Germany, Korea, Japan, US, China, and EU, where LIB components, cells, and packs are produced.

Region	Labour cost ($/person-hour)	Source
Belgium	45.34	(*74*)
Hungary	11.08	(*74*)
Poland	11.98	(*74*)
Germany	39.86	(*74*)
Korea	16.78	(*75*)
Japan	21.29	(*76*)
US	31.46	(*82*)
China	5.41	(*83*)
EU	31.91	(*74*)

Table S20 Manufacturing cost of LIBs for BEV models in the 2019 US market (*61*).

Cost	Material		Audi e-tron	BMW i3	Chevy Bolt	Hyundai Kona Electric	Jaguar I-Pace	Kia Niro EV	Nissan Leaf	Tesla Model 3	Tesla Model S	Tesla Model X	VW e-Golf
	Type		EV	EV	EV	EV	EV	EV	EV	EV	EV	EV	EV
	Capacity (kWh)		95	42.2	60	64	90	64	40	54	75	100	35.8
	Energy efficiency (Wh/mile)		460	300	280	280	440	300	300	260	310	390	280
	Chemistry		NMC 622	NMC 622	NMC 622	NMC 622	NMC 622	NMC 622	NMC 622	NCA	NCA	NCA	NMC 532
Materials ($/pack)	Positive active material	US EV fleet case	3837	1703	2422	2584	3635	2584	1614	1934	2687	3585	1623
		US manufacturing scenario	5148	2285	3249	3466	4876	3466	2165	2598	3610	4816	2184
		Ally-shoring scenario	4758	2112	3003	3204	4507	3204	2002	2243	3116	4157	2022
		China	2566	1139	1619	1728	2430	1728	1079	1508	2095	2795	1072
		Japan	5327	2364	3362	3587	5046	3587	2241	2450	3404	4541	2267
		South Korea	3648	1619	2303	2456	3455	2456	1535	1856	2579	3441	1545
		EU	4547	2018	2870	3062	4307	3062	1913	2138	2970	3962	1929
	Negative active material		1182	554	769	817	1124	817	527	711	967	1266	472
	Carbon black		75	34	48	51	72	51	32	41	56	75	32
	Positive current collector		73	34	47	50	69	50	32	38	52	69	32
	Negative current collector		310	146	202	214	294	214	139	165	224	293	139
	Separator		477	219	306	325	452	325	208	248	340	450	207
	Electrolyte		498	223	316	337	472	337	212	272	377	501	195
	Cell hardware		249	217	229	231	246	231	216	224	236	249	214
	Module hardware		1008	839	896	909	992	909	832	879	947	1027	822
	Battery jacket		587	464	501	509	579	509	459	485	525	588	454
	BMS		540	540	540	540	540	540	540	540	540	540	540
	Thermal management system		80	80	40	40	80	80	80	80	80	80	80
	Purchased items ($/pack)		1966	1622	1735	1759	1937	1760	1607	1693	1821	1988	1589
Labor ($/pack)	US EV fleet case		529	234	547	345	306	345	503	533	489	527	272
	US manufacturing scenario		780	622	683	695	768	695	614	658	721	785	602
	Ally-shoring scenario		618	294	618	418	393	418	561	598	566	617	328
	China		381	272	314	322	372	322	266	295	338	382	259
	Japan		536	428	470	478	528	478	422	453	496	540	414
	South Korea		416	332	364	371	409	371	327	351	385	419	321
	EU		989	788	865	881	973	881	778	834	913	995	763

Capital Equipment and Building ($/pack)	US EV fleet case	1129	879	1026	992	1104	992	932	990	1104	1199	869
	US manufacturing scenario	1196	944	1027	1045	1177	1045	933	991	1104	1200	921
	Ally-shoring scenario	1150	892	1041	1008	1123	1008	944	1004	1121	1220	880
	China	1126	886	965	981	1108	981	875	930	1039	1129	864
	Japan	1191	940	1023	1041	1172	1041	929	987	1100	1195	917
	South Korea	1100	872	946	962	1082	962	863	913	1017	1103	852
	EU	1146	907	986	1002	1128	1002	897	951	1060	1150	886

Table S21 Manufacturing cost of LIBs for PHEV models in the 2019 US market (*61*). (Part I).

Cost	Material		BMW 5-Series Plug in (530e)	BMW i8	Chevy Volt	Chrysler Pacifica Plug-in Hybrid	Ford Fusion Energi	Honda Clarity Plug-in	Hyundai Ioniq Plug-In	Kia Niro Plug In	Mercedes C350We
	Type		PHEV	PHEV	PHEV	PHEV	PHEV	PHEV	PHEV	PHEV	PHEV
	Capacity (kWh)		9.2	7.8	18.4	16	7.6	17	8.9	8.9	6.2
	Energy efficiency (Wh/mile)		460	490	310	410	330	310	280	320	560
	Chemistry		LMO/ NMC532	NMC622	LMO/ NMC532	NMC622	LMO/ NMC532	NMC622	NMC622	NMC622	NCA
Materials ($/pack)	Positive active material	US EV fleet case	352	313	704	645	290	685	357	357	220
		US manufacturing scenario	447	420	894	865	369	919	479	479	296
		Ally-shoring scenario	424	388	848	799	350	849	443	443	255
		China	252	209	504	431	208	458	239	239	172
		Japan	466	434	932	895	384	951	496	496	279
		South Korea	338	297	677	613	279	651	340	340	211
		EU	414	371	829	764	342	811	423	423	243
	Negative active material		131	115	249	223	110	236	130	130	95
	Carbon black		12	6	24	13	10	14	7	7	5
	Positive current collector		12	15	23	15	13	16	14	14	16
	Negative current collector		55	65	99	67	56	70	64	64	70
	Separator		80	96	149	99	82	104	93	93	102
	Electrolyte		91	70	179	131	77	139	77	77	57
	Cell hardware		191	187	204	197	189	198	189	189	185
	Module hardware		732	726	765	755	726	758	730	730	721
	Battery jacket		338	330	402	354	332	355	333	333	298
	BMS		540	540	540	540	540	540	540	540	540
	Thermal management system		280	240	120	280	200	160	160	200	240
	Purchased items ($/pack)		1346	1326	1462	1393	1330	1399	1335	1336	1283
Labor ($/pack)	US EV fleet case		332	326	439	431	397	317	236	236	318
	US manufacturing scenario		464	456	529	504	454	510	463	463	446
	Ally-shoring scenario		363	356	489	469	426	356	267	267	347
	China		167	163	213	191	161	196	167	167	157
	Japan		319	314	364	346	312	351	319	319	307
	South Korea		247	243	282	269	242	272	247	247	237

	EU	588	578	671	638	575	646	587	587	565
Capital Equipment and Building ($/pack)	US EV fleet case	721	713	809	797	744	802	715	715	702
	US manufacturing scenario	759	747	834	798	748	805	754	754	736
	Ally-shoring scenario	736	719	849	805	757	810	721	721	707
	China	712	701	783	749	702	755	708	708	691
	Japan	757	745	831	796	746	802	752	752	734
	South Korea	710	700	776	744	701	750	706	706	692
	EU	734	723	804	770	724	777	729	729	712

Table S22 Manufacturing cost of LIBs for PHEV models in the 2019 US market (*61*) (Part II).

Cost		Material	Mercedes GLC 350e Hybrid	Mercedes GLE 550e	Mitsubishi Outlander Plug In	Porsche Cayenne S E-Hybrid	Porsche Panamera S E-Hybrid	Toyota Prius PHEV	Volvo XC60 Plug In	Volvo-XC90 Plug In
		Type	PHEV	PHEV	PHEV	PHEV	PHEV	PHEV	PHEV	PHEV
		Capacity (kWh)	8.7	8.7	12	10.8	9.4	8.8	10.4	9.8
		Energy efficiency (Wh/mile)	590	690	450	700	650	450	550	550
		Chemistry	NMC622	NMC622	NMC622	NMC622	NMC622	NMC622	NMC622	NMC622
Materials ($/pack)	Positive active material	US EV fleet case	349	349	483	434	377	353	418	394
		US manufacturing scenario	468	468	648	582	506	474	561	528
		Ally-shoring scenario	433	433	599	538	468	438	518	488
		China	233	233	323	290	252	236	279	263
		Japan	485	485	670	602	524	490	580	546
		South Korea	332	332	459	413	359	336	397	374
		EU	414	414	572	514	447	418	495	466
		Negative active material	127	127	171	155	136	128	150	142
		Carbon black	7	7	10	9	8	7	8	8
		Positive current collector	15	15	14	14	14	14	14	14
		Negative current collector	64	64	60	62	63	64	62	63
		Separator	94	94	89	90	92	93	91	92
		Electrolyte	76	76	100	91	81	77	88	84
		Cell hardware	189	189	193	191	189	189	191	190
		Module hardware	730	730	741	737	732	730	736	733
		Battery jacket	333	333	343	340	336	333	338	336
		BMS	540	540	540	540	540	540	540	540
		Thermal management system	280	280	280	280	280	280	280	280
		Purchased items ($/pack)	1335	1335	1361	1352	1341	1335	1349	1344
Labor ($/pack)		US EV fleet case	330	330	301	374	368	291	185	183
		US manufacturing scenario	462	462	481	474	466	463	472	469
		Ally-shoring scenario	361	361	335	407	400	322	218	216
		China	166	166	177	173	168	166	172	170
		Japan	318	318	331	326	321	318	325	322
		South Korea	246	246	256	253	249	247	252	250
		EU	585	585	609	601	591	586	598	594
		US EV fleet case	719	719	770	758	750	750	713	710

Capital Equipment and Building ($/pack)	US manufacturing scenario	753	753	773	766	757	754	764	760
	Ally-shoring scenario	724	724	776	764	755	756	719	716
	China	707	707	725	719	711	707	716	713
	Japan	751	751	770	763	755	751	761	758
	South Korea	705	705	722	716	708	705	714	711
	EU	728	728	747	740	732	729	738	735

Table S23 Region-specific material cost of cathode active material production ($/kg), estimated using the trade value from the UN Comtrade database (*63*).

Region	$NiSO_4$	$CoSO_4$	MnO_2	$Al_2(SO4)_3$	Li_2CO_3	LiOH	Mn_2O_3
China	3.35	6.35	0.36	1.33	8.12	16.50	2.68
EU	3.80	25.41	2.03	0.25	11.08	7.97	0.75
Japan	3.45	43.61	2.38	0.22	13.18	12.91	2.18
South Korea	3.40	15.30	1.89	0.59	12.48	16.67	0.67
US	4.21	39.27	2.39	0.53	8.12	14.14	0.75

Table S24 Region-specific labor cost and building cost (*74-76*).

Region	Labor cost ($/hour)	Building cost ($/m^2)
Belgium	45.34	1452.21
Hungary	11.08	790.43
Poland	11.98	904.40
Germany	39.86	2034.57
Korea	16.78	1319.68
Japan	21.29	2720.93
US	31.46	2793.81
China	5.41	969.65
EU	31.91	1751.95

Table S25 Region-specific cathode active material cost ($/kg), estimated using the model from Ciez et al. (*81*).

Region	NMC622	NCA	NMC532	LMO/NMC532
China	18.01	20.29	17.47	13.93
EU	31.92	28.76	31.44	22.92
Japan	37.39	32.96	36.95	25.77
South Korea	25.61	24.97	25.19	18.72
US	36.14	34.95	35.60	24.73

In this section, we first focus on the US case and assess the key contributors and spatial variation in the automotive LIB's manufacturing costs based on the functional unit of 1 kWh battery energy capacity. We estimate that the manufacturing cost is $147 per kWh LIB for the 2019 US EV fleet, as presented in **Fig. S9a**. This estimation is close to the 2020 battery pack cost reported by the US Department of Energy (DOE) (*16, 84*). However, the manufacturing costs of LIBs vary across different EV models, as shown in **Fig. S8**.

The most influential factors to the manufacturing cost of automotive LIBs for the US case include the battery pack energy capacity and EV type. When LIB pack energy capacity is less than 19 kWh, the cost of purchased items takes the largest proportion of the manufacturing costs (**Fig. S9b**). Otherwise, material cost dominates LIB's manufacturing costs. This is primarily because the amount of purchased items for 1 kWh LIB is larger with lower energy capacity. Purchased items include terminals, cell container, module enclosure, battery jacket, battery management system, thermal management system, and other auxiliary hardware. The amount of purchased items is determined by several design parameters, including the number of cells per module, number of

cells and modules per battery pack, dimension of cells, modules and packs, energy capacity, and battery chemistry. While the number of cells per module and the number of cells and modules per battery pack are not accessible for most LIBs in different EV models, for a fair comparison, we adopt the default settings from the BatPac model and keep these parameters constant regardless of the energy capacity and battery chemistry of LIBs (*61*). The dimension of cells, modules, and packs are highly correlated with the energy capacity and battery chemistry of LIBs. EV type has an impact on the cell thickness as BEVs and PHEVs have different requirements for batteries. Specifically, the electrode thickness is determined by the sustained power requirement for BEVs and acceleration power requirement for PHEVs (*61*). In addition to the lower unit purchased item cost for high-capacity LIBs, the economies of scale for capital equipment, plant area, and working time are considered. Therefore, LIBs with greater energy capacity achieve lower unit manufacturing costs, as shown in **Fig. S9c**.

Material cost also takes advantage of volume discounts, but the benefits are minor, and the unit material cost remains almost constant with increasing energy capacity. In terms of the battery chemistry, LIBs with higher specific energy density tend to attain lower unit manufacturing costs, as shown in **Fig. S8**. However, due to the regional differences in producing cells and packs, manufacturing the high-performance NCA LIBs for BEV models of the US EV fleet gains less cost advantage over other types of LIBs. Despite the factors to raise the unit manufacturing cost of NCA LIBs, with a higher specific energy density, its unit manufacturing cost is still slightly lower than that of NMC622 LIBs.

The spatial variation in the EV battery value chain lies in the costs of cathode active materials, labor, construction, and transportation. These costs vary according to the production regions of LIB components, cells, and packs for each EV model. As cathode active materials are sourced from China, South Korea, Japan, the US, and the EU with respective shares (**Table S3**), spatial variation is not presented across different EV models. Among all the EV models, LIB cells and packs produced in South Korea, Poland, and Hungary achieve substantial cost benefits due to low labor costs. Only 1.6% of the LIB manufacturing cost can be attributed to transportation. However, the container shipping rates keep surging throughout the COVID-19 pandemic due to the lockdown measures and expanding electronic commerce. The sensitivity analysis result shows that the LIB manufacturing cost can be increased by up to 1.5% with a 166% higher container shipping rate (**Fig. S10**). The effect of spatial variation in building cost on LIB's manufacturing cost is subtle.

Fig. S10d shows that the LIB manufacturing cost of US manufacturing ($164/kWh) and ally-shoring ($155/kWh) scenario is 11% and 5% higher than the US EV fleet case ($147/kWh), respectively. The increase in the LIB manufacturing cost can be mainly attributed to the geographic variation in cathode active material cost and labor cost. Cathode active material cost and labor cost explain around 80% and 20% of the differences in the LIB manufacturing costs among these scenarios. As shown in **Table S3**, 42% of the LIB cathodes are produced in China, and the cathode active material cost in China is 21%–42% lower than that in South Korea, the EU, Japan, and the

US. The lower cathode active material cost can be mostly explained by the lower material, labor, and building cost in China. First, China is one of the largest producers of valuable metals used in LIBs, which accounts for 60%, 72%, and 16% of the world's production capacity for refined lithium, cobalt, and nickel, respectively (*17*). Therefore, there exist advantages in the material cost of refined lithium, cobalt, and nickel for local LIB manufacturers in China (*72*). Second, labor cost in China is 50%–88% lower than in the US, EU, South Korea, and Japan, as shown in **Table S19**. Third, as building cost is driven by labor cost (*72*), China has the lowest building cost among the LIB cathode production regions. **Fig. S10d** also indicates that the production of LIB components, cells, and packs exclusively in the Far East or the EU could be more cost-effective than in the US. Therefore, the increasing proportion of LIB component, cell, and pack production in the US, as shown in **Fig. S10a-c**, causes the worse economic performances of the US manufacturing and ally-shoring scenarios compared to the US EV fleet case. Soaring nickel price at up to $48/kg, two times higher than the pre-pandemic nickel price, can lead to a 16–21% increase in the EV battery manufacturing cost, which would delay the vehicle electrification due to demand-side challenges. When sourcing key critical minerals and materials domestically, the price of nickel exported from Canada to the US is used as a proxy for the domestic nickel price, which is lower than the nickel price exported from other countries, possibly due to lower transportation costs and higher nickel ore grade in Canada. Owing to the favorable nickel price and economic performance of lithium sourcing from geothermal brine, US's LIB products may gain cost advantages from domestic sourcing of critical materials.

To fairly allocate the climate change mitigation responsibilities, we calculate the border carbon adjustments required to achieve parity between LIBs produced in the US and their counterparts imported from the EU and main Asia suppliers. We compare these break-even border carbon adjustments with the carbon capture and storage costs reported in the existing literature and discuss the feasibility of avoiding carbon leakage.

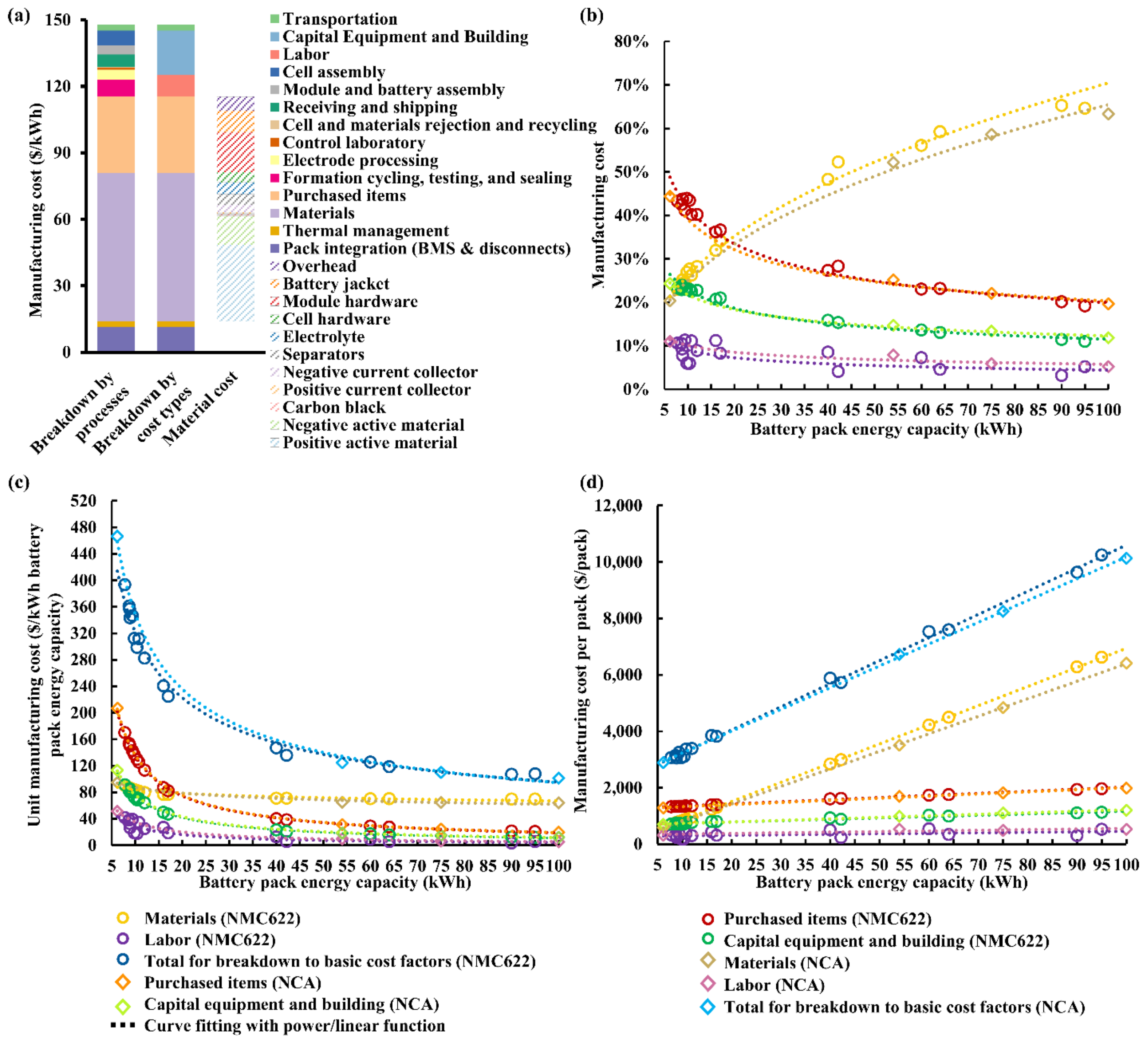

Fig. S9 Economic performance of LIBs for the pre-pandemic US EV fleet. a, Breakdowns of manufacturing cost by the manufacturing process and cost type. Breakdowns of material costs are also presented. **b**, Trends for shares of different types of LIB manufacturing costs with increasing battery pack energy capacity. NMC622 and NCA are chosen because they represent 98% of the US EV LIB market. **c**, Trends for different types of manufacturing costs per kWh energy capacity with increasing battery pack energy capacity. **d**, Trends for different types of manufacturing costs per pack with increasing battery pack energy capacity. Acronyms: battery management system (BMS).

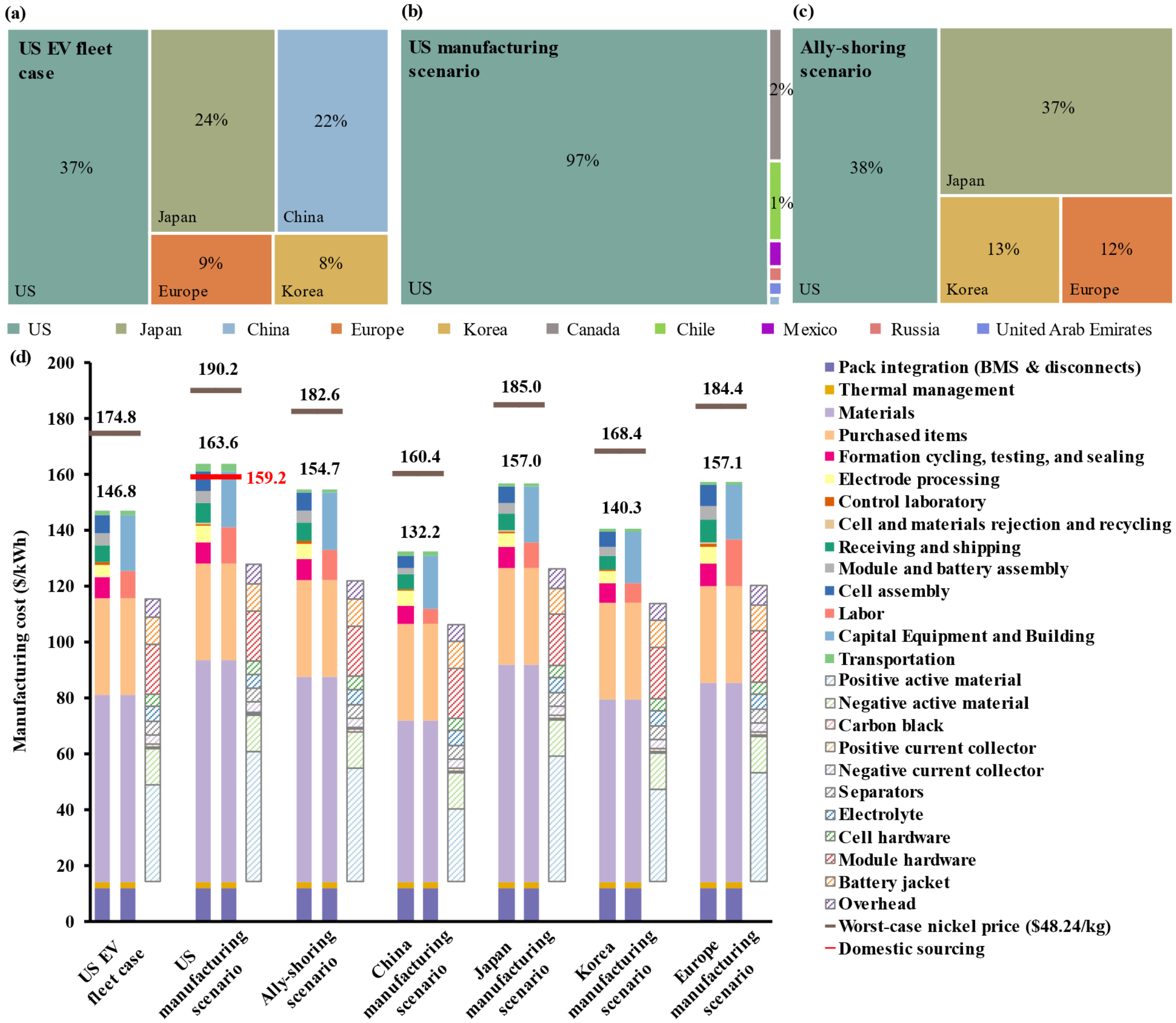

Fig. S10 Comparison of manufacturing costs across the US EV fleet case, US manufacturing scenario, and ally-shoring scenario. a, LIB manufacturing costs by the country for the US EV fleet case. **b**, LIB manufacturing costs by country for the US manufacturing scenario. **c**, LIB manufacturing costs by the country for the ally-shoring scenario. **d**, Comparison of LIB manufacturing costs across different scenarios, including US EV fleet case, US manufacturing scenario, ally-shoring scenario, and sensitivity analyses for manufacturing LIBs from components to packs in EU and main Asia suppliers. For **a-c**, the costs for all battery materials or battery assembly processes are aggregated by the production region. The sensitivity analysis results based on the record-high nickel price are presented as solid brown lines. Acronyms: battery management system (BMS).

Break-even border carbon adjustment analysis

A border carbon adjustment is proposed as a type of border tax adjustment to avoid carbon leakage and advance domestic competitiveness. In this study, we calculate the break-even border carbon adjustments for automotive LIBs imported from different regions to the US following the previous literature (*85*). In order to eliminate trade discrimination, the importing country should not charge border tax adjustments on the imported products in excess of taxes on domestic products, according to the WTO rules (*51*). In other words, if a border carbon adjustment is imposed on imported products, it should also be charged on domestic products in addition to the existing border carbon adjustments. Moreover, the border carbon adjustments priced in the exporting regions should be compensated by the importing country (*52*). Based on the above rules, the equation for calculating the break-even border carbon adjustments between the US and the importing regions, including the EU and the major LIB suppliers in the Far East, are revised as shown in equation (1), where *I* denotes the set of importing countries, indexed by *i*. These regions are considered as they are the largest LIB producers in the world (*16*). In equation (1), $BCA_{US,i}$ denotes the break-even border carbon adjustment between the US and region *i*, MC_{US} and MC_i represent the manufacturing cost of LIBs in the US and region *i*, respectively; CP_{US} and CP_i are the domestic carbon price in the US and the importing region *i*; GWP_{US} and GWP_i are the carbon footprint calculated for manufacturing EV LIBs in the US and the region *i*.

$$BCA_{US,i} = \frac{(MC_{US} + CP_{US} \times GWP_{US}) - (MC_i + CP_i \times GWP_i)}{GWP_i - GWP_{US}}, \forall i \in I \quad (1)$$

Note that there is no current domestic carbon tax in the US, China, and South Korea (*86*). Furthermore, to the best of our knowledge, Japan and European countries where main battery factories are located (i.e., Poland and Hungary) do not levy a carbon tax on EVs (*86-88*). However, Emissions Trading System (ETS) has been implemented in many countries as carbon pricing initiatives though none of them are targeting EV battery suppliers at this moment. Therefore, to investigate the potential implications of carbon pricing initiatives on the break-even border carbon adjustments, we conduct a sensitivity analysis on carbon pricing based on the World Bank statistics (*86, 89*). As shown in **Fig. S18**, 2019 and 2020 carbon prices and border carbon adjustments ranged \$0–\$127/t CO_2 eq. in the EU, \$1–\$12/t CO_2 eq. in China, \$8–\$17/t CO_2 eq. in the US, and \$22–\$33/t CO_2 eq. in South Korea, \$3–\$6/t CO_2 eq. in Japan (*86, 89*). The results are shown in **Fig. S19–SFig**. S21.

Border carbon adjustments are the charge levied on the embodied emissions of imports to secure competitive neutrality across countries with differential carbon prices and can be considered to facilitate mitigating climate change. According to World Trade Organization (WTO), in order to ensure competitive equality, countries cannot adjust the taxes on imported products in excess of domestic taxation (*51*). In other words, a country should impose the same border carbon adjustment on both its domestic products and imported products. To understand the trade-offs

between manufacturing costs and climate change of EV batteries, we calculate the break-even border carbon adjustments to be imposed on automotive LIBs consumed in the US, at which the summation of manufacturing cost and carbon price for LIBs manufactured domestically in the US and imported from other regions are equal. A baseline carbon price of \$0 per t CO_2 is considered as none of the assessed regions levies carbon taxes on EV or EV batteries at this moment. **Fig. S11a** shows that the break-even border carbon adjustment for automotive LIBs consumed in the US varies across the sources of the imported LIBs, EV type, and the battery chemistry. Specifically, among the largest LIB producers, the EU achieves negative break-even border carbon adjustments as LIB manufacturing in the EU is less cost-effective than in the US due to the lower embodied greenhouse gas emissions of LIBs from the EU suppliers. LIBs from their Asia suppliers are more carbon-intensive but more cost-effective than their US counterparts. The break-even border carbon adjustment between the US and China is the highest for all types of LIBs. A break-even border carbon adjustment at \$68 to \$1080 per t CO_2 on EV batteries imported from major Asia suppliers is reported. In particular, the break-even border carbon adjustments between the US and the LIB exporting countries in Asia range from \$65 to \$1051 per t CO_2 eq. for BEV LIBs and \$110 to \$1543 per t CO_2 eq. for PHEV LIBs. The differences in break-even border carbon adjustments by EV type can be attributed to the previously mentioned low battery pack energy density of PHEV LIBs and, consequently, more material and energy input to LIB manufacturing. The highest break-even border carbon adjustment is \$1735 per t CO_2 for NCA LIBs in PHEVs because there is only one PHEV model for NCA LIBs, and its battery pack energy capacity is the lowest among LIBs in all EV models. The geopolitical tensions over the supply and price of nickel do not significantly change the break-even border carbon adjustments. On the contrary, raising domestic sourcing of key critical minerals and materials, according to the US's recent Defense Production Act, can reduce the carbon footprint of domestic LIB products by 37%, thus lowering the break-even border carbon adjustments between the US and the LIB exporting Asian countries.

The break-even border carbon adjustments between the US and major Northeast Asian economies are within the range of carbon capture and storage costs in the existing studies, as presented in **Fig. S11c**. The carbon capture costs vary substantially by technology, industry, location, and the inclusion of carbon storage (*90-96*). Microsoft and Stripe proposed a biosphere-based carbon storage cost of \$16/t CO_2 eq. and a geosphere-based storage cost of \$141/t CO_2 eq. with a range of \$20–10,000/t CO_2 eq. (*97*). The results of many studies also supported that biosphere-based carbon storage costs less than geosphere-based storage (*90-95*).

It is worth mentioning that the scope of break-even analysis is limited to regulating carbon emissions of LIB manufacturing on the supply side, so it omits how the demand side would respond to the imposed border carbon adjustment as the cost would increase accordingly as shown in **Fig. S11b**. In detail, an increase in EV cost and consequently the price may stimulate the demand for conventional internal combustion engine vehicles and consequently deteriorate the efforts to reduce climate change impacts. Moreover, opponents of border carbon adjustment view it as a

protectionist and discriminatory policy measure to the developing countries that export cheaper but more carbon-intensive products (*52, 53*).

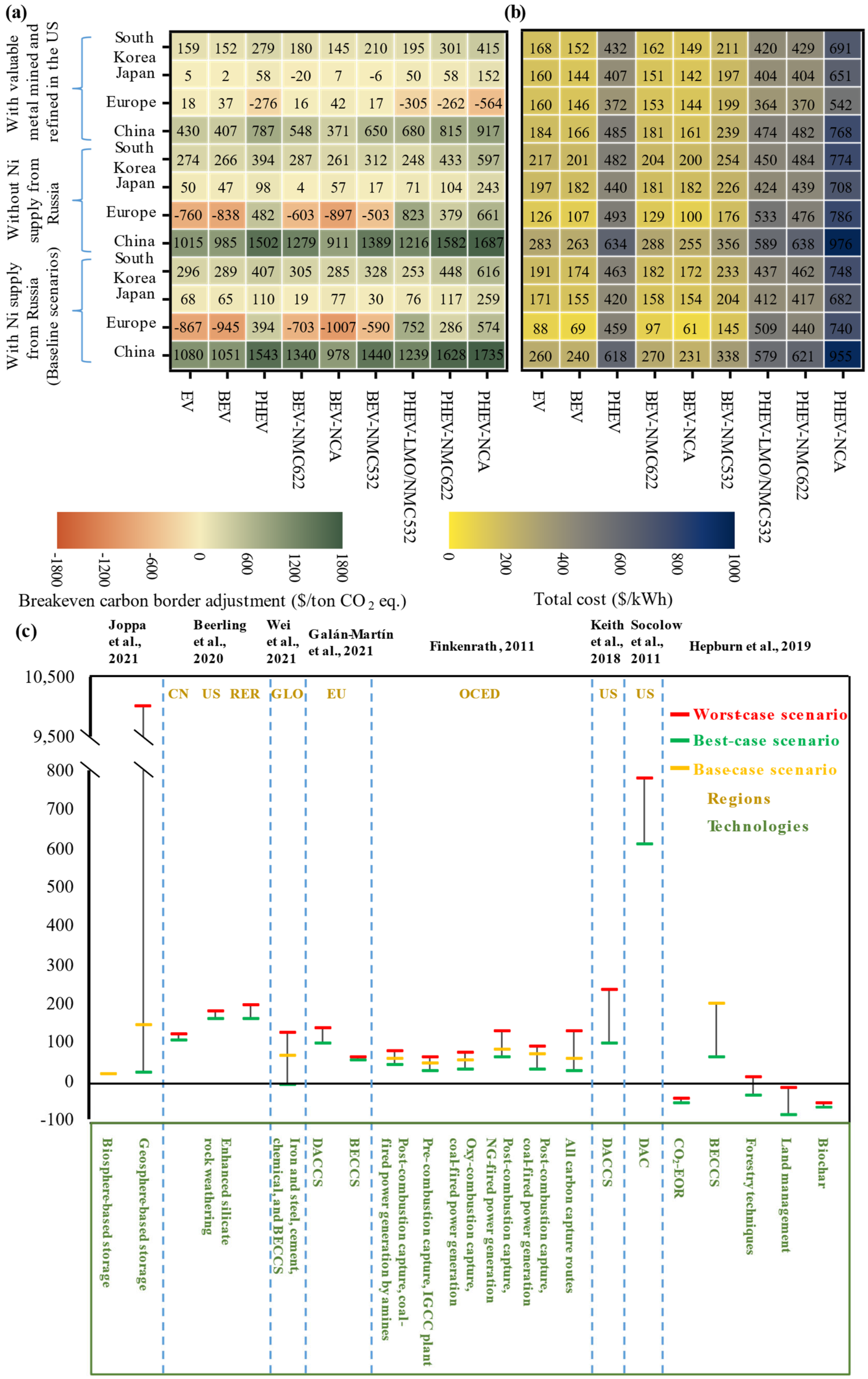
(a)
(b)
With valuable metal mined and refined in the US
Without Ni supply from Russia
With Ni supply from Russia (Baseline scenarios)
South Korea
Japan
Europe
China
159 152 279 180 145 210 195 301 415
5 2 58 -20 7 -6 50 58 152
18 37 -276 16 42 17 -305 -262 -564
430 407 787 548 371 650 680 815 917
274 266 394 287 261 312 248 433 597
50 47 98 4 57 17 71 104 243
-760 -838 482 -603 -897 -503 823 379 661
1015 985 1502 1279 911 1389 1216 1582 1687
296 289 407 305 285 328 253 448 616
68 65 110 19 77 30 76 117 259
-867 -945 394 -703 -1007 -590 752 286 574
1080 1051 1543 1340 978 1440 1239 1628 1735
168 152 432 162 149 211 420 429 691
160 144 407 151 142 197 404 404 651
160 146 372 153 144 199 364 370 542
184 166 485 181 161 239 474 482 768
217 201 482 204 200 254 450 484 774
197 182 440 181 182 226 424 439 708
126 107 493 129 100 176 533 476 786
283 263 634 288 255 356 589 638 976
191 174 463 182 172 233 437 462 748
171 155 420 158 154 204 412 417 682
88 69 459 97 61 145 509 440 740
260 240 618 270 231 338 579 621 955
EV
BEV
PHEV
BEV-NMC622
BEV-NCA
BEV-NMC532
PHEV-LMO/NMC532
PHEV-NMC622
PHEV-NCA
-1800 -1200 -600 0 600 1200 1800
Breakeven carbon border adjustment ($/ton CO2 eq.)
0 200 400 600 800 1000
Total cost ($/kWh)
(c)
Joppa et al., 2021
Beerling et al., 2020
Wei et al., 2021
Galán-Martín et al., 2021
Finkenrath, 2011
Keith et al., 2018
Socolow et al., 2011
Hepburn et al., 2019
CN
US
RER
GLO
EU
OCED
US
US
Worst-case scenario
Best-case scenario
Base-case scenario
Regions
Technologies
10,500
9,500
800
700
600
500
400
300
200
100
0
-100
Biosphere-based storage
Geosphere-based storage
Enhanced silicate rock weathering
Iron and steel, cement, chemical, and BECCS
DACCS
BECCS
Post-combustion capture, coal-fired power generation by amines
Pre-combustion capture, IGCC plant
Oxy-combustion capture, coal-fired power generation
Post-combustion capture, NG-fired power generation
Post-combustion capture, coal-fired power generation
All carbon capture routes
DACCS
DAC
CO2-EOR
BECCS
Forestry techniques
Land management
Biochar

Fig. S11 Comparison of break-even border carbon adjustments to carbon capture cost reported by previous studies. a, Break-even border carbon adjustments for the US on LIBs imported from major Northeast Asian economies and EU. **b**, Total cost of EV batteries considering the manufacturing cost and the break-even border carbon adjustment rate based on the baseline carbon price of \$0/t CO_2. **c**, Carbon capture costs reported by existing studies. Finkenrath did not include the carbon storage cost (*96*). Acronyms: China (CN); the United States (US); Europe (RER); European Union (EU); Organization for Economic Co-operation and Development (OECD).

Sensitivity analysis

In this section, sensitivity analysis results on the LIB manufacturing cost, carbon footprint, cumulative energy demand (CED), mineral resource scarcity, and break-even border carbon adjustment are provided. Sensitivity analysis of the container shipping rate shows that LIB manufacturing cost can be increased by up to 1.5% with a 166% higher container shipping rate (**Fig. S12**). Sensitivity analysis of temporal variation in the power grid implies that the clean energy transitions from 2019 to 2050 can reduce the carbon footprint by up to 5% and CED by up to 3% for the US manufacturing scenario, as shown in **Fig. S13–SFig**. S14. For the ally-shoring scenario, the reduction in carbon footprint and CED are up to 7% and 4%, respectively. The sensitivity analysis result of spatial variation in the power grid suggests that there will be up to 11% and 7% reduction in the carbon footprint and CED if the LIB is produced in the region with the least carbon- and energy-intensive power grid. The result suggests that relocating the midstream and downstream automotive LIB value chain in the Northeast Power Coordinating Council (NPCC) and Western Electricity Coordinating Council (WECC) region of the US will be the least carbon- and energy-intensive by 2050, respectively. On the contrary, the power grid in the Reliability First Corporation (RFC) region of the US will be the most carbon- and energy-intensive in 2050. **Fig. S15-SFig**. S17 demonstrate the sensitivity analysis results of the recycled content for valuable metals on the carbon footprint, CED, and mineral resource scarcity of the US EV fleet case, US manufacturing scenario, and ally-shoring scenario. For the US EV fleet, using 100% secondary nickel would reduce the carbon footprint by 26–37%, CED by 23–33%, and mineral resource scarcity by 27–43%, suggesting a greater emission reduction benefit compared to adopting 100% recycled cobalt, aluminum, copper, or steel. Among the three scenarios, the US manufacturing scenario achieves the most reduction benefits in the carbon footprint and CED, while the ally-shoring scenario can mitigate the most surplus ore potential by adopting 100% secondary nickel. By adopting 100% secondary aluminum for manufacturing automotive LIBs, both the US EV fleet case and ally-shoring scenario have the potential to achieve around 10% reduction in the carbon footprint and CED. **Fig. S18** depicts the worst- and best-case carbon price for the sensitivity analysis on break-even border carbon adjustments. The results (**Fig. S19–SFig**. S21) imply limited impacts of variation in existing carbon price on the break-even carbon border adjustment, compared to the more influential battery pack energy capacity, battery chemistry, and production regions. **Table S26** provides the material cost of cathode active material production for sensitivity

analysis on nickel supply from Russia and domestic sourcing of critical battery materials. The record-high nickel price during the recent Russia-Ukraine war is adopted as the worst case for the US manufacturing and ally-shoring scenarios without nickel supply from Russia. For the domestic sourcing of critical battery materials, we consider the unit prices of $NiSO_4$ and $CoSO_4$ exported from Canada to the US as proxies for the prices of domestic originated metals; in terms of the lithium price, we calculate the weighted sum of the lithium price exported from Canada to the US and the projected lithium price considering future demand growth and potential supply from geothermal brine given the respective production capacities of lithium extraction from conventional and geothermal brines.

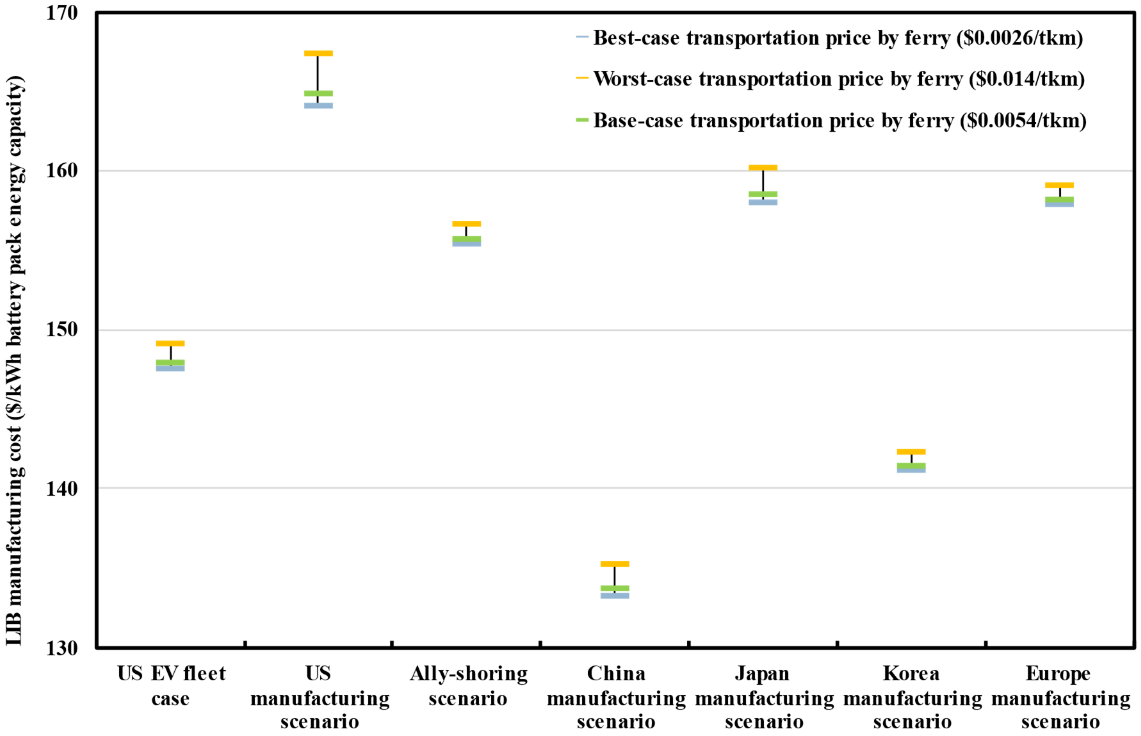

Fig. S12 Sensitivity analysis of container shipping rate. LIB manufacturing costs can be increased by up to 1.5% with a 166% higher container shipping rate.

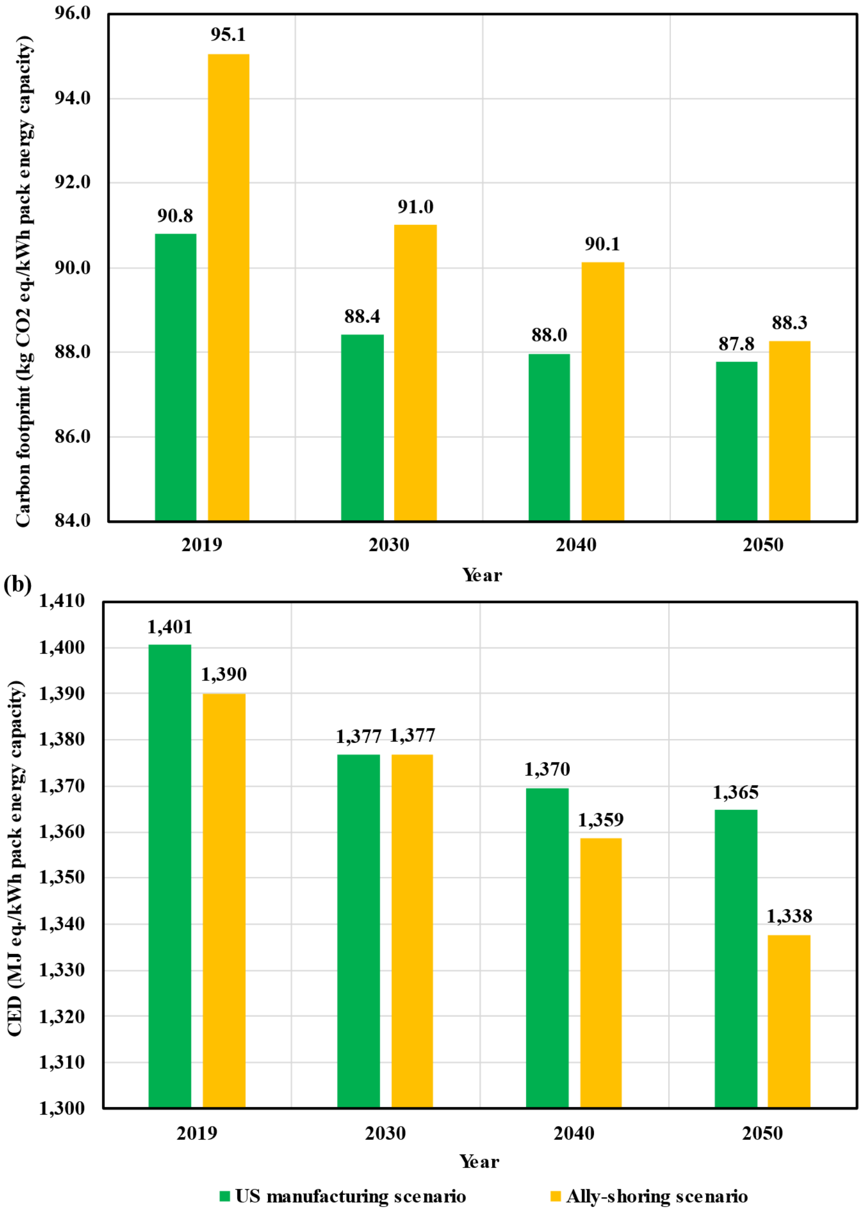

Fig. S13 Sensitivity analysis of temporal variation in the power grid for the US manufacturing and ally-shoring scenarios. a, carbon footprint with projected power grids in 2030, 2040, and 2050. **b**, CED with projected power grids in 2030, 2040, and 2050. The result shows that both the carbon footprint and CED are reduced gradually from 2019 to 2050. For the US manufacturing scenario, the reduction in carbon footprint varies from 12.1% for an average EV battery manufactured in 2019 to 15.0% for its counterpart manufactured in 2050. Similarly, the reduction in CED varies from 2.9% to 5.4%. Moreover, for the ally-shoring scenario, the reduction in carbon footprint varies from 7.9% for an average EV battery manufactured in 2019 to 14.5% for its counterpart manufactured in 2050. Similarly, the reduction in CED varies from 3.6%

to 7.2%. Compared to the US manufacturing scenario, the ally-shoring scenario achieves more reduction in both carbon footprint and CED from varying the power grid from 2019 to 2050.

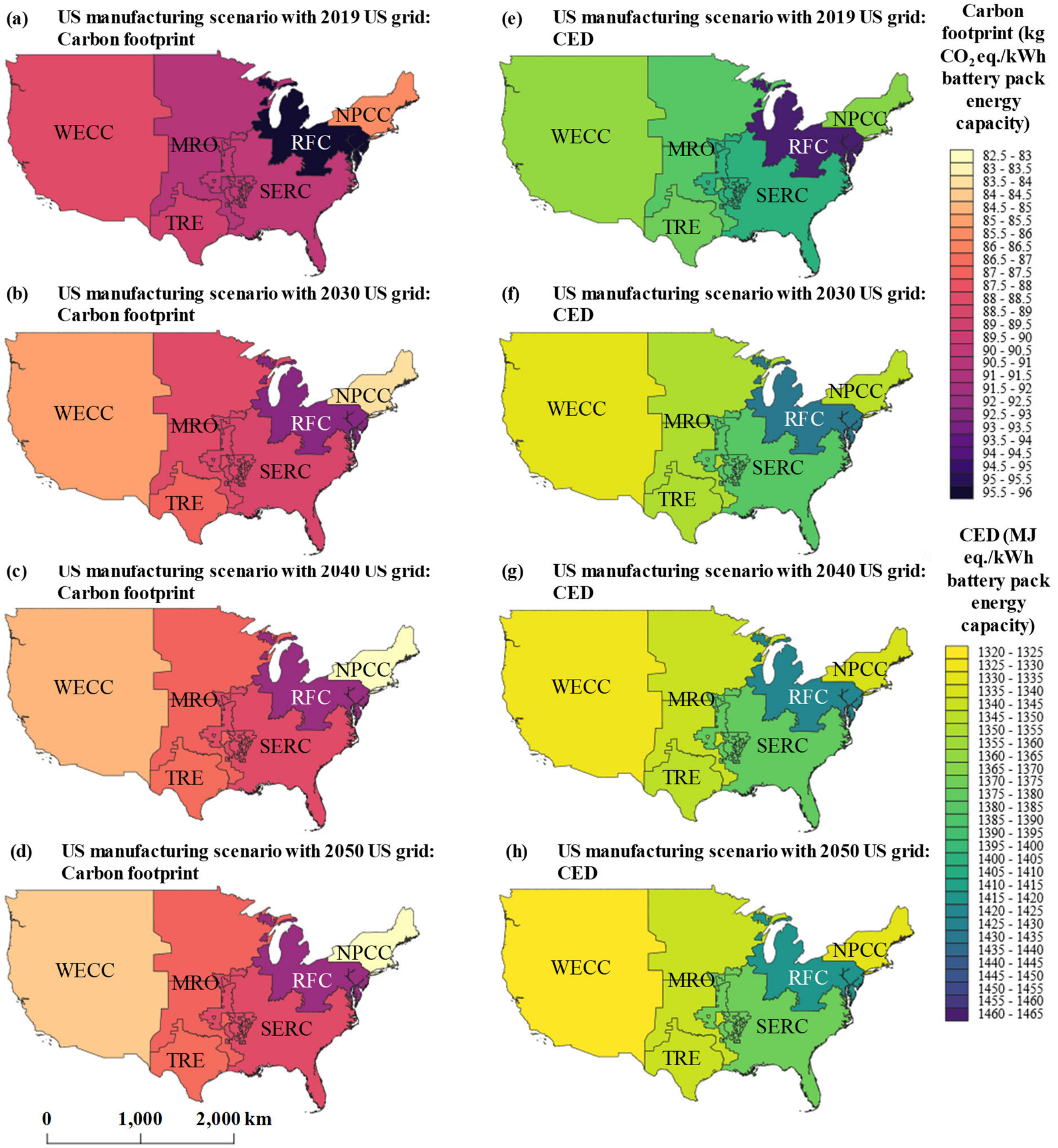

Fig. S14 Sensitivity analysis of spatial and temporal variation in the power grid for the US manufacturing scenarios. a, **b**, and **c**, represent the carbon footprint of the US manufacturing scenario with the projected power grid in 2030, 2040, and 2050, respectively. **d**, **e**, and **f**, represent the CED of the US manufacturing scenario with the projected power grid in 2030, 2040, and 2050, respectively. Acronyms: Midwest Reliability Organization (MRO), Texas Reliability Entity (TRE), Southeast Regional Council (SERC).

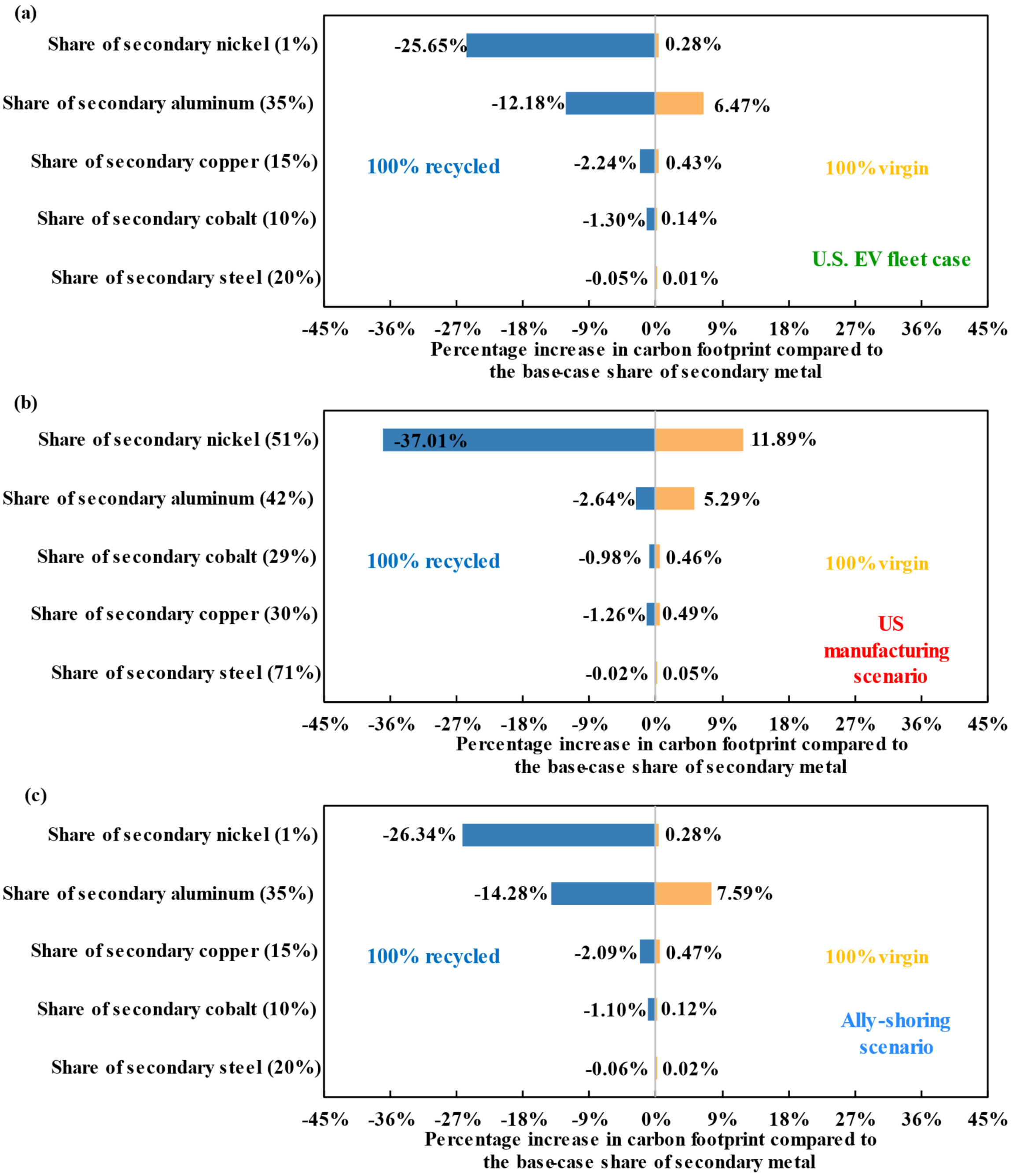

Fig. S15 Sensitivity analysis of carbon footprint on secondary metal supply. a, US EV fleet case. **b**, US manufacturing scenario. **c**, Ally-shoring scenario.

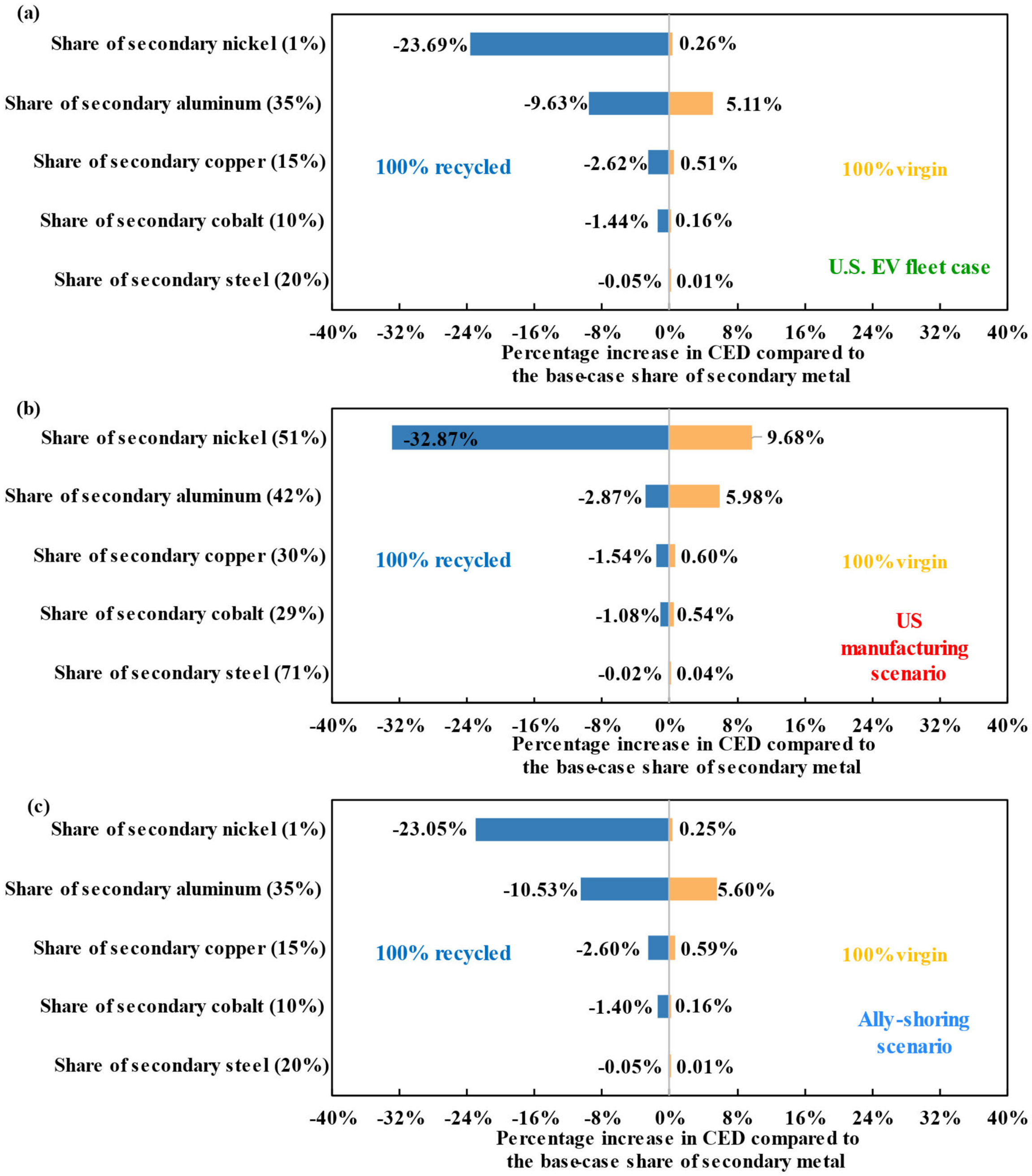

Fig. S16 Sensitivity analysis of CED on secondary metal supply. a, US EV fleet case. **b**, US manufacturing. **c**, Ally-shoring scenario.

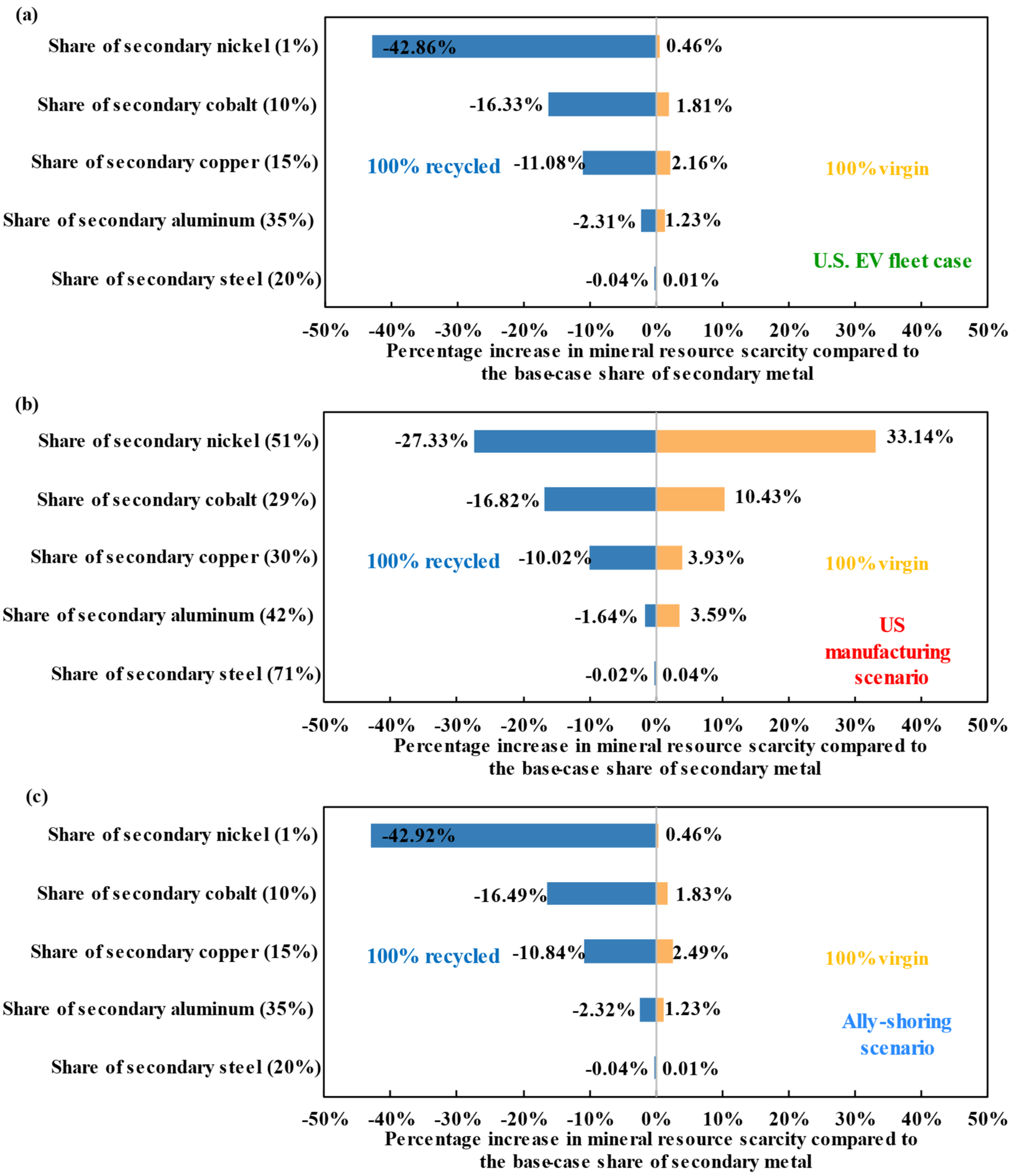

Fig. S17 Sensitivity analysis of mineral resource scarcity on secondary metal supply. a, US EV fleet case. **b**, US manufacturing scenario. **c**, Ally-shoring scenario.

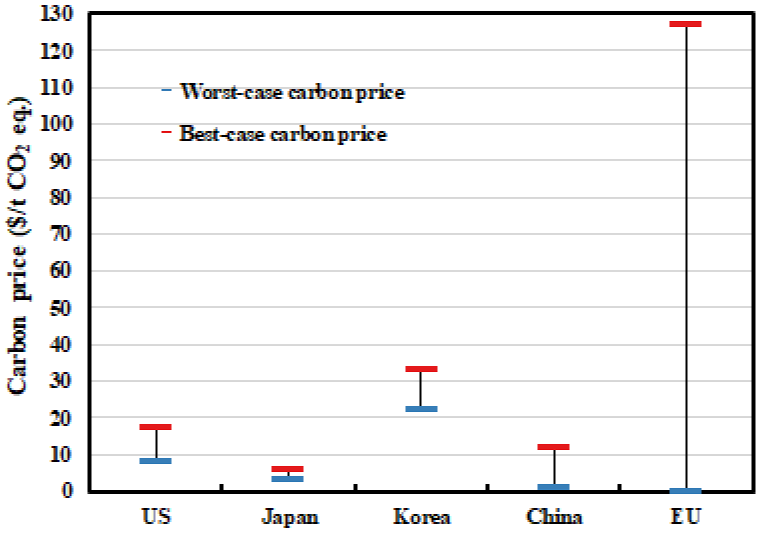

Fig. S18 Worst- and best-case parameters for the sensitivity analysis on the carbon price.

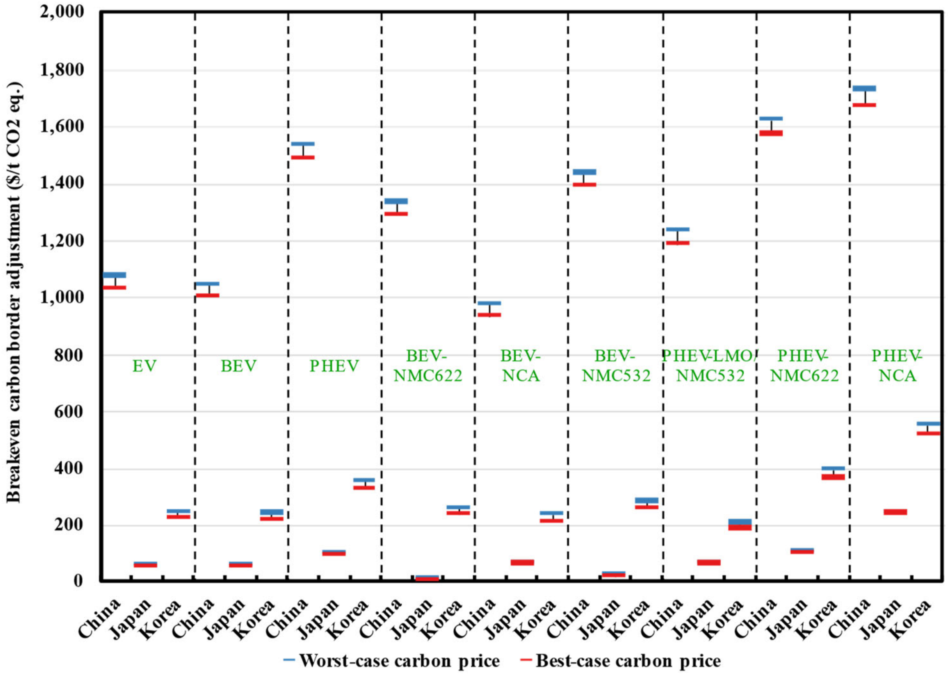

Fig. S19 Sensitivity analysis of the carbon price of the East Asia countries when the base-case US carbon price of $0/t CO_2 eq.

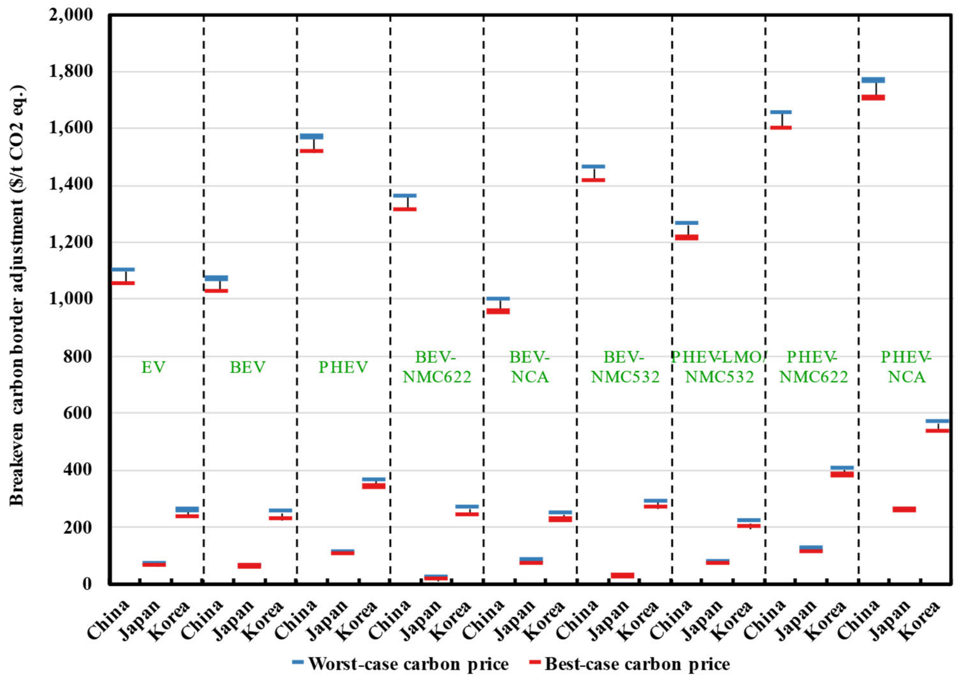

Fig. S20 Sensitivity analysis of the carbon price of the East Asia countries when the worst-case US carbon price of $8/t CO_2 eq.

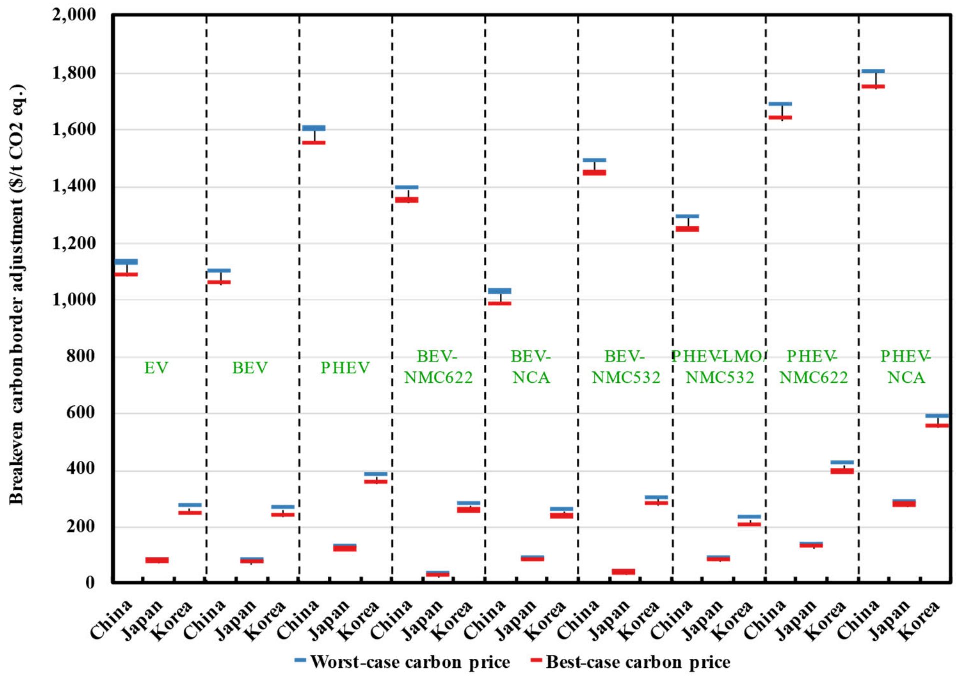

Fig. S21 Sensitivity analysis of the carbon price of the East Asia countries when the best-case US carbon price of $17/t CO_2 eq.

Table S26 Material cost of cathode active material production ($/kg) for sensitivity analysis on nickel supply from Russia and domestic sourcing of critical battery materials (*63, 66, 80*).

Parameter	$NiSO_4$	$CoSO_4$	Li_2CO_3	LiOH
Domestic sourcing of critical battery materials	1.63	40.98	7.94	11.58
Nickel supply from Russia	18.36	**	**	**

Table S27 Total cost of EV batteries considering the manufacturing cost and the break-even border carbon adjustment rate based on the baseline carbon price of $0/t CO_2.

Sensitivity analysis	Item	Country where the US imported LIBs from	EV	BEV	PHEV	EV-NMC622	EV-NCA	EV-NMC532	PHEV-LMO/NMC532	PHEV-NMC622	PHEV-NCA
With Ni supply from Russia (Baseline scenario)	Break-even border carbon adjustment ($/t CO_2)	China	1080	1051	1543	1340	978	1440	1239	1628	1735
		Europe	-867	-945	394	-703	-1007	-590	752	286	574
		Japan	68	65	110	19	77	30	76	117	259
		Korea	296	289	407	305	285	328	253	448	616
	Manufacturing cost ($/kWh)	China	133	119	346	120	119	157	346	341	558
		Europe	159	143	408	152	141	197	406	404	640
		Japan	159	144	393	155	141	199	389	390	608
		Korea	142	128	364	133	126	173	363	360	575
	Total cost ($/kWh)	China	260	240	618	270	231	338	579	621	955
		Europe	88	69	459	97	61	145	509	440	740
		Japan	171	155	420	158	154	204	412	417	682
		Korea	191	174	463	182	172	233	437	462	748
Without Ni supply from Russia	Break-even border carbon adjustment ($/t CO_2)	China	1015	985	1502	1279	911	1389	1216	1582	1687
		Europe	-760	-838	482	-603	-897	-503	823	379	661
		Japan	50	47	98	4	57	17	71	104	243
		Korea	274	266	394	287	261	312	248	433	597
	Manufacturing cost ($/kWh)	China	163	150	369	145	150	181	361	367	590
		Europe	188	173	430	177	171	221	420	428	670
		Japan	189	174	416	180	172	222	403	415	639
		Korea	172	158	387	158	158	197	377	386	606
	Total cost ($/kWh)	China	283	263	634	288	255	356	589	638	976
		Europe	126	107	493	129	100	176	533	476	786
		Japan	197	182	440	181	182	226	424	439	708
		Korea	217	201	482	204	200	254	450	484	774
With valuable metal mined and refined in the US	Break-even border carbon adjustment ($/t CO_2)	China	430	407	787	548	371	650	680	815	917
		Europe	18	37	-276	16	42	17	-305	-262	-564
		Japan	5	2	58	-20	7	-6	50	58	152
		Korea	159	152	279	180	145	210	195	301	415
	Manufacturing cost ($/kWh)	China	133	119	346	120	119	157	346	341	558
		Europe	159	143	408	152	141	197	406	404	640
		Japan	159	144	393	155	141	199	389	390	608
		Korea	142	128	364	133	126	173	363	360	575
	Total cost ($/kWh)	China	184	166	485	181	161	239	474	482	768
		Europe	160	146	372	153	144	199	364	370	542
		Japan	160	144	407	151	142	197	404	404	651
		Korea	168	152	432	162	149	211	420	429	691

** Unchanged values can be found in Table S5

Table S28 Abbreviations in the figure legends of main text.

Abbreviation	Definition
BMS	battery management system
CED	cumulative energy demand
DMC	dimethyl carbonate
EC	ethylene carbonate
EOFP	ozone formation (terrestrial ecosystems)
FEP	freshwater eutrophication
FETP	freshwater ecotoxicity
FFP	fossil resource scarcity
GWP	global warming potential
HOFP	ozone formation (human health)
HTPc	human carcinogenic toxicity
HTPnc	human non-carcinogenic toxicity
IRP	ionizing radiation
LOP	land use
MEP	marine eutrophication
METP	marine ecotoxicity
NMP	N-methyl-2-pyrrolidone
ODP	stratospheric ozone depletion
PMFP	fine particulate matter formation
PVDF	polyvinylfluoride
SOP	mineral resource scarcity
TAP	terrestrial acidification
TETP	terrestrial ecotoxicity
WCP	water consumption

REFERENCES AND NOTES

1. Greenhouse Gas Emissions from Energy: Overview (International Energy Agency (IEA), 2021); https://www.iea.org/data-and-statistics/data-product/greenhouse-gas-emissions-from-energy-highlights [accessed 17 January 2022].

2. P. Wolfram, S. Weber, K. Gillingham, E. G. Hertwich, Pricing indirect emissions accelerates low—Carbon transition of US light vehicle sector. *Nat. Commun.* **12**, 7121 (2021).

3. Global EV Outlook 2021 - Trends and developments in electric vehicle markets (International Energy Agency, 2021); https://www.iea.org/reports/global-ev-outlook-2021/trends-and-developments-in-electric-vehicle-markets [accessed 03 June 2021].

4. A. Milovanoff, I. D. Posen, H. L. MacLean, Electrification of light-duty vehicle fleet alone will not meet mitigation targets. *Nat. Clim. Chang.* **10**, 1102–1107 (2020).

5. R. Schmuch, R. Wagner, G. Hörpel, T. Placke, M. Winter, Performance and cost of materials for lithium-based rechargeable automotive batteries. *Nat. Energy* **3**, 267–278 (2018).

6. C. Zhang, X. Zhao, R. Sacchi, F. You, Trade-off between critical metal requirement and transportation decarbonization in automotive electrification. *Nat. Commun.* **14**, 1616 (2023).

7. T. R. Hawkins, B. Singh, G. Majeau-Bettez, A. H. Strømman, Comparative environmental life cycle assessment of conventional and electric vehicles. *J. Ind. Ecol.* **17**, 53–64 (2013).

8. D. A. Notter, M. Gauch, R. Widmer, P. Wäger, A. Stamp, R. Zah, H.-J. Althaus, Contribution of Li-Ion batteries to the environmental impact of electric vehicles. *Environ. Sci. Technol.* **44**, 6550–6556 (2010).

9. Z. Wu, M. Wang, J. Zheng, X. Sun, M. Zhao, X. Wang, Life cycle greenhouse gas emission reduction potential of battery electric vehicle. *J. Clean. Prod.* **190**, 462–470 (2018).

10. L. A.-W. Ellingsen, B. Singh, A. H. Strømman, The size and range effect: Lifecycle greenhouse gas emissions of electric vehicles. *Environ. Res. Lett.* **11**, 054010 (2016).

11. J. Baars, T. Domenech, R. Bleischwitz, H. E. Melin, O. Heidrich, Circular economy strategies for electric vehicle batteries reduce reliance on raw materials. *Nat. Sustain.* **4**, 71–79 (2021).

12. A. Zeng, W. Chen, K. D. Rasmussen, X. Zhu, M. Lundhaug, D. B. Müller, J. Tan, J. K. Keiding, L. Liu, T. Dai, A. Wang, G. Liu, Battery technology and recycling alone will not save the electric mobility transition from future cobalt shortages. *Nat. Commun.* **13**, 1341 (2022).

13. W. Li, E. M. Erickson, A. Manthiram, High-nickel layered oxide cathodes for lithium-based automotive batteries. *Nat. Energy* **5**, 26–34 (2020).

14. A. Ata, S. Zhi, P. Sandeep, COVID-19 disruptions to tech-metals supply are a wake-up call. *Nature* **587**, 365–367 (2020).

15. S. Althaf, C. W. Babbitt, Disruption risks to material supply chains in the electronics sector. *Resour. Conserv. Recycl.* **167**, 105248 (2021).

16. National Blueprint for Lithium Batteries 2021–2030 Executive Summary [U.S. Department of Energy (DOE), 2021]; https://www.energy.gov/sites/default/files/2021-06/FCAB%20National%20Blueprint%20Lithium%20Batteries%200621_0.pdf [accessed 15 January 2022].

17. Building resilient supply chains, revitalizing American manufacturing, and fostering broad-based growth (The White House, 2021); https://www.whitehouse.gov/wp-content/uploads/2021/06/100-day-supply-chain-review-report.pdf.

18. J. C. Kelly, Q. Dai, M. Wang, Globally regional life cycle analysis of automotive lithium-ion nickel manganese cobalt batteries. *Mitig. Adapt. Strat. Glob. Chang.* **25**, 371–396 (2020).

19. Z. Yang, H. Huang, F. Lin, Sustainable electric vehicle batteries for a sustainable world: Perspectives on battery cathodes, environment, supply chain, manufacturing, life cycle, and policy. *Adv. Energy Mater.* **12**, 2200383 (2022).

20. N. O. Bonsu, Towards a circular and low-carbon economy: Insights from the transitioning to electric vehicles and net zero economy. *J. Clean. Prod.* **256**, 120659 (2020).

21. Mineral commodity summaries 2021 [U.S. Geological Survey (USGS), 2021]; https://pubs.er.usgs.gov/publication/mcs2021 [accessed 17 January 2022].

22. Battery Critical Materials Supply Chain Opportunities (U.S. Department of Energy, Office of Energy Efficiency & Renewable Energy, 2020); https://www.energy.gov/eere/amo/articles/battery-critical-materials-supply-chain-challenges-and-opportunities-results-0 [accessed 03 February 2022].

23. Y. Tao, D. Rahn Christopher, A. Archer Lynden, F. You, Second life and recycling: Energy and environmental sustainability perspectives for high-performance lithium-ion batteries. *Sci. Adv.* **7**, eabi7633 (2021).

24. M. Hiremath, K. Derendorf, T. Vogt, Comparative life cycle assessment of battery storage systems for stationary applications. *Environ. Sci. Technol.* **49**, 4825–4833 (2015).

25. G. Majeau-Bettez, T. R. Hawkins, A. H. Strømman, Life cycle environmental assessment of lithium-ion and nickel metal hydride batteries for plug-in hybrid and battery electric vehicles. *Environ. Sci. Technol.* **45**, 4548–4554 (2011).

26. R. Frischknecht, LCI modelling approaches applied on recycling of materials in view of environmental sustainability, risk perception and eco-efficiency. *Int. J. Life Cycle Assess.* **15**, 666–671 (2010).

27. R. Sommerville, P. Zhu, M. A. Rajaeifar, O. Heidrich, V. Goodship, E. Kendrick, A qualitative assessment of lithium ion battery recycling processes. *Resour. Conserv. Recycl.* **165**, 105219 (2021).

28. Inventory of U.S. greenhouse gas emissions and sinks [U.S. Environmental Protection Agency (EPA), 2021]; https://www.epa.gov/ghgemissions/inventory-us-greenhouse-gas-emissions-and-

sinks#:~:text=Key%20findings%20from%20the%201990,sequestration%20from%20the%20land%20sector [accessed 15 January 2022].

29. U.S. energy facts explained [U.S. Energy Information Administration (EIA), 2021]; https://www.eia.gov/energyexplained/us-energy-facts/#:~:text=In%202021%2C%20production%20equaled%2097.78,primary%20energy%20production%20in%202021 [accessed 17 January 2022].

30. Emissions & generation resource integrated database (United States Environmental Protection Agency); https://www.epa.gov/egrid [accessed 22 January 2021].

31. World energy statistics and balances [International Energy Agency (IEA), 2021]; https://www.iea.org/data-and-statistics/data-product/world-energy-statistics-and-balances [accessed 15 January 2022].

32. Q. Dai, J. C. Kelly, L. Gaines, M. Wang, Life cycle analysis of lithium-ion batteries for automotive applications. *Batteries* **5**, 48 (2019).

33. T.-Y. Huang, J. R. Pérez-Cardona, F. Zhao, J. W. Sutherland, M. P. Paranthaman, Life cycle assessment and techno-economic assessment of lithium recovery from geothermal brine. *ACS Sustain. Chem. Eng.* **9**, 6551–6560 (2021).

34. The Role of Critical Minerals in Clean Energy Transitions [International Energy Agency (IEA), 2021]; https://www.iea.org/reports/the-role-of-critical-minerals-in-clean-energy-transitions [accessed 17 January 2022].

35. G. Wernet, C. Bauer, B. Steubing, J. Reinhard, E. Moreno-Ruiz, B. Weidema, The ecoinvent database version 3 (part I): Overview and methodology. *Int. J. Life Cycle Assess.* **21**, 1218–1230 (2016).

36. Aluminium Recycling [International Aluminium Institute (IAI), 2020]; https://recycling.world-aluminium.org/home/ [accessed 15 January 2022].

37. Aluminium [International Energy Agency (IEA), 2021]; https://www.iea.org/reports/aluminium [accessed 15 January 2022].

38. B. Christian, Lignite mine operation, allocation, cut-off by classification (ecoinvent database version 3.8); https://ecoinvent.org/the-ecoinvent-database/data-releases/ecoinvent-3-8/ [accessed 15 January 2022].

39. T. Karin, Market for electricity, high voltage, allocation, cut-off by classification (ecoinvent database version 3.8); https://ecoinvent.org/the-ecoinvent-database/data-releases/ecoinvent-3-8/ [accessed 15 January 2022].

40. B. Guillaume, Market for aluminium, wrought alloy, GLO, Allocation, cut-off by classification (ecoinvent database version 3.8); https://ecoinvent.org/the-ecoinvent-database/data-releases/ecoinvent-3-8/ [accessed 15 January 2022].

41. Aluminum Statistics and Information [U.S. Geological Survey (USGS), 2021]; https://www.usgs.gov/centers/national-minerals-information-center/aluminum-statistics-and-information [accessed 15 January 2022].

42. W. Linlin, Aluminium ingot, primary, to aluminium, wrought alloy market, GLO, Allocation, cut-off by classification (ecoinvent database version 3.8); https://ecoinvent.org/the-ecoinvent-database/data-releases/ecoinvent-3-8/ [accessed 15 January 2022].

43. G. M. Mudd, Global trends and environmental issues in nickel mining: Sulfides versus laterites. *Ore Geol. Rev.* **38**, 9–26 (2010).

44. Y. Deng, J. Li, T. Li, X. Gao, C. Yuan, Life cycle assessment of lithium sulfur battery for electric vehicles. *J. Power Sources* **343**, 284–295 (2017).

45. F. Wang, Y. Deng, C. Yuan, Life cycle assessment of lithium oxygen battery for electric vehicles. *J. Clean. Prod.* **264**, 121339 (2020).

46. F. Duffner, N. Kronemeyer, J. Tübke, J. Leker, M. Winter, R. Schmuch, Post-lithium-ion battery cell production and its compatibility with lithium-ion cell production infrastructure. *Nat. Energy* **6**, 123–134 (2021).

47. R. Tom, A Tesla Co-Founder Aims To Build an Entire U.S. Battery Industry (Bloomberg, 2021); https://www.bloomberg.com/news/articles/2021-09-14/a-tesla-co-founder-aims-to-build-an-entire-u-s-battery-industry#xj4y7vzkg [accessed 15 January 2022].

48. D. Paulikas, S. Katona, E. Ilves, S. H. Ali, Life cycle climate change impacts of producing battery metals from land ores versus deep-sea polymetallic nodules. *J. Clean. Prod.* **275**, 123822 (2020).

49. Science & Tech Spotlight: Deep-sea Mining [U.S. Government Accountability Office (GAO), 2021]; https://www.gao.gov/products/gao-22-105507 .

50. Governments must weigh the environmental costs of deep-sea mining. *Nature* **559**, 152 (2018).

51. Taxes and Charges for Environmental Purposes - Border Tax Adjustment (Note by the Secretariat) [World Trade Organization (WTO), 1997]; https://docs.wto.org/dol2fe/Pages/FE_Search/FE_S_S009-DP.aspx?language=E&CatalogueIdList=6608&CurrentCatalogueIdIndex=0&FullTextHash=&HasEnglishRecord=True&HasFrenchRecord=True&HasSpanishRecord=True.

52. C. Böhringer, C. Fischer, K. E. Rosendahl, T. F. Rutherford, Potential impacts and challenges of border carbon adjustments. *Nat. Clim. Chang.* **12**, 22–29 (2022).

53. G. A. Goldthau, L. Hughes, Protect global supply chains for low-carbon technologies. *Nature* **585**, 28–30 (2020).

54. M. Wang, X. Mao, Y. Xing, J. Lu, P. Song, Z. Liu, Z. Guo, K. Tu, E. Zusman, Breaking down barriers on PV trade will facilitate global carbon mitigation. *Nat. Commun.* **12**, 6820 (2021).

55. H. Liang, F. You, Reshoring silicon photovoltaics manufacturing contributes to decarbonization and climate change mitigation. *Nat. Commun.* **14**, 1274 (2023).

56. ISO 14040:2006 Environmental management — Life cycle assessment — Principles and framework (International Standards Organization, 2006); https://www.iso.org/standard/37456.html.

57. U.S. Plug-in Electric Vehicles Sales by Model (Transportation Research Center at Argonne National Laboratory, 2020); https://afdc.energy.gov/data [accessed 17 January 2022].

58. D. Gohlke, Y. Zhou, Assessment of Light-Duty Plug-in Electric Vehicles in the United States, 2010–2020 [Argonne National Laboratory (ANL), 2021]; https://publications.anl.gov/anlpubs/2021/06/167626.pdf [accessed 17 January 2022].

59. fueleconomy.gov, [U.S. Department of Energy (DOE) and the U.S. Environmental Protection Agency (EPA), 2021] [accessed 17 January 2022].

60. Y. Zhou, D. Gohlke, L. Rush, J. Kelly, Q. Dai, "Lithium-Ion battery supply chain for e-drive vehicles in the United States: 2010–2020" (2021, https://www.osti.gov/servlets/purl/1778934).

61. P. A. Nelson, S. Ahmed, K. G. Gallagher, D. W. Dees, Modeling the Performance and Cost of Lithium-Ion Batteries for Electric-Drive Vehicles, Third Edition (Office of Scientific and Technical Information, 2019); https://www.osti.gov/biblio/1503280.

62. K. Richa, C. W. Babbitt, N. G. Nenadic, G. Gaustad, Environmental trade-offs across cascading lithium-ion battery life cycles. *Int. J. Life Cycle Assess.* **22**, 66–81 (2017).

63. UN Comtrade Database [United Nations (UN)]; https://comtradeplus.un.org/ [accessed 15 January 2022].

64. “State of the Cobalt market” report (Cobalt Institute, 2021); https://www.cobaltinstitute.org/wp-

content/uploads/2021/05/CobaltInstitute_Market_Report_2020_1.pdf [accessed 17 January 2022].

65. A. Manthiram, X. Yu, S. Wang, Lithium battery chemistries enabled by solid-state electrolytes. *Nat. Rev. Mater.* **2**, 16103 (2017).

66. G. E. Blomgren, The development and future of lithium ion batteries. *J. Electrochem. Soc.* **164**, A5019–A5025 (2017).

67. J. P. Park, Electrical Components KR EV Battery 2020: Momentum to rebuild (CGSCIMB, 2019); https://www.skc.co.kr/upload/ir/20191216/20191216134740943289.pdf [accessed 17 January 2022].

68. Research Study on Reuse and Recycling of Batteries Employed in Electric Vehicles: The Technical, Environmental, Economic, Energy and Cost Implications of Reusing and Recycling EV Batteries (Kelleher Environmental, 2019); https://www.api.org/~/media/files/oil-and-natural-gas/fuels/kelleher%20final%20ev%20battery%20reuse%20and%20recycling%20report%20to%20api%2018sept2019%20edits%2018dec2019.pdf [accessed 17 January 2022].

69. W. Michelle, B. Špela, H. Lucia, H. Jaco, E. Johanna, N. L. Amund, L. S. Maria, S. Kristine, T. Johan, B. Kees, W. Patrick, M. Paul, S. László, L. Pascal, M. José, H. Zoltan, K. János, S. Katalin, H. Katarina, V. Andrej, Technical Guideline Tools for harmonization of data collection on Batteries (ORAMA Consortium, 2020); https://orama-h2020.eu/wp-content/uploads/ORAMA_WP4-1_Guidance_To_Data_Harmonization_For_SRM_for_Batteries.pdf [accessed 17 January 2022].

70. C. Donald, E. Emma, S. Shriram, Automotive Lithium-ion Cell Manufacturing: Regional Cost Structures and Supply Chain Considerations [Clean Energy Manufacturing Analysis Center (CEMAC), 2016]; https://www.nrel.gov/docs/fy16osti/66086.pdf [accessed 15 January 2022].

71. FACT SHEET: Securing a Made in America Supply Chain for Critical Minerals (The White House, 2022); https://www.whitehouse.gov/briefing-room/statements-releases/2022/02/22/fact-sheet-securing-a-made-in-america-supply-chain-for-critical-minerals/.

72. A.-L. Toba, R. T. Nguyen, C. Cole, G. Neupane, M. P. Paranthaman, U.S. lithium resources from geothermal and extraction feasibility. *Resour. Conserv. Recycl.* **169**, 105514 (2021).

73. Iron and Steel [International Energy Agency (IEA), 2021]; https://www.iea.org/reports/iron-and-steel [accessed 17 January 2022].

74. Labour cost levels by NACE Rev. 2 activity (Eurostat, 2021); https://ec.europa.eu/eurostat/cache/metadata/de/lc_lci_lev_esms.htm [accessed 17 January 2022].

75. Statistics, Wage & Working Hours [Ministry of the Employment and Labor (South Korea), 2019]; https://www.moel.go.kr/english/resources/statistics.do [accessed 17 January 2022].

76. Statistical Yearbook of Japan 2020 (Statistics Bureau Ministry of Internal Affairs and Communications Japan, 2021); https://www.stat.go.jp/english/data/nenkan/69nenkan/index.html.

77. International Comparison Program (ICP) (World Bank, 2017); https://www.worldbank.org/en/programs/icp/data [accessed 17 January 2022].

78. International construction market survey 2021 (Turner & Townsend, 2021); https://ontarioconstructionnews.com/wp-content/uploads/2021/07/798439_international-construction-market-survey-2021-web.pdf [accessed 17 January 2022].

79. R. E. Ciez, J. F. Whitacre, Comparison between cylindrical and prismatic lithium-ion cell costs using a process based cost model. *J. Power Sources* **340**, 273–281 (2017).

80. LME Nickel [The London Metal Exchange (LME), 2022]; https://www.lme.com/en/metals/non-ferrous/lme-nickel [accessed 05 April 2022].

81. R. E. Ciez, J. F. Whitacre, Examining different recycling processes for lithium-ion batteries. *Nat. Sustain.* **2**, 148–156 (2019).

82. Occupational Employment and Wage Statistics (U.S. Bureau of Labor Statistics, 2020); https://www.bls.gov/oes/oes_emp.htm [accessed 17 January 2022].

83. C. Ian, Z. Yvonne, W. John, W. Jeff, R. Justin, M. Lars, China's Next Leap in Manufacturing (Boston Consulting Group, 2018); https://www.bcg.com/publications/2018/china-next-leap-in-manufacturing [accessed 17 January 2022].

84. Energy Storage Grand Challenge Roadmap [U.S. Department of Energy (DOE), 2020]; https://www.energy.gov/sites/default/files/2020/12/f81/Energy%20Storage%20Grand%20Challenge%20Roadmap.pdf [accessed 15 January 2022].

85. D. Yue, F. You, S. B. Darling, Domestic and overseas manufacturing scenarios of silicon-based photovoltaics: Life cycle energy and environmental comparative analysis. *Solar Energy* **105**, 669–678 (2014).

86. K. Iwata, "Climate Policy in Transportation Sector: Role of Carbon Pricing" in Carbon Pricing in Japan, T. H. Arimura, S. Matsumoto, Eds. (Springer, 2021), pp. 61–78.

87. State and Trends of Carbon Pricing 2020 (World Bank, 2020); https://openknowledge.worldbank.org/entities/publication/bcc20088-9fbf-5a71-8fa0-41d871df4625 [accessed 17 January 2022].

88. Global EV Outlook 2021 [International Energy Agency (IEA), 2021]; https://www.iea.org/reports/global-ev-outlook-2021.

89. State and Trends of Carbon Pricing 2019 (World Bank, 2019); https://openknowledge.worldbank.org/entities/publication/0a107aa7-dcc8-5619-bdcf-71f97a8909d6 [accessed 17 January 2022].

90. D. J. Beerling, E. P. Kantzas, M. R. Lomas, P. Wade, R. M. Eufrasio, P. Renforth, B. Sarkar, M. G. Andrews, R. H. James, C. R. Pearce, J.-F. Mercure, H. Pollitt, P. B. Holden, N. R.

Edwards, M. Khanna, L. Koh, S. Quegan, N. F. Pidgeon, I. A. Janssens, J. Hansen, S. A. Banwart, Potential for large-scale CO_2 removal via enhanced rock weathering with croplands. *Nature* **583**, 242–248 (2020).

91. Y.-M. Wei, J.-N. Kang, L.-C. Liu, Q. Li, P.-T. Wang, J.-J. Hou, Q.-M. Liang, H. Liao, S.-F. Huang, B. Yu, A proposed global layout of carbon capture and storage in line with a 2 °C climate target. *Nat. Clim. Chang.* **11**, 112–118 (2021).

92. Á. Galán-Martín, D. Vázquez, S. Cobo, N. Mac Dowell, J. A. Caballero, G. Guillén-Gosálbez, Delaying carbon dioxide removal in the European Union puts climate targets at risk. *Nat. Commun.* **12**, 6490 (2021).

93. D. W. Keith, G. Holmes, D. St Angelo, K. Heidel, A process for capturing CO_2 from the atmosphere. *Joule* **2**, 1573–1594 (2018).

94. R. Socolow, M. Desmond, R. Aines, J. Blackstock, O. Bolland, T. Kaarsberg, N. Lewis, M. Mazzotti, A. Pfeffer, K. Sawyer, J. Siirola, B. Smit, J. Wilcox, *Direct Air Capture of CO2 with Chemicals: A Technology Assessment for the APS Panel on Public Affairs* (2011).

95. C. Hepburn, E. Adlen, J. Beddington, E. A. Carter, S. Fuss, N. Mac Dowell, J. C. Minx, P. Smith, C. K. Williams, The technological and economic prospects for CO_2 utilization and removal. *Nature* **575**, 87–97 (2019).

96. F. Matthias, Cost and Performance of Carbon Dioxide Capture from Power Generation (International Energy Agency (IEA), 2011) [accessed 17 January 2022].

97. J. Lucas, L. Amy, W. Elizabeth, F. S. Julio, P. H. Steven, B. Rafael, Microsoft's million-tonne CO_2-removal purchase — Lessons for net zero. *Nature* **597**, 629–632 (2021).
